# Teaching medicine web-based with the help of interactive audience response systems

**DOI:** 10.1371/journal.pone.0289417

**Published:** 2023-08-15

**Authors:** Phillip Kremer, Leonard Richter, Leander Melms, Claus F. Vogelmeier, Juergen R. Schaefer

**Affiliations:** 1 Center for Unknown and Rare Diseases, UKGM GmbH, University Clinic Marburg and Philipps-University, Marburg, Germany; 2 University Medical Center Hamburg-Eppendorf, University Heart and Vascular Center, Cardiology, Martinistraße, Hamburg, Germany; 3 UKGM, Department of Internal Medicine, Pulmonary and Critical Care Medicine, German Center for Lung Research (DZL), Baldingerstr, Marburg, Germany; Alexandria Medicine: Alexandria University Faculty of Medicine, EGYPT

## Abstract

The COVID-19 pandemic confronted the medical community worldwide with numerous challenges, not only with respect to medical care, but also for teaching the next generation of physicians. To minimize the risk of infections patient-unrelated classes can be held digitally. Here we present a student initiated, web-based teaching approach, called “From symptom to diagnosis”. In this seminar case reports of rare diseases were presented to the audience in a symptom-focused manner. The patients´ most significant symptoms were presented, followed by an in-depth discussion about differential diagnosis. First glance diagnosis pictures were shown to improve students´ ability to identify important clinical scenarios. We used chat functions as well as an audience response system to make the seminar more interactive. By this we attracted between 71 and 147 participants per session. The online seminar was very well perceived and 97% of the students saw an improvement of their diagnostic skills. In summary, we successfully established an interactive, web-based teaching format for medical students.

## Introduction

The COVID-19 pandemic confronted the medical community with unexpected challenges. The nationwide lockdown and the need for social restrictions to reduce the spread of SARS-CoV-2 had an impact on all aspects of life including medical education [1–3]. Traditionally, medical training is strongly based on in-person learning with an increasing number of practical courses as the students become more experienced [4, 5]. Besides, students must participate in lectures and seminars, which usually take place within the hospital. However, the COVID-19 pandemic stopped the bedside and in-class teaching in numerous medical schools and medical training had to be transferred into a digital format [6–10]. Several models of online educational activities like video tutorials as well as video surgeries have been established [11]. Nonetheless, the pandemic took this web-based learning on a new level. Early data indicate that medical students appreciate well performed, online based lectures [12, 13]. For this we hypothesized that utilizing an audience response system (ARS) might improve online teaching substantially. The ARS technology is a recent innovation, which provides a mechanism to engage larger groups as active participants in teaching sessions. Originally developed for in face-to-face teaching, ARS now plays a pivotal role in web-based teaching formats, as they turn passively listening students into active online participants. These systems can increase interactivity and improve learning outcomes [14]. However, the potential of ARS for web-based medical courses is currently unknown. With the online seminar “From symptom to diagnosis” we continued our effort to improve the medical education for rare diseases by focusing on the role of anamnesis, clinical history and physical findings [15–17]. We developed an innovative, web-based teaching format focused on teaching rare diseases under pandemic conditions [18]. The aim of our project is to attract students for a better understanding of rare diseases by utilizing modern web-based, interactive teaching techniques.

## Materials and methods

### Materials

We used BigBlueButton which is an open-source web conferencing system. It was designed for online education and provides a variety of presentational tools. The presenter can share audio, webcams, slides, and screen with his or her students, which are key features in online lessons. Participants can either use the chat tool to ask questions or their microphones to ask the educator directly. Lecturers can create different rooms and then share the link with their students, who will enter the meeting. The access to this platform is free of charge by the Philipps-University of Marburg.

The Audience Response System Poll Everywhere® was purchased for this course offering a maximum audience size of 700 participants. The tool allows us to create an unlimited number of questions. Poll Everywhere® permits different online activities such as: multiple-choice questions, word clouds, Q&As, clickable images, surveys, open-ended questions, competition-based questions, donut charts, upvoting-based questions, emotion scale, presentation feedback, spotlight, retrospective, discussion, brainstorming, select on map, bulletin board, 2x2 matrix. Many settings can be adjusted, like the number of answers a participant can give (one or multiple per question), and the time provided for the participants to answer.

### Design of the seminars

The course is offered to students every two weeks (Tuesdays from 6:00 p.m. to 7:30 p.m.) as a web-based lecture on the university’s own online platform (BigBlueButton) as a voluntary seminar. Medical students (PK & LR), both in their second last or last year, prepared a case report with the support of experienced clinicians. The support of the full professors was limited on suggesting clinical cases, preparing the seminar, and answering highly specific questions before or during the session. However, the online classes were given by the students themselves. At the beginning of each seminar, five multiple-choice questions relating to topics of the past seminar were posted to check the learning effect. Thereafter, the presentation of a new case followed. The presented case was either from our own “Centre for undiagnosed and rare diseases” (ZusE), Marburg University Hospital or taken from the literature (such as case reports published in the New England Journal of Medicine, The Lancet or other medical journals). The case was presented step by step (history, physical findings, laboratory studies, imaging and further diagnostic tests). The most relevant symptoms were discussed in detail with the participating students. A special focus was on potential differential diagnoses. The students used both, the system´s chat function as well as the ARS "Poll Everywhere®" with great enthusiasm. During a seminar the ARS was used about 15 to 20 times. After the diagnosis was made the underlying pathophysiology, therapy and clinical course were explained. The seminar ended by presenting a series of clinical pictures to train their “clinical view”. Goal of our first glance diagnosis training was to teach students the most important diagnoses that every clinician regardless of her/his specialization must know. The seminar was continuously evaluated by the participants.

### Ethical approval

A formal waiver of ethical approval was granted by the Institutional Review Board (Ethic Committee Marburg) since this paper analyses voluntarily and anonymously received standard evaluation data from our medical students retrospectively and does not involve any studies in humans (23–81 Anz Schaefer).

### Evaluation and statistical analysis of the seminar series

**Fig 1:**
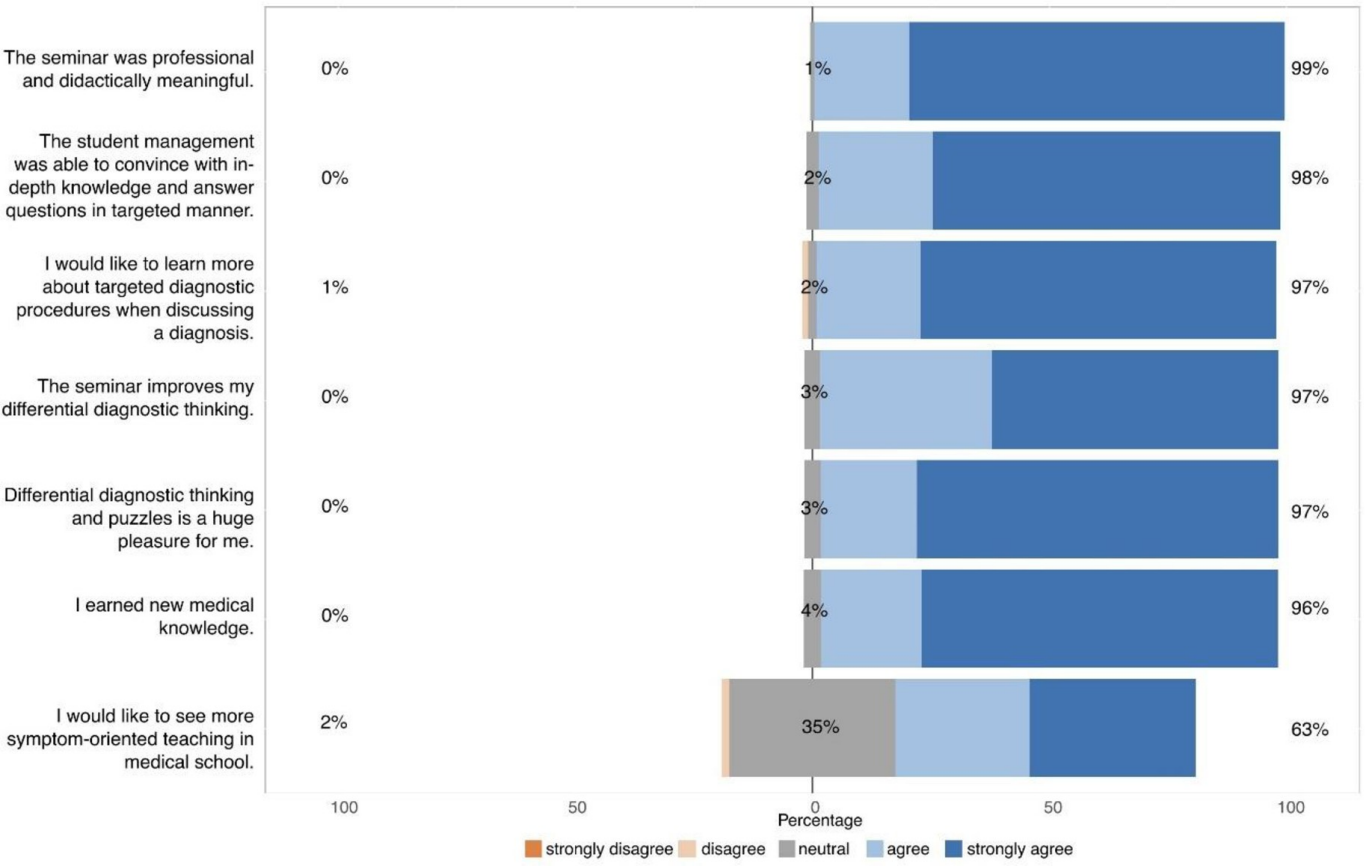
Motivation, overall impression und learning effect of the medical students after participating the course „From Symptom to Diagnosis” (n = 164).

**Fig 2:**
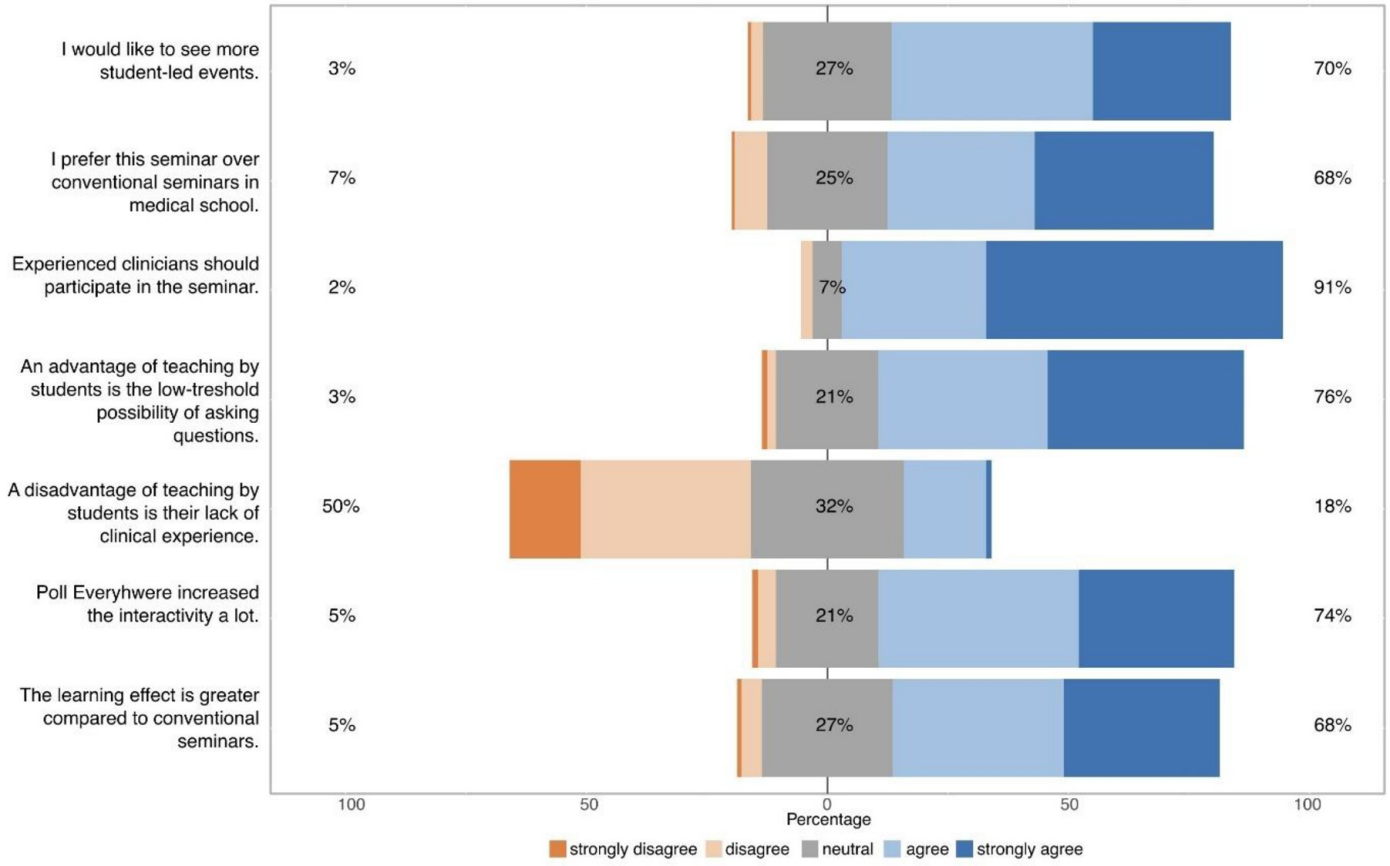
Advantages and disadvantages of a student-led, web-based course in medical school and the usage of an audience response system within this course (n = 165).

This study reports the consecutively generated responses of all students willing to participate in this project. The primary focus of the online questionnaire was to evaluate and improve our teaching approach. There was no predefined or calculated sample size, and all responses are summarized in this report. Data were obtained from six different seminars in comma-separated values format from the questionnaire server. The R programming language was utilized for exploratory data analysis, data post-processing and plotting. Basic data were analyzed using descriptive statistics. For analysis, the Likert questionnaire items were assigned a corresponding point value between 0 and 4 (0  =  strongly disagree, 1  =  disagree, 2  =  neutral, 3 = agree, 4  =  strongly agree) and the centre of the scale is 2. All data are presented as average ± standard deviation, unless otherwise stated. Figs 1 and 2 were generated with the R library likert [19]. As part of this evaluation, an anonymous online–questionnaire designed with Microsoft forms with 47 questions was completed by most of the participants once (see supporting information file).

## Results

The course "From Symptom to Diagnosis" was attended by a total of 658 medical students during six seminars at the Philipps-University in Marburg, Germany (71 to 147 participants per seminar, mean 109.7 ± 25,7).

76% of participating students were clinical semesters and 24% were pre-clinical: 1st year medical studies: 17.0%; 2nd year medical studies: 7.0%; 3rd year medical studies (1st clinical year): 23.0%; 4th year medical studies (2nd clinical year): 29.0%; 5th year medical studies (3rd clinical year): 17.0%; 6th year medical studies (practical year students): 7.0%. 16% of the participants had experience in conducting physical examinations, however the majority (52%) had little or no practice with physical examination. Moreover, 67% of the participants had no experience in choosing diagnostic tests for evaluating a patient. Furthermore, most students (69%) were unfamiliar with critical clinical situations requiring urgent action. 10% of the students had experience assessing laboratory values, 8% had knowledge regarding imaging techniques and 4% of the students were familiar with “first glance diagnosis” analyzing clinical pictures.

### Why do medical students visit the online-seminar?

Most of the students wanted to learn more about diagnostic procedures (96.6%) and to identify pathognomonic clinical pictures (97.5%). Almost the whole audience stated that they enjoyed the event (97.6%) and reported added value regarding professional knowledge (96.4%). Overall, this was achieved through a professional, didactically meaningful seminar (99.2%). Taken together, 99.2% of the participants would recommend this seminar to their fellow students.

### Advantages and disadvantages of a students—teach—students seminar

76% of undergraduates saw the low-threshold possibility of asking questions as an advantage of the student teaching format. Furthermore, 68% of the participants stated a subjectively higher learning effect compared to conventional online seminars. 91% of the students insist in the supervision of the seminar by an experienced member of the faculty (see Fig 1).

Our approach to repeat questions from earlier seminars enabled us to recognize some improvements. For example, a localized linear scleroderma, shown as the classic “En coup de sabre”, was initially recognized by only 9 (= 22%) of the students. With a similar but not the identical picture two weeks later, the correct answer was given by 13 (= 36%) (see Table 1). However, the total number of students answering our questions was low. This might indicate that students who don´t remember the correct answer might simply skip answering.

**Table 1:** Learning effect of students in pathognomonic clinical pictures and essential medical knowledge.

clinical topic	correct answers (1st time)	total answers (1st time)	correct answers (2nd time)	total answers (2nd time)
**En coup de sabre**	9 (22%)	42	13 (36%)	36
**Meigs-Syndrome**	12 (30%)	40	12 (41%)	29
**Definition Anemia**	29 (62%)	47	31 (78%)	40
**Blood transfusion**	31 (52%)	60	31 (65%)	48
**Liquor diagnostic**	31 (54%)	57	31 (72%)	43
**Limbic-encephalitis**	26 (45%)	58	34 (72%)	47

**Fig 3:**
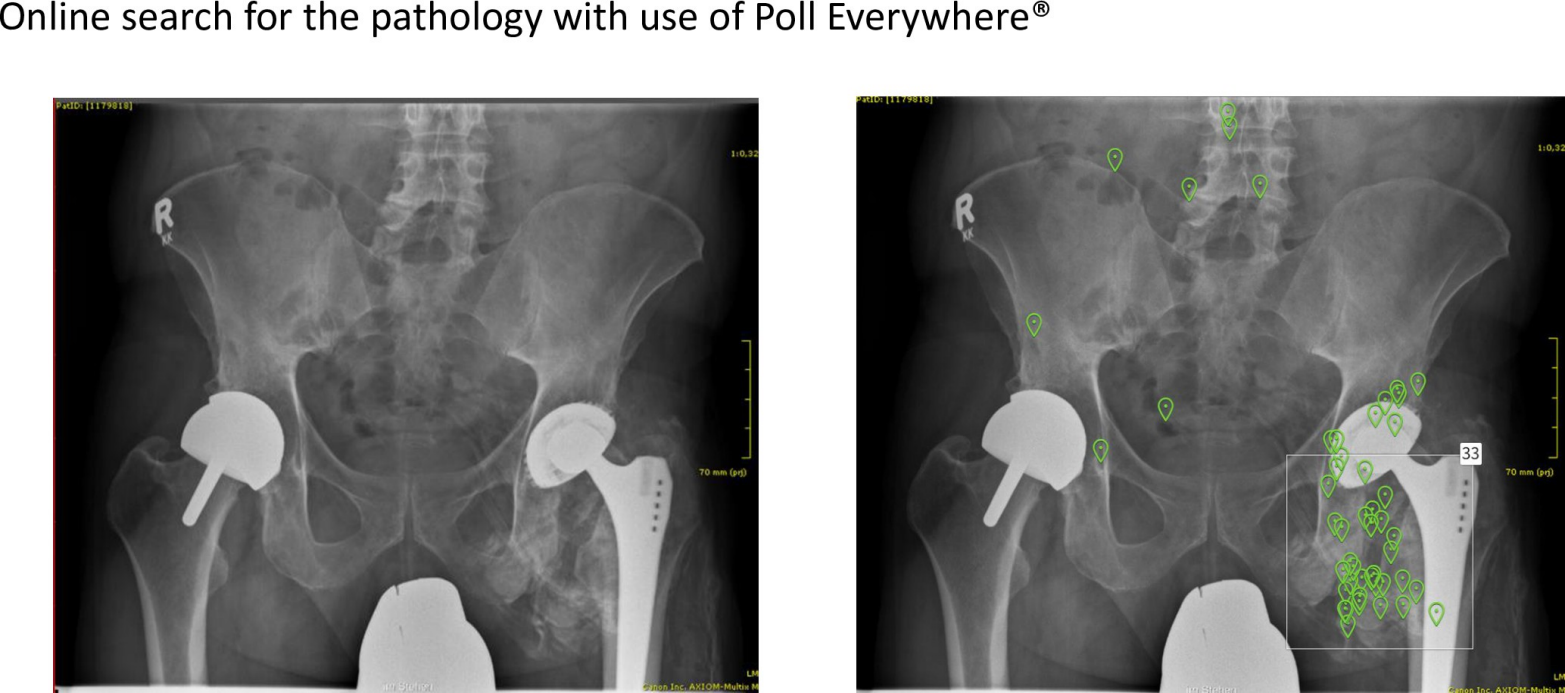
Metallosis in the left hip with typical tissue changes. Live voting with Poll Everywhere®. Green mark corresponds to one student vote. Area of interest is marked with a rectangle. 33 out of 50 (= 66%) students identified the suspected area of metal infiltrated tissue correct (X-ray of the hip by UKGM Radiology Marburg).

Moreover, we showed the students three pictures, which were assembled into one and which should be recognized as the “Meigs syndrome” or the “Pseudo-Meigs syndrome” [20]. Initially 30% of the students selected the correct answer. Two weeks later 41% recognized the typical clinical picture (see Table 1). Anemia is one of the most common problems in clinical practice, up to 10.6% of patients (65 years or older) are diagnosed with anemia [21]. Pivotal for the diagnosis of anemia is the haemoglobin level. This was recognized by 62% of the students, when we first asked for it. Two weeks later the proportion of correct answers improved and 78% knew the definition of anemia. Since dealing with anemia is a quite important and widely spread aspect of medicine, we asked our participating students, how many millilitres of erythrocyte concentrate must be transfused to increase haemoglobin levels by 1g/dl. Initially 52% of the students chose the correct answer (one unit of blood transfusion or 300ml) [22, 23]. In the second query two weeks later, 65% of the students knew the correct answer. Similar results were obtained with multiple other questions (see Table 1). Nonetheless, this survey of evaluating students learning improvements has several limitations. Answering questions was not mandatory. Moreover, the total number of participants was consistently higher within the first round of questions. After two weeks, when we showed slightly modified questions, less students participated. However, of those answering, the percentage of correct answers was always higher. By this it is conceivable that mostly those students who were sure of the correct answer took part and those who had no idea did not.The ASR Poll Everywhere®, used in this course, offered the possibility to show different clinical images, like histological specimens, X rays, CT or MR-scans, dermatologic findings, and other clinical findings. As an example, we showed a hip radiogram with tissue metallosis due to a cobalt intoxication [24]. In the next step, students were asked to flag the suspected underlying pathology within the picture (see Fig 3) and 66% (33 of 50) marked the suspected area correctly. By utilizing these options, a total of 95.8% of our students stated a significant increase in interaction using the Poll Everywhere® tool.

## Discussion

The COVID-19 pandemic challenged the medical systems but also the educational system worldwide. It forced us to develop new and innovative teaching practices [1, 2, 18, 25]. Due to the need for social distancing, many well-established practical courses were discontinued [7]. Medical student education rapidly shifted to a virtual format in the areas where remote learning was possible [6–9].

Case-based learning is well- and long-established teaching approach for medical students. Although, often claimed as a highly effective teaching method, there is no clear and conclusive evidence that it is more effective compared to other methods [26]. Our teaching approach (modified case-based learning) is strongly focused on the patient’s symptoms as the crucial part of the diagnostic process. This compares to a medical puzzle and improves the differential diagnostic thinking of our students. Similar findings, especially having fun by solving rare and difficult clinical cases, was generated by our seminar called “Teaching medicine with the help of Dr. House” [15]. Unfortunately, the broad use of “Dr. House” video clips via internet is not permitted, preventing such teaching strategies as an online version during the pandemic.

The diagnostic process always starts with the patient’s medical history, followed by in-depth physical examination with focus on the symptoms presented [16, 17]. After the initial assessment of patient’s history and the physical exam, the physician must choose from a plethora of further diagnostic tests to find the correct diagnosis. Our teaching approach followed this real-life scenario and allowed the students to understand the decisions made by experienced physicians in detail. Clearly, this symptom-focused approach is more interesting for our students compared to the organ-focused approach. However, this approach requires a detailed knowledge of different diseases which is not always present in the earlier stage of study.

Web-based teaching is usually less interactive. Therefore, some fundamental concepts must be considered. First, in contrast to traditional education online lectures are characterized by physical distance. Second, students must be able to give feed-back, to share their thoughts on topics, attend in discussions or queries, but also project their feeling and questions into the audience [27]. The digital teaching format enables many students (here up to 150) to participate in events, which would otherwise be limited by spatial requirements and the more difficult interaction. To overcome this interactivity problem, we used the ARS Poll Everywhere®. ARS have been used to increase effectiveness regarding learning outcomes and to create interactivity between the presenter and its audience [14, 28].

A systematic review by Nelson et al. (2012) evaluated twenty-one studies, involving 2637 participating, whether the use of an ARS results in greater knowledge and self-confidence improvements compared to traditional lectures [14]. Fourteen of those studies reported a statistically significant difference in utilized knowledge scores, favoring the use of an ARS with greater difference in non-randomized studies. In 10 non-randomized trials the immediate knowledges scores were higher in the ARS groups (95% CI 1.47). Overall, there was a modest benefit of an ARS in terms of knowledge and self-confidence improvements. In line with the findings by Nelson et al., Hussain et al. (2019) assessed the outcomes of using an ARS in pharmacy education with a systematic review of eleven studies [29]. Participating students reported positive impact on participation and engagement. It was shown that the use of an ARS seems to enhance active learning. However, impact of ARS on course grades showed mixed results [29, 30]. Douce et al. (2009) conducted a prospective observational controlled study to evaluate, whether the use of an ARS would promote an active learning environment during case-based discussions in large groups [31]. In their study one group A (N = 83) answered open-ended question, for the other group B (N = 86) an ARS was used to poll students’ individual responses. Although there were significant improvements in final examination results (group A 89.0% ± 11.9 and group B 92.2% ± 5.4, *p* = 0.03), the long-term retention after one year was not significantly different (group A 51.0% ± 8.5 and group B 51.3% ± 10.0, *p* = 0.89). However, the authors showed that ARS use increased student engagement and significantly improved the learning experience. Moreover, a case-study by Cain et al (2009) showed that using an ARS increases attention of students during in-class lectures, which was reported by 98% students [32]. This rise in attention may be even more important in a web-based seminar compared to in-class teaching. These studies are consistent with our findings that using an ARS is well accepted by participating students, increases interactivity within the seminar and can enhance the learning effect of medical knowledge.

Despite all the positive aspects of an ARS, there are some drawbacks. First, trainees working with an ARS have to learn and set up the technology [33]. Next, it is necessary to create precise questions, which provide a benefit for the users and last, the audience must be able to answer questions directly and specifically [33, 34]. This can be achieved by implementing multiple-choice questions or by providing questions whose answers are collected in a word cloud, followed by a detailed discussion with the audience. The need to formulate the question precise and short, requires from our students the close and thoughtful follow-up of the presentation. It is important to anticipate the answers of the audience beforehand. By this the next presentation slide will be in line with answers from the previous ARS question. Studies from universities in the USA and Saudi Arabia showed great potential to use an ARS for teaching medical students, especially since online lectures will still be useful after the peak of the COVID-19 pandemic [35]. Castillo et al (2020) studied the effect of an ARS in respect to learner satisfaction in a group of internal medicine fellowships (n = 18). By using a four-question retrospective pre-post satisfaction survey with a standardized five-item Likert scale the authors showed that incorporation of the ARS (Poll Everywhere®) resulted in a shift towards more favorable satisfaction scores and self-perceived attentiveness compared to the pre-intervention responses. [28].

Although, most of us are looking forward to “normal” face-to-face teaching, a digital teaching format offers additional, far-reaching chances to improve lecturing also interactively. However, since teaching is gradually returning to a pre-corona phase, the question arose whether an online-based seminar may play a role in the future. During the pandemic many students missed interaction with their classmates and were critical of a purely online-based teaching. In addition, practical skills cannot be learned virtually and purely web-based teaching may miss important clinical skills [12, 36, 37]. Nevertheless, it is undoubtedly an advantage that an online format does not require physical presence, can be timesaving and even allows students around the globe to participate in seminars such as “From Symptom to diagnosis” [35].

This study has several limitations. First, this is a single centre, retrospective, uncontrolled study. By this there is a potential sampling bias and the generalizability of our findings is limited. The overall number of students, who completed our survey is small and there is a great variability regarding the students from all pre-clinical and clinical years. Since our survey was completely anonymous, there is the possibility that–despite our request not to do so—some students might have completed it more than once, whereas others did not conduct the survey at all.

In summary we could show that web-based teaching is feasible and effective. A symptom-based approach with a focus on differential diagnoses and rare diseases is well received by medical students.

## Supporting information

S1 File (PDF)

S2 File (PDF)

S3 File (PDF)
