# Supplementary material for: Teaching medicine web-based with the help of interactive audience response systems
Source: PLoS One. 2023 Aug 15;18(8):e0289417. doi: 10.1371/journal.pone.0289417 (PMC10427006; doi:10.1371/journal.pone.0289417)
Supplement: S1 File — (PDF) [file pone.0289417.s001.pdf]

Evaluation of the seminar "From Symptom to diagnosis"

Das Ausfüllen der Umfrage dauert ungefähr 7 Minuten.

* Erforderlich

1. Which study year are you currently attending? *

- ○ 1
- ○ 2
- ○ 3
- ○ 4
- ○ 5
- ○ 6
- ○ Sonstiges

2. How many internships have you finished currently? *

- ○ 1
- ○ 2
- ○ 3
- ○ 4
- ○ 5
- ○ 6
- ○ more than 6
- ○ Keine
- ○ Sonstiges

3. What are reasons for visiting the seminar "From Symptom to diagnosis"?

	Strongly agree	Agree	Neutral	Disagree	Strongly disagree
The content is relevant to my education.	○	○	○	○	○
For me, the timing, early evening, is pleasant.	○	○	○	○	○
To learn more about targeted diagnostic procedures when discussing a diagnosis.	○	○	○	○	○
To get to know more diagnostic options.	○	○	○	○	○
To discover new diseases.	○	○	○	○	○
The seminar is fun.	○	○	○	○	○
I learned a lot.	○	○	○	○	○
I learned new stuff.	○	○	○	○	○
I would like to learn clinical important visual diagnoses.	○	○	○	○	○
Differential diagnostic thinking and "puzzles" give me pleasure.	○	○	○	○	○

4. How experienced are you with...

	None	Rarely	Some	Frequently	Very frequently
With conducting a clinical examination.	○	○	○	○	○
With diagnostic algorithms in clinical settings.	○	○	○	○	○
The assessment of imaging (CT/MRI/X-ray).	○	○	○	○	○
The solution of so-called eye-diagnoses pictures.	○	○	○	○	○
The assessment of which diagnostics should be chosen in the clinic at the beginning.	○	○	○	○	○
The assessment of challenging patient situations that require urgent action.	○	○	○	○	○

5. What is your overall impression of the course?

	Strongly agree	Agree	Neutral	Disagree	Strongly disagree
The claim of the event is reasonable.	◯	◯	◯	◯	◯
Attending the course was associated with a learning success for me.	◯	◯	◯	◯	◯
I would rate the selection and solvability of the case as good.	◯	◯	◯	◯	◯
The event makes it easier for me to assign individual symptoms to clinical pictures.	◯	◯	◯	◯	◯
Learning visual diagnoses can be beneficial for later clinical practice.	◯	◯	◯	◯	◯
I would rate the solvability of the eye-diagnoses as good or feasible.	◯	◯	◯	◯	◯
I would recommend the event to my fellow students.	◯	◯	◯	◯	◯
The event doesn't have much added value for me.	◯	◯	◯	◯	◯
The duration of the event is reasonable.	◯	◯	◯	◯	◯
I would like the event to become part of curricular teaching.	◯	◯	◯	◯	◯
The student management was able to convince with in-depth knowledge and answer questions in a targeted manner.	◯	◯	◯	◯	◯
The seminar was professional and didactically meaningful.	◯	◯	◯	◯	◯

6. To what extent do the following statements apply to you?

	Strongly agree	Agree	Neutral	Disagree	Strongly disagree	Agree
During the seminar it was easy for me to concentrate throughout.						
I am in favour of the fact that relevant content is taken up and explained thematically in the seminar.						
After participating in this event, I noticed that teaching based on the symptom is not adequately teached in medical school.						
I have already been able to learn new clinical pictures and visual diagnoses.						
The seminar makes it easier for me to judge which differential diagnoses are suitable on the basis of various symptoms.						
Learning symptoms or symptom constellations promotes my differential diagnostic thinking.						

7. How would you rate the use of the "Poll everywhere" tool?

	Strongly agree	Agree	Neutral	Disagree	Strongly disagree
The tool made the seminar much more interactive.					
I would like to continue using the tool.					
I would like to see it used in other events as well.					
I would prefer a seminar without the use of additional tools.					

8. What are the advantages and disadvantages of a student-led seminar?

	Trifft sicher zu	Trifft zu	Unentschieden	Trifft nicht zu	Trifft sicher nicht zu
One advantage of teaching by students is the low-threshold possibility of asking questions, which many professors tend to be inhibited about.	○	○	○	○	○
A disadvantage of teaching by students is their lack of professional experience and that the course content is read and, at best, discussed by the lecturer during preparation.	○	○	○	○	○
In order to compensate for the lack of clinical experience, we will always have a "veteran clinician" with us at the seminars. Is this helpful from your point of view?	○	○	○	○	○
I would like to see more student-led events.	○	○	○	○	○
The student leadership makes it easier for me to interact.	○	○	○	○	○
I would prefer attending this event over conventional seminars.	○	○	○	○	○
Compared to conventional seminars, this event is associated with a greater learning effect for me.	○	○	○	○	○

9. Any other feedback you'd like to share with us?

Dieser Inhalt wurde von Microsoft weder erstellt noch gebilligt. Die von Ihnen übermittelten Daten werden an den Formulareigentümer gesendet.

Microsoft Forms
