# Supplementary material for: Teaching medicine web-based with the help of interactive audience response systems
Source: PLoS One. 2023 Aug 15;18(8):e0289417. doi: 10.1371/journal.pone.0289417 (PMC10427006; doi:10.1371/journal.pone.0289417)
Supplement: S2 File — (PDF) [file pone.0289417.s002.PDF]

ID	Startzeit	Fertigstellungszeit	E-Mail
2	11.9.21 19:53:10	11.9.21 19:55:56	anonymous
3	11.9.21 19:53:23	11.9.21 19:56:04	anonymous
4	11.9.21 19:53:34	11.9.21 19:56:06	anonymous
5	11.9.21 19:53:26	11.9.21 19:56:20	anonymous
6	11.9.21 19:53:24	11.9.21 19:56:21	anonymous
7	11.9.21 19:53:22	11.9.21 19:56:27	anonymous
8	11.9.21 19:53:36	11.9.21 19:56:35	anonymous
9	11.9.21 19:53:34	11.9.21 19:57:03	anonymous
10	11.9.21 19:53:45	11.9.21 19:57:06	anonymous
11	11.9.21 19:53:39	11.9.21 19:57:11	anonymous
12	11.9.21 19:53:29	11.9.21 19:57:17	anonymous
13	11.9.21 19:54:41	11.9.21 19:57:25	anonymous
14	11.9.21 19:53:20	11.9.21 19:57:27	anonymous
15	11.9.21 19:53:19	11.9.21 19:57:27	anonymous
16	11.9.21 19:53:45	11.9.21 19:57:28	anonymous
17	11.9.21 19:53:19	11.9.21 19:57:28	anonymous
18	11.9.21 19:53:44	11.9.21 19:57:33	anonymous
19	11.9.21 19:54:03	11.9.21 19:57:37	anonymous
20	11.9.21 19:53:34	11.9.21 19:57:41	anonymous
21	11.9.21 19:53:35	11.9.21 19:58:11	anonymous
22	11.9.21 19:53:36	11.9.21 19:58:16	anonymous
23	11.9.21 19:53:29	11.9.21 19:58:38	anonymous
24	11.9.21 19:54:41	11.9.21 19:58:52	anonymous
25	11.9.21 19:55:05	11.9.21 19:59:17	anonymous
26	11.9.21 19:53:27	11.9.21 19:59:30	anonymous
27	11.9.21 19:55:02	11.9.21 19:59:47	anonymous
28	11.9.21 19:54:01	11.9.21 19:59:52	anonymous
29	11.9.21 19:54:37	11.9.21 19:59:57	anonymous
30	11.9.21 19:53:21	11.9.21 20:00:00	anonymous
31	11.9.21 19:55:02	11.9.21 20:00:23	anonymous
32	11.9.21 19:54:36	11.9.21 20:00:33	anonymous
33	11.9.21 19:54:36	11.9.21 20:00:41	anonymous
34	11.9.21 19:54:41	11.9.21 20:00:52	anonymous
35	11.9.21 19:53:25	11.9.21 20:01:55	anonymous
36	11.9.21 19:53:51	11.9.21 20:02:19	anonymous
37	11.9.21 19:53:21	11.9.21 20:02:31	anonymous
38	11.9.21 19:53:29	11.9.21 20:03:05	anonymous
39	11.9.21 19:53:39	11.9.21 20:03:24	anonymous
40	11.9.21 19:53:43	11.9.21 20:04:04	anonymous
41	11.9.21 19:54:48	11.9.21 20:04:33	anonymous
42	11.9.21 19:53:30	11.9.21 20:09:56	anonymous
43	11.9.21 19:53:21	11.9.21 20:43:44	anonymous
44	11.9.21 19:56:34	11.9.21 21:05:53	anonymous
45	11.9.21 21:46:46	11.9.21 21:51:00	anonymous
46	11.9.21 21:52:42	11.9.21 21:56:42	anonymous
47	11.9.21 23:01:13	11.9.21 23:06:51	anonymous
48	11.23.21 17:56:31	11.23.21 18:01:51	anonymous
49	11.23.21 17:59:37	11.23.21 18:04:43	anonymous
50	11.23.21 18:01:08	11.23.21 18:07:43	anonymous

51	11.23.21 18:05:38	11.23.21 18:11:34	anonymous
52	11.23.21 18:05:40	11.23.21 18:36:47	anonymous
53	11.23.21 17:59:48	11.23.21 18:46:30	anonymous
54	11.23.21 18:07:43	11.23.21 19:03:37	anonymous
55	11.23.21 18:04:16	11.23.21 19:12:01	anonymous
56	11.23.21 17:57:45	11.23.21 19:14:40	anonymous
57	11.23.21 19:10:54	11.23.21 19:18:40	anonymous
58	11.23.21 17:59:33	11.23.21 19:37:23	anonymous
59	11.23.21 18:06:43	11.23.21 19:37:43	anonymous
60	11.23.21 19:35:03	11.23.21 19:37:52	anonymous
61	11.23.21 19:35:09	11.23.21 19:38:00	anonymous
62	11.23.21 17:59:46	11.23.21 19:38:28	anonymous
63	11.23.21 19:35:01	11.23.21 19:38:39	anonymous
64	11.23.21 19:35:44	11.23.21 19:38:43	anonymous
65	11.23.21 19:34:59	11.23.21 19:38:45	anonymous
66	11.23.21 19:35:30	11.23.21 19:38:52	anonymous
67	11.23.21 19:35:05	11.23.21 19:39:00	anonymous
68	11.23.21 18:25:28	11.23.21 19:39:00	anonymous
69	11.23.21 19:35:30	11.23.21 19:39:44	anonymous
70	11.23.21 19:35:06	11.23.21 19:40:05	anonymous
71	11.23.21 18:06:43	11.23.21 19:40:06	anonymous
72	11.23.21 17:58:56	11.23.21 19:40:41	anonymous
73	11.23.21 19:35:03	11.23.21 19:40:41	anonymous
74	11.23.21 19:35:09	11.23.21 19:40:42	anonymous
75	11.23.21 19:35:34	11.23.21 19:40:42	anonymous
76	11.23.21 19:35:01	11.23.21 19:40:58	anonymous
77	11.23.21 17:58:05	11.23.21 19:41:09	anonymous
78	11.23.21 19:35:36	11.23.21 19:41:28	anonymous
79	11.23.21 19:35:11	11.23.21 19:41:38	anonymous
80	11.23.21 19:35:33	11.23.21 19:41:49	anonymous
81	11.23.21 19:35:06	11.23.21 19:41:56	anonymous
82	11.23.21 19:35:17	11.23.21 19:42:27	anonymous
83	11.23.21 19:35:14	11.23.21 19:43:18	anonymous
84	11.23.21 18:39:11	11.23.21 19:43:30	anonymous
85	11.23.21 19:35:06	11.23.21 19:43:56	anonymous
86	11.23.21 18:06:40	11.23.21 19:46:09	anonymous
87	11.23.21 19:35:15	11.23.21 20:00:18	anonymous
88	11.23.21 17:56:56	11.23.21 20:45:44	anonymous
89	11.23.21 22:27:53	11.23.21 22:32:11	anonymous
90	11.24.21 0:13:13	11.24.21 0:16:50	anonymous
91	12.7.21 17:59:12	12.7.21 19:29:03	anonymous
92	12.7.21 19:26:35	12.7.21 19:31:06	anonymous
93	12.7.21 17:58:09	12.7.21 19:32:04	anonymous
94	12.7.21 19:26:38	12.7.21 19:33:26	anonymous
95	12.7.21 19:27:30	12.7.21 19:35:13	anonymous
96	12.7.21 19:28:51	12.7.21 19:35:17	anonymous
97	12.7.21 18:04:14	12.7.21 19:36:18	anonymous
98	12.7.21 18:49:36	12.7.21 19:38:05	anonymous
99	12.7.21 19:33:58	12.7.21 19:38:10	anonymous
100	12.7.21 19:27:14	12.7.21 19:38:46	anonymous

101	12.7.21 19:34:29	12.7.21 19:38:53	anonymous
102	12.7.21 19:26:37	12.7.21 19:39:15	anonymous
103	12.7.21 19:27:26	12.7.21 19:39:28	anonymous
104	12.7.21 19:34:45	12.7.21 19:39:42	anonymous
105	12.7.21 19:34:33	12.7.21 19:40:07	anonymous
106	12.7.21 19:33:58	12.7.21 19:40:09	anonymous
107	12.7.21 20:10:15	12.7.21 20:12:45	anonymous
108	12.7.21 20:28:33	12.7.21 20:34:39	anonymous
109	12.7.21 19:26:43	12.7.21 23:09:02	anonymous
110	12.8.21 8:27:26	12.8.21 8:33:56	anonymous
111	12.8.21 13:21:21	12.8.21 13:28:04	anonymous
112	12.8.21 14:01:32	12.8.21 14:07:19	anonymous
113	12.21.21 19:23:59	12.21.21 19:27:02	anonymous
114	12.21.21 19:23:58	12.21.21 19:30:19	anonymous
115	12.21.21 19:23:39	12.21.21 19:36:33	anonymous
116	12.21.21 19:33:29	12.21.21 19:37:02	anonymous
117	12.21.21 19:33:34	12.21.21 19:37:49	anonymous
118	12.21.21 19:24:01	12.21.21 19:38:46	anonymous
119	12.21.21 19:34:13	12.21.21 19:39:35	anonymous
120	12.21.21 19:34:03	12.21.21 19:56:11	anonymous
121	12.21.21 19:56:56	12.21.21 19:59:15	anonymous
122	12.21.21 20:05:35	12.21.21 20:08:04	anonymous
123	12.21.21 20:49:06	12.21.21 20:51:32	anonymous
124	12.22.21 17:16:50	12.22.21 17:21:41	anonymous
125	12.21.21 19:24:02	12.22.21 19:17:30	anonymous
126	1.11.22 19:33:48	1.11.22 19:35:40	anonymous
127	1.11.22 19:34:01	1.11.22 19:37:01	anonymous
128	1.11.22 19:33:58	1.11.22 19:37:09	anonymous
129	1.11.22 19:33:51	1.11.22 19:37:14	anonymous
130	1.11.22 19:34:06	1.11.22 19:37:31	anonymous
131	1.11.22 19:33:54	1.11.22 19:37:50	anonymous
132	1.11.22 19:33:30	1.11.22 19:37:52	anonymous
133	1.11.22 19:34:06	1.11.22 19:38:00	anonymous
134	1.11.22 19:33:51	1.11.22 19:38:25	anonymous
135	1.11.22 19:34:16	1.11.22 19:38:33	anonymous
136	1.11.22 19:34:21	1.11.22 19:38:58	anonymous
137	1.11.22 19:33:54	1.11.22 19:39:02	anonymous
138	1.11.22 19:33:37	1.11.22 19:39:02	anonymous
139	1.11.22 19:34:39	1.11.22 19:39:22	anonymous
140	1.11.22 19:34:32	1.11.22 19:39:25	anonymous
141	1.11.22 19:34:27	1.11.22 19:39:26	anonymous
142	1.11.22 19:34:06	1.11.22 19:39:41	anonymous
143	1.11.22 19:33:54	1.11.22 19:39:49	anonymous
144	1.11.22 19:34:06	1.11.22 19:40:26	anonymous
145	1.11.22 19:33:28	1.11.22 19:41:01	anonymous
146	1.11.22 19:35:16	1.11.22 19:43:28	anonymous
147	1.11.22 19:32:30	1.11.22 19:43:53	anonymous
148	1.11.22 19:33:53	1.11.22 19:47:28	anonymous
149	1.11.22 19:33:40	1.11.22 20:18:08	anonymous
150	1.11.22 19:35:10	1.11.22 21:48:32	anonymous

	151	1.14.22 18:58:41	1.14.22 19:02:50	anonymous
	152	1.25.22 17:55:20	1.25.22 17:58:29	anonymous
	153	1.25.22 17:58:56	1.25.22 18:06:01	anonymous
	154	1.25.22 19:30:16	1.25.22 19:32:40	anonymous
	155	1.25.22 19:30:25	1.25.22 19:34:32	anonymous
	156	1.25.22 19:11:10	1.25.22 19:34:49	anonymous
	157	1.25.22 19:30:17	1.25.22 19:36:03	anonymous
	158	1.25.22 19:36:07	1.25.22 19:39:10	anonymous
	159	1.25.22 19:36:00	1.25.22 19:39:13	anonymous
	160	1.25.22 19:36:24	1.25.22 19:39:39	anonymous
	161	1.25.22 19:35:49	1.25.22 19:39:48	anonymous
	162	1.25.22 19:35:48	1.25.22 19:39:58	anonymous
	163	1.25.22 19:36:06	1.25.22 19:44:00	anonymous
	164	1.25.22 19:36:30	1.25.22 19:44:30	anonymous
	165	1.25.22 19:35:53	1.25.22 19:45:19	anonymous
	166	1.25.22 19:35:50	1.25.22 20:25:33	anonymous
	167	1.25.22 19:30:49	1.25.22 22:06:53	anonymous
	168	2.7.22 9:36:56	2.7.22 9:40:18	anonymous

Name	In welchem Studienjahr	Wie viele Famulaturen h	Die Inhalte sind relevant
	3	3	Trifft zu
	5	6	Trifft sicher zu
	5	3	Trifft sicher zu
	5	3	Trifft sicher zu
	5	1	Trifft sicher zu
	3	0	Trifft sicher zu
	4	2	Trifft sicher zu
	4	2	Trifft sicher zu
	4	2	Trifft sicher zu
	4	2	Trifft zu
	6	4	Trifft sicher zu
	5	4	Trifft sicher zu
	3	Keine	Trifft sicher zu
	5	4	Trifft sicher zu
	4	2	Trifft sicher zu
	3	Im Frühjahr die erste	Trifft sicher zu
	4	1	Trifft sicher zu
	5	1	Trifft zu
	3	1	Trifft sicher zu
	6	5	Trifft sicher zu
	4	1	Trifft sicher zu
	3	0	Trifft sicher zu
	1	0	Trifft sicher zu
	1	0	Trifft sicher zu
	4	2	Trifft sicher zu
	3	0	Trifft sicher zu
	3	0	Trifft sicher zu
	3	0	Trifft sicher zu
	3	0	Trifft sicher zu
	4	1	Trifft sicher zu
	1	Keine	Trifft sicher zu
	4	2	Trifft zu
	4	2	Trifft sicher zu
	4	3	Trifft sicher zu
	4	1	Trifft zu
	4	3	Trifft sicher zu
	2	Keine	Trifft sicher zu
	4	2	Trifft sicher zu
	3	0	Trifft sicher zu
	7	2	Trifft sicher zu
	5	3	Trifft sicher zu
	3	0	Trifft sicher zu
	3	2	Trifft sicher zu
	4	2	Trifft zu
	3	keine	Trifft sicher zu
	4	1	Trifft sicher zu
	5	4	Trifft zu
	1	Keine	Trifft zu
	4	2	Trifft zu

	3	Keine	Trifft zu
	4	1	Trifft sicher zu
	5	3	Trifft sicher zu
	5	3	Trifft sicher zu
	4	3	Trifft sicher zu
	3	Keine	Trifft zu
	2	Keine	Trifft zu
	3	Keine	Trifft sicher zu
	5	3	Trifft sicher zu
	4	1	Trifft sicher zu
	5	4	Trifft sicher zu
	3	Keine	Trifft sicher zu
	1	Keine	Trifft zu
	2	Keine	Trifft zu
	5	5	Trifft sicher zu
	3	Keine	Trifft zu
	6	2	Trifft sicher zu
	3	1	Trifft sicher zu
	1	Keine	Trifft sicher zu
	4	2	Trifft sicher zu
	3	Keine	Trifft sicher zu
	3	Keine	Trifft sicher zu
	4	2	Trifft zu
	3	Keine	Trifft zu
	2	Keine	Trifft sicher zu
	1	Keine	Trifft zu
	1	Keine	Trifft zu
	2	Keine	Trifft sicher zu
	3	1	Trifft sicher zu
	1	Keine	Trifft sicher zu
	4	1	Trifft sicher zu
	1	Keine	Trifft sicher zu
	1	Keine	Trifft sicher zu
	2	Keine	Trifft sicher zu
	1	Keine	Trifft sicher zu
	1	Keine	Trifft sicher zu
	3	Keine	Trifft sicher zu
	1	Keine	Trifft sicher zu
	4	2	Trifft zu
	1	Keine	Trifft sicher zu
	4	Keine	Trifft sicher zu
	4	2	Trifft sicher zu
	1	Keine	Trifft sicher zu
	2	Keine	Trifft zu
	1	Keine	Trifft sicher zu
	3	2	Trifft sicher zu
	6	4	Trifft sicher zu
	3	Keine	Trifft zu
	4	1	Trifft sicher zu
	3	2	Trifft sicher zu

2	Keine	Trifft zu
4	2	Trifft zu
5	3	Trifft zu
3	Keine	Trifft zu
3	Keine	Trifft sicher zu
1	Keine	Trifft sicher zu
5	3	Trifft sicher zu
3	Keine	Trifft sicher zu
5	5	Unentschieden
4	1	Trifft sicher zu
3	Keine	Trifft sicher zu
1	Keine	Trifft sicher zu
3	2	Trifft zu
5	4	Trifft sicher zu
5	4	Trifft sicher zu
5	4	Trifft sicher zu
3	Keine	Trifft sicher zu
1	Keine	Trifft zu
4	1	Trifft sicher zu
3	Keine	Trifft zu
4	3	Trifft sicher zu
6	5	Trifft zu
4	2	Trifft zu
4	1	Trifft sicher zu
6	5	Trifft sicher zu
1	Keine	Trifft zu
2	Keine	Trifft sicher zu
5	4	Trifft sicher zu
1	Keine	Trifft zu
4	2	Trifft sicher zu
1	Keine	Trifft zu
4	2	Trifft zu
5	3	Trifft sicher zu
2	Keine	Trifft zu
1	Keine	Trifft sicher zu
4	2	Trifft sicher zu
4	2	Trifft sicher zu
5	3	Trifft sicher zu
2	Keine	Trifft sicher zu
4	3	Trifft sicher zu
4	2	Trifft sicher zu
6	5	Trifft sicher zu
3	Keine	Trifft zu
4	3	Trifft sicher zu
3	Keine	Trifft zu
5	3	Trifft sicher zu
6	3	Trifft sicher zu
4	1	Trifft sicher zu
5	4	Trifft sicher zu
6	4	Trifft nicht zu

	6	5	Trifft sicher zu
	3	Keine	Trifft zu
	4	1	Trifft sicher zu
	3	2	Trifft sicher zu
	1	Keine	Trifft sicher zu
	6	5	Trifft sicher zu
	4	Keine	Trifft sicher zu
	5	2	Trifft sicher zu
	1	Keine	Trifft sicher zu
	4	1	Trifft sicher zu
	4	2	Trifft sicher zu
	4	2	Trifft sicher zu
	1	Keine	Trifft sicher zu
	4	1	Trifft sicher zu
	5	2	Trifft zu
	1	Keine	Trifft zu
	5	Mehr als 6	Trifft sicher zu
	3	1	Trifft zu

Für mich ist der Zeitpunl	Um mehr über gezieltes	Um mehr diagnostische	Um neue Krankheitsbild
Trifft sicher zu	Trifft zu	Trifft zu	Trifft zu
Trifft zu	Trifft zu	Trifft zu	Trifft sicher zu
Trifft sicher zu	Trifft sicher zu	Trifft sicher zu	Trifft sicher zu
Trifft sicher zu	Trifft zu	Trifft zu	Trifft sicher zu
Trifft sicher zu	Trifft zu	Trifft zu	Trifft sicher zu
Trifft nicht zu	Trifft sicher zu	Trifft sicher zu	Trifft sicher zu
Trifft sicher zu	Trifft sicher zu	Trifft zu	Trifft sicher zu
Trifft zu	Trifft sicher zu	Trifft zu	Trifft sicher zu
Trifft sicher zu	Trifft zu	Trifft zu	Trifft zu
Trifft zu	Trifft zu	Unentschieden	Trifft zu
Trifft zu	Trifft sicher zu	Trifft sicher zu	Trifft sicher zu
Trifft sicher zu	Trifft sicher zu		Trifft zu
Trifft sicher zu	Trifft sicher zu	Trifft sicher zu	Trifft sicher zu
Trifft zu	Trifft sicher zu	Trifft sicher zu	Trifft sicher zu
Trifft sicher zu	Trifft sicher zu	Trifft sicher zu	Trifft sicher zu
Trifft zu	Trifft sicher zu	Trifft sicher zu	Trifft sicher zu
Trifft sicher zu	Trifft sicher zu	Trifft sicher zu	Trifft sicher zu
Trifft zu	Unentschieden	Unentschieden	Unentschieden
Trifft sicher zu	Trifft sicher zu	Trifft sicher zu	Trifft sicher zu
Trifft sicher zu	Trifft sicher zu	Trifft zu	Trifft sicher zu
Trifft sicher zu	Trifft sicher zu	Trifft zu	Trifft zu
Trifft sicher zu	Trifft sicher zu	Trifft sicher zu	Trifft sicher zu
Trifft nicht zu	Trifft sicher zu	Trifft sicher zu	Trifft sicher zu
Trifft zu	Trifft sicher zu	Trifft sicher zu	Trifft sicher zu
Trifft zu	Trifft sicher zu	Trifft sicher zu	Trifft sicher zu
Trifft sicher zu	Trifft zu	Trifft zu	Trifft zu
Trifft zu	Trifft sicher zu	Trifft sicher zu	Trifft sicher zu
Trifft sicher zu	Trifft sicher zu	Trifft sicher zu	Trifft sicher zu
Trifft sicher zu	Trifft zu	Trifft zu	Trifft sicher zu
Trifft sicher zu	Trifft sicher zu	Trifft zu	Trifft sicher zu
Trifft zu	Trifft sicher zu	Trifft sicher zu	Trifft sicher zu
Trifft zu		Unentschieden	Trifft sicher zu
Trifft sicher zu	Trifft sicher zu	Trifft sicher zu	Trifft sicher zu
Trifft sicher zu	Trifft sicher zu	Trifft sicher zu	Trifft sicher zu
Trifft sicher zu	Trifft sicher zu	Unentschieden	Trifft sicher zu
Trifft zu	Trifft sicher zu	Trifft zu	Trifft zu
Trifft sicher zu	Trifft sicher zu	Trifft sicher zu	Trifft sicher zu
Trifft sicher zu	Trifft sicher zu	Trifft sicher zu	Trifft sicher zu
Trifft sicher zu	Trifft sicher zu	Trifft sicher zu	Trifft sicher zu
Trifft nicht zu	Trifft sicher zu	Trifft sicher zu	Trifft sicher zu
Trifft sicher zu	Trifft sicher zu	Trifft sicher zu	Trifft sicher zu
Trifft sicher zu	Trifft sicher zu	Trifft sicher zu	Trifft sicher zu
Trifft sicher zu	Trifft sicher zu	Trifft sicher zu	Trifft sicher zu
Trifft zu	Trifft sicher zu	Trifft zu	Trifft sicher zu
Trifft sicher zu	Trifft sicher zu	Trifft sicher zu	Trifft sicher zu
Trifft sicher zu	Trifft sicher zu	Trifft sicher zu	Trifft sicher zu
Trifft sicher zu	Trifft sicher zu	Trifft zu	Trifft zu
Trifft sicher zu	Trifft sicher zu	Trifft sicher zu	Trifft sicher zu
Trifft sicher zu	Trifft zu	Trifft zu	Trifft zu

Trifft zu	Trifft zu	Trifft zu	Trifft zu
Trifft zu	Trifft sicher zu	Trifft sicher zu	Trifft sicher zu
Trifft nicht zu	Trifft zu	Unentschieden	Trifft zu
Trifft sicher zu	Trifft sicher zu	Trifft sicher zu	Trifft sicher zu
Trifft zu	Trifft sicher zu	Trifft sicher zu	Trifft sicher zu
Trifft sicher zu	Trifft zu	Trifft zu	Trifft sicher zu
Trifft zu	Trifft sicher zu	Trifft sicher zu	Trifft sicher zu
Trifft sicher zu	Trifft sicher zu	Trifft sicher zu	Trifft sicher zu
Trifft sicher zu	Trifft sicher zu	Trifft sicher zu	Trifft sicher zu
Trifft sicher zu	Trifft sicher zu	Trifft zu	Trifft sicher zu
Trifft sicher zu	Trifft sicher zu	Trifft sicher zu	Trifft sicher zu
Trifft sicher zu	Trifft sicher zu	Trifft sicher zu	Trifft sicher zu
Trifft sicher zu	Trifft sicher zu	Trifft sicher zu	Trifft sicher zu
Trifft zu	Trifft zu	Trifft zu	Trifft zu
Trifft sicher zu	Trifft sicher zu	Trifft sicher zu	Trifft sicher zu
Unentschieden	Trifft zu	Trifft zu	Trifft zu
Trifft sicher zu	Trifft sicher zu	Trifft sicher zu	Trifft sicher zu
Trifft sicher zu	Trifft sicher zu	Trifft sicher zu	Trifft sicher zu
Trifft sicher zu	Trifft sicher zu	Trifft sicher zu	Trifft sicher zu
Trifft zu	Trifft zu	Trifft zu	Trifft zu
Trifft sicher zu	Trifft sicher zu	Trifft sicher zu	Trifft sicher zu
Trifft sicher zu	Trifft sicher zu	Trifft sicher zu	Trifft sicher zu
Trifft zu	Trifft zu	Trifft zu	Trifft sicher zu
Trifft sicher zu	Trifft nicht zu	Trifft zu	Trifft sicher zu
Trifft sicher zu	Trifft sicher zu	Trifft sicher zu	Trifft sicher zu
Trifft sicher zu	Trifft nicht zu	Trifft zu	Trifft sicher zu
Trifft zu	Unentschieden	Unentschieden	Trifft sicher zu
Trifft sicher zu	Trifft sicher zu	Trifft sicher zu	Trifft sicher zu
Trifft zu	Trifft sicher zu	Trifft sicher zu	Trifft sicher zu
Trifft sicher zu	Trifft sicher zu	Trifft zu	Trifft sicher zu
Trifft sicher zu	Trifft zu	Trifft zu	Trifft sicher zu
Trifft zu	Trifft sicher zu	Trifft sicher zu	Trifft sicher zu

Trifft sicher zu	Trifft sicher zu	Trifft sicher zu	Trifft sicher zu
Trifft zu	Trifft sicher zu	Trifft sicher zu	Trifft sicher zu
Trifft zu	Trifft sicher zu	Trifft zu	Trifft zu
Trifft sicher zu	Trifft sicher zu	Unentschieden	Trifft sicher zu
Trifft zu	Trifft sicher zu	Trifft sicher zu	Trifft sicher zu
Trifft sicher zu	Trifft sicher zu	Unentschieden	Trifft sicher zu
Trifft sicher zu	Trifft sicher zu	Trifft sicher zu	Trifft sicher zu
Trifft sicher zu	Trifft sicher zu	Trifft sicher zu	Trifft sicher zu
Trifft zu	Trifft sicher zu	Trifft sicher zu	Trifft sicher zu
Trifft zu	Trifft sicher zu	Trifft zu	Trifft sicher zu
Trifft zu	Trifft sicher zu	Trifft sicher zu	Trifft sicher zu
Trifft zu	Trifft sicher zu	Trifft sicher zu	Trifft sicher zu
Trifft zu	Trifft zu	Trifft zu	Trifft zu
Unentschieden	Trifft sicher zu	Trifft zu	Trifft sicher zu
Trifft zu	Trifft zu	Trifft zu	Trifft zu
Trifft sicher zu	Trifft sicher zu	Trifft sicher zu	Trifft sicher zu
Trifft zu	Trifft sicher zu	Trifft sicher zu	Trifft sicher zu

Trifft sicher zu	Trifft sicher zu	Unentschieden	Trifft sicher zu
Unentschieden	Trifft zu	Trifft nicht zu	Unentschieden
Trifft zu	Trifft sicher zu	Unentschieden	Unentschieden
Trifft zu	Trifft sicher zu	Trifft sicher zu	Trifft sicher zu
Trifft sicher zu	Trifft sicher zu	Trifft sicher zu	Trifft sicher zu
Trifft sicher zu	Trifft sicher zu	Trifft sicher zu	Trifft sicher zu
Trifft sicher zu	Trifft sicher zu	Trifft sicher zu	Trifft sicher zu
Trifft sicher zu	Trifft sicher zu	Trifft sicher zu	Trifft sicher zu
Trifft zu	Trifft sicher zu	Trifft sicher zu	Trifft sicher zu
Unentschieden	Trifft sicher zu	Trifft zu	Trifft sicher zu
Trifft sicher zu	Trifft sicher zu	Trifft zu	Trifft sicher zu
Trifft zu	Trifft zu	Trifft sicher zu	Trifft zu
Trifft nicht zu	Trifft zu	Trifft nicht zu	Trifft zu
Unentschieden	Trifft sicher zu	Trifft zu	Trifft zu
Trifft zu	Trifft sicher zu	Trifft sicher zu	Trifft sicher zu
Trifft sicher zu	Trifft sicher zu	Trifft sicher zu	Trifft sicher zu
Trifft zu	Trifft zu	Unentschieden	Trifft zu
Trifft zu	Trifft sicher zu	Trifft zu	Trifft sicher zu
Trifft zu	Trifft sicher zu	Trifft sicher zu	Trifft sicher zu
Trifft zu	Trifft zu	Trifft zu	Trifft zu
Trifft sicher zu	Trifft sicher zu	Unentschieden	Trifft sicher zu
Trifft zu	Trifft sicher zu	Unentschieden	Trifft sicher zu
Trifft zu	Trifft sicher zu	Trifft nicht zu	Trifft sicher zu
Unentschieden	Trifft sicher zu	Trifft zu	Trifft sicher zu
Trifft zu	Trifft sicher zu	Trifft sicher zu	Trifft sicher zu
Trifft zu	Trifft zu	Trifft zu	Trifft sicher zu
Trifft sicher zu	Trifft sicher zu	Trifft sicher zu	Trifft sicher zu
Trifft sicher zu	Trifft sicher zu	Trifft sicher zu	Trifft sicher zu
Trifft zu	Trifft zu	Trifft zu	Trifft zu
Trifft zu	Trifft sicher zu	Trifft zu	Trifft sicher zu
Trifft sicher zu	Unentschieden	Trifft zu	Trifft sicher zu
Trifft zu	Trifft zu	Trifft zu	Trifft zu
Trifft sicher zu	Trifft sicher zu	Trifft sicher zu	Trifft sicher zu
Trifft zu	Trifft sicher zu	Trifft zu	Trifft sicher zu
Trifft zu	Trifft sicher zu	Trifft sicher zu	Trifft sicher zu
Unentschieden	Trifft sicher zu	Trifft sicher zu	Trifft sicher zu
Trifft sicher zu	Trifft sicher zu	Trifft sicher zu	
Trifft sicher zu	Trifft sicher zu	Trifft zu	Trifft zu
Trifft sicher zu	Trifft sicher zu	Trifft sicher zu	Trifft sicher zu
Trifft sicher zu	Trifft zu	Trifft zu	Trifft sicher zu
Trifft zu	Trifft sicher zu	Trifft sicher zu	Trifft sicher zu
Trifft sicher zu	Trifft zu	Unentschieden	Trifft zu
Trifft zu	Trifft sicher zu	Trifft sicher zu	Trifft sicher zu
Trifft sicher zu	Trifft sicher zu	Trifft zu	Trifft sicher zu
Trifft sicher zu	Trifft zu	Trifft zu	Trifft sicher zu
Trifft zu	Trifft zu	Trifft sicher zu	Trifft zu
Trifft sicher zu	Trifft sicher zu	Unentschieden	Trifft sicher zu
Trifft nicht zu	Trifft sicher zu	Trifft sicher zu	Trifft sicher zu
Trifft zu	Trifft sicher zu	Trifft zu	Trifft sicher zu
Trifft zu	Trifft zu	Unentschieden	Unentschieden

Trifft sicher zu	Trifft sicher zu	Trifft sicher zu	Trifft sicher zu
Trifft zu	Trifft zu	Unentschieden	Trifft zu
Trifft sicher zu	Trifft sicher zu	Trifft sicher zu	Unentschieden
Trifft sicher zu	Trifft sicher zu	Trifft sicher zu	Trifft sicher zu
Trifft zu	Trifft sicher zu	Trifft zu	Trifft sicher zu
Unentschieden	Trifft zu	Trifft zu	Trifft nicht zu
Trifft sicher zu	Trifft sicher zu	Trifft sicher zu	Trifft sicher zu
Trifft sicher zu	Trifft sicher zu	Trifft sicher zu	Trifft sicher zu
Trifft sicher zu	Trifft sicher zu	Trifft sicher zu	Trifft sicher zu
Unentschieden	Trifft sicher zu	Trifft sicher zu	Trifft sicher zu
Trifft sicher zu	Trifft sicher zu	Trifft sicher zu	Trifft sicher zu
Trifft sicher zu		Trifft sicher zu	Trifft sicher zu
Trifft sicher zu	Trifft sicher zu	Trifft sicher zu	Trifft sicher zu
Trifft sicher zu	Trifft sicher zu	Trifft sicher zu	Trifft sicher zu
Trifft zu	Trifft sicher zu	Trifft sicher zu	Trifft sicher zu
Trifft sicher zu	Trifft sicher zu	Trifft zu	Trifft sicher zu
Trifft zu	Trifft zu	Trifft nicht zu	Trifft zu
Trifft zu	Trifft zu	Trifft zu	Trifft zu

Das Event hat mir Spaß {	Ich habe dort viel gelern	Ich habe dort Neues gel	Ich möchte gerne klinisc
Trifft sicher zu	Trifft sicher zu	Trifft sicher zu	Trifft sicher zu
Trifft sicher zu	Trifft sicher zu	Trifft zu	Trifft sicher zu
Trifft sicher zu	Trifft sicher zu	Trifft sicher zu	Trifft sicher zu
Trifft sicher zu	Trifft sicher zu	Trifft sicher zu	Trifft sicher zu
Trifft sicher zu	Trifft sicher zu	Trifft sicher zu	Trifft zu
Trifft sicher zu	Trifft sicher zu	Trifft sicher zu	Trifft sicher zu
Trifft sicher zu	Trifft sicher zu	Trifft sicher zu	Trifft sicher zu
Trifft zu	Trifft zu	Trifft zu	Trifft sicher zu
Trifft sicher zu	Trifft zu	Unentschieden	Trifft sicher zu
Trifft sicher zu	Trifft zu	Trifft sicher zu	Trifft sicher zu
Trifft sicher zu	Trifft sicher zu	Trifft sicher zu	Trifft sicher zu
Trifft sicher zu	Trifft sicher zu	Trifft sicher zu	Trifft sicher zu
Trifft sicher zu	Trifft zu	Trifft sicher zu	Trifft sicher zu
Trifft sicher zu	Trifft sicher zu	Trifft sicher zu	Trifft sicher zu
Trifft sicher zu	Trifft sicher zu	Trifft sicher zu	Trifft sicher zu
Trifft sicher zu	Trifft sicher zu	Trifft sicher zu	Trifft sicher zu
Trifft sicher zu	Trifft sicher zu	Trifft sicher zu	Trifft sicher zu
Trifft zu	Trifft zu	Trifft zu	Trifft zu
Trifft sicher zu	Trifft sicher zu	Trifft sicher zu	Trifft sicher zu
Trifft sicher zu	Trifft zu	Trifft sicher zu	Trifft sicher zu
Trifft sicher zu	Trifft zu	Trifft sicher zu	Trifft zu
Trifft sicher zu	Trifft sicher zu	Trifft sicher zu	Trifft sicher zu
Trifft sicher zu	Trifft zu	Trifft sicher zu	Trifft sicher zu
Trifft sicher zu	Trifft sicher zu	Trifft sicher zu	Trifft sicher zu
Trifft sicher zu	Trifft sicher zu	Trifft sicher zu	Trifft sicher zu
Trifft sicher zu	Trifft zu	Trifft zu	Trifft sicher zu
Trifft sicher zu	Trifft sicher zu	Trifft zu	Trifft sicher zu
Trifft sicher zu	Trifft sicher zu	Trifft sicher zu	Trifft zu
Trifft sicher zu	Trifft sicher zu	Trifft sicher zu	Trifft sicher zu
Trifft sicher zu	Trifft sicher zu	Trifft sicher zu	Trifft sicher zu
Trifft sicher zu	Trifft sicher zu	Trifft sicher zu	Trifft sicher zu
Trifft sicher zu	Trifft zu	Trifft sicher zu	Trifft sicher zu
Trifft sicher zu	Trifft sicher zu	Trifft sicher zu	Trifft sicher zu
Trifft zu	Trifft zu	Trifft sicher zu	Trifft sicher zu
Trifft sicher zu	Trifft sicher zu	Trifft zu	Trifft zu
Trifft sicher zu	Trifft sicher zu	Trifft sicher zu	Trifft zu
Trifft sicher zu	Trifft sicher zu	Trifft sicher zu	Trifft sicher zu
Trifft sicher zu	Trifft sicher zu	Trifft sicher zu	Trifft sicher zu
Trifft sicher zu	Trifft sicher zu	Trifft sicher zu	Trifft sicher zu
Trifft sicher zu	Trifft sicher zu	Trifft zu	Trifft sicher zu
Trifft zu	Trifft zu	Trifft zu	Trifft sicher zu
Trifft sicher zu	Trifft sicher zu	Trifft sicher zu	Trifft sicher zu
Trifft sicher zu	Trifft sicher zu	Trifft sicher zu	Trifft sicher zu
Trifft zu	Trifft zu	Trifft sicher zu	Trifft sicher zu
Trifft sicher zu	Trifft sicher zu	Trifft zu	Trifft sicher zu
Trifft sicher zu	Trifft sicher zu	Trifft sicher zu	Trifft sicher zu
Unentschieden	Unentschieden	Unentschieden	Trifft sicher zu
Trifft sicher zu	Trifft zu	Trifft sicher zu	Trifft sicher zu
Trifft sicher zu	Trifft zu	Trifft sicher zu	Trifft sicher zu

Trifft zu	Trifft zu	Trifft zu	Trifft zu
Trifft sicher zu	Trifft zu	Trifft sicher zu	Trifft sicher zu
Trifft zu	Unentschieden	Unentschieden	Trifft sicher zu
Trifft sicher zu	Trifft sicher zu	Trifft sicher zu	Trifft sicher zu
Unentschieden	Unentschieden	Unentschieden	Trifft zu
Trifft zu	Trifft zu	Trifft sicher zu	Trifft sicher zu
Trifft zu	Trifft zu	Trifft zu	Trifft sicher zu
Trifft sicher zu	Trifft sicher zu	Trifft sicher zu	Trifft sicher zu
Trifft sicher zu	Trifft sicher zu	Trifft sicher zu	Trifft sicher zu
Trifft sicher zu	Trifft sicher zu	Trifft sicher zu	Trifft sicher zu
Trifft sicher zu	Trifft sicher zu	Trifft sicher zu	Trifft sicher zu
Trifft sicher zu	Trifft zu	Trifft sicher zu	Trifft sicher zu
Trifft sicher zu	Trifft zu	Trifft sicher zu	Trifft sicher zu
Trifft zu	Unentschieden	Trifft zu	Trifft zu
Trifft sicher zu	Trifft sicher zu	Trifft zu	Trifft sicher zu
Trifft zu	Trifft zu	Trifft zu	Trifft nicht zu
Trifft sicher zu	Trifft sicher zu	Trifft sicher zu	Trifft sicher zu
Trifft sicher zu	Trifft sicher zu	Trifft sicher zu	Trifft sicher zu
Trifft sicher zu	Trifft zu	Trifft sicher zu	Trifft sicher zu
Trifft sicher zu	Trifft zu	Trifft sicher zu	Trifft sicher zu
Trifft sicher zu	Trifft sicher zu	Trifft sicher zu	Trifft sicher zu
Trifft sicher zu	Trifft sicher zu	Trifft sicher zu	Trifft sicher zu
Trifft zu	Trifft sicher zu	Trifft sicher zu	Trifft sicher zu
Trifft zu	Trifft zu	Trifft sicher zu	Trifft sicher zu
Trifft sicher zu	Trifft sicher zu	Trifft sicher zu	Trifft sicher zu
Trifft sicher zu	Unentschieden	Trifft zu	Trifft sicher zu
Trifft sicher zu	Trifft zu	Trifft sicher zu	Trifft zu
Trifft sicher zu	Trifft sicher zu	Trifft sicher zu	Trifft sicher zu
Trifft sicher zu	Trifft sicher zu	Trifft sicher zu	Trifft sicher zu
Trifft sicher zu	Trifft sicher zu	Trifft sicher zu	Trifft sicher zu
Trifft sicher zu	Trifft zu	Trifft zu	Trifft sicher zu
Trifft sicher zu	Trifft sicher zu	Trifft sicher zu	Trifft sicher zu

Trifft sicher zu	Trifft sicher zu	Trifft sicher zu	Trifft sicher zu
Trifft sicher zu	Trifft sicher zu	Trifft sicher zu	Trifft sicher zu
Trifft zu			
Trifft sicher zu	Trifft sicher zu	Trifft sicher zu	Trifft sicher zu
Trifft sicher zu	Trifft sicher zu	Trifft sicher zu	Trifft sicher zu
Trifft sicher zu	Trifft zu	Unentschieden	Unentschieden
Trifft sicher zu	Trifft sicher zu	Trifft sicher zu	Trifft sicher zu
Trifft sicher zu	Trifft sicher zu	Trifft sicher zu	Trifft sicher zu
Trifft zu	Trifft zu	Trifft zu	Trifft sicher zu
Trifft sicher zu	Trifft sicher zu	Trifft sicher zu	Trifft sicher zu
Trifft sicher zu	Trifft sicher zu	Trifft sicher zu	Trifft zu
Trifft zu	Unentschieden	Trifft sicher zu	Trifft zu
Trifft zu	Unentschieden	Trifft zu	Trifft sicher zu
Unentschieden	Trifft zu	Trifft zu	Trifft sicher zu
Trifft sicher zu	Trifft zu	Trifft sicher zu	Trifft zu
Trifft sicher zu	Trifft sicher zu	Trifft sicher zu	Trifft sicher zu
Trifft sicher zu	Trifft sicher zu	Trifft sicher zu	Trifft zu

Trifft zu	Trifft sicher zu	Trifft sicher zu	Trifft sicher zu
Trifft zu	Trifft zu	Trifft zu	Unentschieden
Trifft zu	Trifft zu	Trifft zu	Unentschieden
Trifft zu	Trifft nicht zu	Trifft sicher zu	Trifft zu
Trifft sicher zu	Trifft zu	Trifft sicher zu	Trifft sicher zu
Trifft sicher zu	Trifft sicher zu	Trifft sicher zu	Trifft sicher zu
Trifft sicher zu	Trifft sicher zu	Trifft sicher zu	Trifft zu
Trifft sicher zu	Trifft sicher zu	Trifft sicher zu	Trifft sicher zu
Trifft sicher zu	Trifft zu	Trifft sicher zu	Trifft zu
Trifft sicher zu	Trifft sicher zu	Trifft sicher zu	Trifft sicher zu
Trifft sicher zu	Trifft zu	Trifft sicher zu	Trifft sicher zu
Trifft sicher zu	Trifft zu	Trifft sicher zu	Trifft zu
Trifft sicher zu	Trifft sicher zu	Trifft sicher zu	Trifft sicher zu
	Trifft sicher zu	Trifft zu	Trifft sicher zu
Trifft sicher zu	Trifft sicher zu	Trifft sicher zu	Trifft sicher zu
Trifft sicher zu	Trifft sicher zu	Trifft sicher zu	Trifft sicher zu
Trifft zu	Trifft zu	Trifft zu	Trifft zu
Trifft sicher zu	Trifft zu	Trifft sicher zu	Trifft sicher zu
Trifft sicher zu	Trifft sicher zu	Trifft sicher zu	Trifft sicher zu
Trifft zu	Trifft zu	Trifft zu	Trifft zu
Trifft sicher zu	Trifft sicher zu	Trifft sicher zu	Trifft sicher zu
Trifft sicher zu	Trifft sicher zu	Trifft zu	Trifft sicher zu
Trifft zu	Trifft sicher zu	Trifft sicher zu	Trifft sicher zu
Trifft zu	Trifft zu	Trifft sicher zu	Trifft sicher zu
Trifft sicher zu	Trifft sicher zu	Trifft sicher zu	Trifft sicher zu
Trifft sicher zu	Trifft zu	Trifft sicher zu	Trifft sicher zu
Trifft sicher zu	Trifft sicher zu	Trifft sicher zu	Trifft sicher zu
Trifft sicher zu	Trifft sicher zu	Trifft sicher zu	Trifft sicher zu
Trifft zu	Trifft zu	Trifft zu	Trifft zu
Trifft zu	Trifft zu	Trifft sicher zu	Trifft sicher zu
Trifft sicher zu	Trifft zu	Trifft sicher zu	Trifft sicher zu
Trifft zu	Trifft zu	Trifft zu	Trifft zu
Trifft sicher zu	Trifft sicher zu	Trifft sicher zu	Trifft sicher zu
Trifft sicher zu	Trifft sicher zu	Trifft sicher zu	Trifft zu
Trifft sicher zu	Trifft zu	Trifft sicher zu	Trifft sicher zu
Trifft sicher zu	Trifft sicher zu	Trifft sicher zu	Trifft sicher zu
Trifft sicher zu	Trifft sicher zu	Trifft sicher zu	Trifft sicher zu
Trifft sicher zu	Trifft sicher zu	Trifft sicher zu	Trifft sicher zu
Trifft zu	Trifft zu	Trifft zu	Trifft sicher zu
Trifft sicher zu	Trifft zu	Trifft sicher zu	Trifft sicher zu
Trifft sicher zu	Trifft zu	Trifft sicher zu	Trifft sicher zu
Trifft sicher zu	Trifft zu	Trifft zu	Trifft zu
Trifft sicher zu	Trifft zu	Trifft sicher zu	Trifft sicher zu
Trifft sicher zu	Trifft sicher zu	Trifft sicher zu	Trifft sicher zu
Trifft sicher zu	Trifft zu	Trifft sicher zu	Trifft zu
Trifft sicher zu	Trifft zu	Trifft zu	Trifft sicher zu
Trifft sicher zu	Trifft sicher zu	Trifft zu	Trifft sicher zu
Trifft sicher zu	Trifft sicher zu	Trifft sicher zu	Trifft sicher zu
Trifft sicher zu	Trifft sicher zu	Trifft zu	Trifft sicher zu
Trifft sicher zu	Trifft sicher zu	Trifft zu	Trifft sicher zu

Trifft sicher zu	Trifft sicher zu	Trifft sicher zu	Trifft sicher zu
Trifft zu	Trifft zu	Trifft zu	Trifft sicher zu
Trifft zu	Trifft zu	Trifft sicher zu	Trifft sicher zu
Trifft sicher zu	Trifft sicher zu	Trifft sicher zu	Trifft zu
Trifft sicher zu	Trifft zu	Trifft sicher zu	Trifft sicher zu
Trifft zu	Unentschieden	Unentschieden	Trifft sicher zu
Trifft sicher zu	Trifft sicher zu	Trifft sicher zu	Trifft sicher zu
Trifft sicher zu	Trifft sicher zu	Trifft sicher zu	Trifft sicher zu
Trifft sicher zu	Trifft sicher zu	Trifft sicher zu	Trifft zu
Trifft sicher zu	Trifft sicher zu	Trifft sicher zu	Trifft sicher zu
Trifft sicher zu	Trifft sicher zu	Trifft sicher zu	Trifft sicher zu
Trifft sicher zu	Trifft sicher zu	Trifft sicher zu	Trifft sicher zu
Trifft sicher zu	Trifft sicher zu	Trifft sicher zu	Trifft sicher zu
Trifft sicher zu	Trifft sicher zu	Trifft sicher zu	Trifft sicher zu
Trifft sicher zu	Trifft sicher zu	Trifft sicher zu	Trifft sicher zu
Trifft sicher zu	Trifft sicher zu	Trifft sicher zu	Trifft sicher zu
Trifft sicher zu	Trifft sicher zu	Trifft zu	Trifft sicher zu
Trifft sicher zu	Trifft sicher zu	Trifft sicher zu	Trifft sicher zu

Differentialdiagnostisch	Der Durchführung einer	Der Beurteilung von Lab	Der Beurteilung einer Bi
	Etwas	Ab und zu	Häufig
	Ab und zu	Ab und zu	Ab und zu
	Etwas	Etwas	Ab und zu
	Etwas	Etwas	Etwas
	Etwas	Etwas	Gar keine
	Etwas	Etwas	Etwas
	Ab und zu	Etwas	Etwas
	Ab und zu	Ab und zu	Etwas
	Ab und zu	Etwas	Etwas
	Ab und zu	Etwas	Gar keine
	Ab und zu	Häufig	Ab und zu
	Sehr viel	Ab und zu	Etwas
	Etwas	Etwas	Etwas
	Ab und zu	Ab und zu	Ab und zu
	Etwas	Etwas	Etwas
	Etwas	Etwas	Etwas
	Etwas	Etwas	Ab und zu
	Etwas	Ab und zu	Ab und zu
	Etwas	Ab und zu	Etwas
	Häufig	Häufig	Ab und zu
	Ab und zu	Etwas	Ab und zu
	Etwas	Häufig	Etwas
	Gar keine	Etwas	Gar keine
	Etwas	Ab und zu	Gar keine
	Etwas	Etwas	Etwas
	Gar keine	Etwas	Ab und zu
	Gar keine	Gar keine	Etwas
	Etwas	Etwas	Etwas
	Etwas	Etwas	Etwas
	Ab und zu	Etwas	Etwas
	Etwas	Häufig	Ab und zu
	Etwas	Etwas	Gar keine
	Ab und zu	Ab und zu	Etwas
	Ab und zu	Ab und zu	Ab und zu
	Gar keine	Etwas	Ab und zu
	Etwas	Etwas	Etwas
	Gar keine	Etwas	Etwas
	Etwas	Etwas	Etwas
	Etwas	Etwas	Etwas
	Ab und zu	Etwas	Etwas
	Ab und zu	Ab und zu	Etwas
	Etwas	Etwas	Gar keine
	Ab und zu	Ab und zu	Etwas
	Ab und zu	Ab und zu	Etwas
	Gar keine	Gar keine	Gar keine
	Häufig	Ab und zu	Etwas
Trifft sicher zu	Häufig	Ab und zu	Häufig
Trifft zu	Etwas	Gar keine	Etwas
Trifft sicher zu	Etwas	Etwas	Etwas

Trifft zu	Ab und zu	Ab und zu	Ab und zu
Trifft sicher zu	Etwas	Etwas	Etwas
Trifft sicher zu	Ab und zu	Ab und zu	Etwas
Trifft sicher zu	Häufig	Häufig	Ab und zu
Trifft sicher zu	Gar keine	Etwas	Ab und zu
Trifft sicher zu	Etwas	Etwas	Etwas
Trifft sicher zu	Etwas	Gar keine	Etwas
Trifft sicher zu	Etwas	Etwas	Etwas
Trifft sicher zu	Ab und zu	Ab und zu	Etwas
Trifft sicher zu	Sehr viel	Ab und zu	Ab und zu
Trifft sicher zu	Etwas	Etwas	Etwas
Trifft sicher zu	Ab und zu	Ab und zu	Ab und zu
Trifft zu	Ab und zu	Ab und zu	Ab und zu
Trifft sicher zu	Häufig	Ab und zu	Ab und zu
Trifft zu	Etwas	Etwas	Gar keine
Trifft sicher zu	Ab und zu	Ab und zu	Ab und zu
Trifft sicher zu	Etwas	Etwas	Etwas
Trifft sicher zu	Gar keine	Gar keine	Gar keine
Trifft zu	Ab und zu	Ab und zu	Ab und zu
Trifft sicher zu	Etwas	Etwas	Etwas
Trifft sicher zu	Ab und zu	Häufig	Ab und zu
Trifft sicher zu	Ab und zu	Etwas	Etwas
Trifft sicher zu	Gar keine	Etwas	Etwas
Trifft sicher zu	Etwas	Etwas	Etwas
Trifft sicher zu	Gar keine	Gar keine	Gar keine
Trifft zu	Ab und zu	Ab und zu	Etwas
Trifft sicher zu	Gar keine	Gar keine	Gar keine
Trifft sicher zu	Etwas	Etwas	Etwas
Trifft sicher zu	Gar keine	Gar keine	Gar keine
Trifft zu	Ab und zu	Etwas	Etwas
Trifft sicher zu	Gar keine	Etwas	Etwas
	Etwas	Etwas	Ab und zu
Trifft sicher zu	Etwas	Etwas	Etwas
Trifft sicher zu	Etwas	Etwas	Etwas
	Häufig	Häufig	Ab und zu
Trifft sicher zu	Etwas	Etwas	Gar keine
Trifft sicher zu	Etwas	Ab und zu	Gar keine
Trifft sicher zu	Ab und zu	Etwas	Etwas
Trifft sicher zu	Gar keine	Etwas	Gar keine
Trifft sicher zu	Etwas	Ab und zu	Etwas
Trifft sicher zu	Ab und zu	Ab und zu	Etwas
Trifft sicher zu	Etwas	Ab und zu	Ab und zu
Trifft sicher zu	Ab und zu	Gar keine	Gar keine
Trifft sicher zu	Ab und zu	Ab und zu	Etwas
Trifft sicher zu	Ab und zu	Ab und zu	Etwas
Unentschieden	Ab und zu	Etwas	Etwas
Trifft sicher zu	Häufig	Ab und zu	Ab und zu
Trifft sicher zu	Etwas	Etwas	Ab und zu
Trifft sicher zu	Ab und zu	Ab und zu	Etwas

Trifft sicher zu	Gar keine	Etwas	Gar keine
Trifft zu	Etwas	Etwas	Etwas
Unentschieden	Ab und zu	Ab und zu	Etwas
Trifft zu	Etwas	Etwas	Gar keine
Trifft sicher zu	Etwas	Etwas	Etwas
Trifft sicher zu	Häufig	Häufig	Etwas
Trifft sicher zu	Ab und zu	Ab und zu	Etwas
Trifft sicher zu	Gar keine	Etwas	Gar keine
Trifft sicher zu	Häufig	Häufig	Häufig
Trifft sicher zu	Ab und zu	Ab und zu	Sehr viel
Trifft sicher zu	Etwas	Etwas	Etwas
Trifft zu	Etwas	Etwas	Etwas
Trifft sicher zu	Ab und zu	Ab und zu	Etwas
Trifft sicher zu	Häufig	Häufig	Ab und zu
Trifft sicher zu	Häufig	Häufig	Ab und zu
Trifft sicher zu	Ab und zu	Etwas	Ab und zu
Unentschieden	Etwas	Etwas	Etwas
Trifft sicher zu	Etwas	Etwas	Etwas
Trifft sicher zu	Häufig	Ab und zu	Häufig
Trifft zu	Etwas	Etwas	Etwas
Trifft zu	Etwas	Etwas	Etwas
Trifft sicher zu	Häufig	Ab und zu	Etwas
Trifft sicher zu	Sehr viel	Häufig	Ab und zu
Trifft zu	Ab und zu	Ab und zu	Etwas
Trifft sicher zu	Häufig	Ab und zu	Etwas
Trifft zu	Gar keine	Etwas	Gar keine
Trifft sicher zu	Ab und zu	Gar keine	Etwas
Trifft sicher zu	Häufig	Ab und zu	Ab und zu
Trifft zu	Gar keine	Etwas	Gar keine
Trifft zu	Ab und zu	Etwas	Etwas
Trifft sicher zu	Gar keine	Etwas	Etwas
Trifft zu	Ab und zu	Ab und zu	Etwas
Trifft sicher zu	Etwas	Gar keine	Etwas
Trifft sicher zu	Gar keine	Ab und zu	Etwas
Trifft sicher zu	Etwas	Etwas	Etwas
Trifft sicher zu	Etwas	Etwas	Etwas
Trifft sicher zu	Ab und zu	Etwas	Sehr viel
Trifft sicher zu	Häufig	Häufig	Häufig
Trifft sicher zu	Etwas	Ab und zu	Etwas
Trifft sicher zu	Etwas	Ab und zu	Etwas
Trifft zu	Etwas	Etwas	Etwas
Trifft sicher zu	Ab und zu	Ab und zu	Etwas
Trifft sicher zu	Etwas	Gar keine	Gar keine
Trifft sicher zu	Etwas	Etwas	Gar keine
Trifft zu	Etwas	Etwas	Etwas
Trifft zu	Ab und zu	Ab und zu	Häufig
Trifft sicher zu	Ab und zu	Häufig	Ab und zu
Trifft sicher zu	Häufig	Ab und zu	Häufig
Trifft sicher zu	Häufig	Ab und zu	Ab und zu
Trifft sicher zu	Sehr viel	Häufig	Häufig

Trifft sicher zu	Häufig	Häufig	Häufig
Trifft zu	Ab und zu	Ab und zu	Ab und zu
Unentschieden	Ab und zu	Etwas	Ab und zu
Trifft sicher zu	Ab und zu	Ab und zu	Etwas
Trifft sicher zu	Etwas	Gar keine	Etwas
Trifft sicher zu	Sehr viel	Häufig	Ab und zu
Trifft sicher zu	Etwas	Etwas	Etwas
Trifft sicher zu	Ab und zu	Ab und zu	Etwas
Trifft sicher zu	Gar keine	Etwas	Gar keine
Trifft sicher zu	Ab und zu	Etwas	Häufig
Trifft zu	Häufig	Ab und zu	Ab und zu
Trifft sicher zu	Ab und zu	Etwas	Etwas
Trifft sicher zu	Ab und zu	Ab und zu	Etwas
Trifft zu	Etwas	Etwas	Etwas
Trifft sicher zu	Ab und zu	Ab und zu	Etwas
Trifft zu	Gar keine	Etwas	Etwas
Trifft sicher zu	Häufig	Ab und zu	Häufig
Trifft sicher zu	Etwas	Ab und zu	Etwas

Dem Lösen sogenannter	Der Beurteilung, welche	Der Beurteilung von bed	Der Anspruch der Veran:
Ab und zu	Häufig	Etwas	Trifft sicher zu
Etwas	Ab und zu	Ab und zu	Trifft zu
Gar keine	Etwas	Etwas	Trifft sicher zu
Ab und zu	Ab und zu	Ab und zu	Trifft sicher zu
Etwas	Ab und zu	Etwas	Trifft sicher zu
Etwas	Etwas	Etwas	Trifft sicher zu
Etwas	Etwas	Etwas	Trifft sicher zu
Ab und zu	Häufig	Ab und zu	Trifft zu
Gar keine	Etwas	Etwas	Trifft zu
Etwas	Etwas	Gar keine	Trifft zu
Ab und zu	Etwas	Etwas	Trifft sicher zu
Gar keine	Gar keine	Ab und zu	Trifft sicher zu
Ab und zu	Etwas	Ab und zu	Trifft sicher zu
Etwas	Ab und zu	Ab und zu	Trifft sicher zu
Etwas	Etwas	Etwas	Trifft sicher zu
Etwas	Etwas	Etwas	Trifft sicher zu
Etwas	Etwas	Gar keine	Trifft sicher zu
Ab und zu	Etwas	Etwas	Trifft zu
Ab und zu	Etwas	Etwas	Trifft sicher zu
Ab und zu	Etwas	Etwas	Trifft zu
Etwas	Etwas	Häufig	Trifft zu
Gar keine	Ab und zu	Ab und zu	Trifft sicher zu
Gar keine	Gar keine	Gar keine	Trifft zu
Etwas	Gar keine	Gar keine	Trifft sicher zu
Gar keine	Gar keine	Gar keine	Trifft sicher zu
Gar keine	Gar keine	Etwas	Trifft sicher zu
Etwas	Gar keine	Gar keine	Trifft zu
Etwas	Gar keine	Gar keine	Trifft sicher zu
Etwas	Etwas	Etwas	Trifft sicher zu
Ab und zu	Ab und zu	Etwas	Trifft sicher zu
Etwas	Gar keine	Häufig	Trifft sicher zu
Etwas	Gar keine	Etwas	Trifft zu
Etwas	Etwas	Gar keine	Trifft sicher zu
Etwas	Etwas	Gar keine	Trifft sicher zu
Etwas			Trifft sicher zu
Etwas	Etwas	Etwas	Trifft sicher zu
Etwas	Etwas	Etwas	Trifft sicher zu
Etwas	Gar keine	Etwas	Trifft sicher zu
Etwas	Etwas	Etwas	Trifft sicher zu
Etwas	Ab und zu	Ab und zu	Trifft sicher zu
Etwas	Etwas	Etwas	Trifft zu
Gar keine	Etwas	Gar keine	Trifft sicher zu
Ab und zu	Etwas	Gar keine	Trifft sicher zu
Gar keine	Etwas	Etwas	Trifft zu
Gar keine	Gar keine	Gar keine	Trifft sicher zu
Ab und zu	Ab und zu	Ab und zu	Trifft sicher zu
Etwas	Ab und zu	Ab und zu	Trifft zu
Gar keine	Gar keine	Etwas	Trifft zu
Etwas	Etwas	Gar keine	Trifft sicher zu

Ab und zu	Etwas	Häufig	Trifft zu
Gar keine	Ab und zu	Etwas	Trifft zu
Ab und zu	Ab und zu	Ab und zu	Trifft zu
Ab und zu	Etwas	Etwas	Trifft sicher zu
	Etwas	Etwas	Trifft sicher zu
Etwas	Gar keine	Gar keine	Trifft sicher zu
Etwas	Gar keine	Gar keine	Unentschieden
Gar keine	Etwas	Etwas	Trifft sicher zu
			Trifft sicher zu
Etwas	Etwas	Gar keine	Trifft sicher zu
Sehr viel	Etwas	Gar keine	Trifft sicher zu
Etwas	Etwas	Etwas	Trifft zu
Gar keine	Ab und zu	Gar keine	Trifft zu
Ab und zu	Ab und zu	Ab und zu	Trifft zu
Etwas	Ab und zu	Gar keine	Trifft sicher zu
Gar keine	Gar keine	Etwas	Trifft zu
Etwas	Ab und zu	Etwas	Trifft sicher zu
Etwas	Etwas	Etwas	Trifft sicher zu
Gar keine	Gar keine	Gar keine	Trifft zu
Etwas	Ab und zu	Etwas	Trifft sicher zu
Gar keine	Gar keine	Gar keine	Trifft sicher zu
Gar keine	Etwas	Ab und zu	Trifft sicher zu
Gar keine	Etwas	Etwas	Trifft sicher zu
Gar keine	Etwas	Gar keine	Trifft zu
Gar keine	Etwas	Sehr viel	Trifft sicher zu
Gar keine	Gar keine	Gar keine	Trifft sicher zu
Gar keine	Gar keine	Häufig	Unentschieden
Gar keine	Gar keine	Gar keine	Trifft sicher zu
Häufig	Etwas	Gar keine	Trifft sicher zu
Gar keine	Gar keine	Gar keine	Trifft sicher zu
Ab und zu	Etwas	Gar keine	Trifft zu
Gar keine	Gar keine	Etwas	Trifft sicher zu
Etwas	Gar keine	Etwas	Trifft sicher zu
Etwas	Etwas	Etwas	Trifft zu
Etwas	Etwas	Ab und zu	Trifft sicher zu
Etwas	Gar keine	Gar keine	Trifft zu
Etwas	Gar keine	Ab und zu	Trifft zu
Gar keine	Gar keine	Ab und zu	Trifft sicher zu
Ab und zu	Etwas	Ab und zu	Trifft sicher zu
Gar keine	Gar keine	Etwas	Trifft sicher zu
Etwas	Etwas	Etwas	Trifft sicher zu
Etwas	Ab und zu	Gar keine	Trifft zu
Ab und zu	Ab und zu	Häufig	Trifft zu
Etwas	Etwas	Ab und zu	Trifft zu
Etwas	Häufig	Häufig	Unentschieden
Ab und zu	Ab und zu	Sehr viel	Trifft sicher zu
Etwas	Ab und zu	Etwas	Trifft zu
Häufig	Ab und zu	Sehr viel	Trifft sicher zu
Gar keine	Ab und zu	Ab und zu	Trifft sicher zu
Häufig	Etwas	Gar keine	Trifft sicher zu

Etwas	Etwas	Etwas	Trifft zu
Gar keine	Ab und zu	Gar keine	Unentschieden
Gar keine	Etwas	Gar keine	Trifft zu
Gar keine	Etwas	Etwas	Unentschieden
Gar keine	Gar keine	Etwas	Trifft zu
Etwas	Ab und zu	Ab und zu	Trifft sicher zu
Ab und zu	Etwas	Etwas	Trifft zu
Etwas	Etwas	Etwas	Trifft sicher zu
Etwas	Ab und zu	Ab und zu	Trifft zu
Etwas	Ab und zu	Etwas	Trifft sicher zu
Etwas	Etwas	Etwas	Trifft sicher zu
Ab und zu	Gar keine	Etwas	Trifft zu
Etwas	Etwas	Gar keine	Trifft sicher zu
Ab und zu	Ab und zu	Ab und zu	Trifft sicher zu
Ab und zu	Etwas	Etwas	Trifft sicher zu
Etwas	Häufig	Ab und zu	Trifft sicher zu
Gar keine	Gar keine	Gar keine	Trifft zu
Etwas	Etwas	Ab und zu	Trifft zu
Ab und zu	Ab und zu	Ab und zu	Trifft sicher zu
Etwas	Etwas	Etwas	Trifft zu
Gar keine	Etwas	Etwas	Trifft sicher zu
Etwas	Ab und zu	Ab und zu	Trifft zu
Etwas	Ab und zu	Etwas	Unentschieden
Etwas	Etwas	Etwas	Trifft sicher zu
Etwas	Ab und zu	Etwas	Trifft zu
Etwas	Etwas	Etwas	Trifft zu
Etwas	Etwas	Häufig	Trifft sicher zu
Ab und zu	Häufig	Ab und zu	Trifft sicher zu
Gar keine	Gar keine	Etwas	Trifft zu
Gar keine	Ab und zu	Etwas	Trifft sicher zu
Gar keine	Etwas	Gar keine	Trifft sicher zu
Etwas	Ab und zu	Gar keine	Trifft zu
Etwas	Etwas	Etwas	Trifft sicher zu
Gar keine	Etwas	Ab und zu	Trifft zu
Etwas	Etwas	Ab und zu	Trifft zu
Etwas	Ab und zu	Ab und zu	Trifft sicher zu
Häufig	Ab und zu	Etwas	Trifft sicher zu
Ab und zu	Häufig	Ab und zu	Trifft sicher zu
Gar keine	Gar keine	Etwas	Trifft sicher zu
Etwas	Etwas	Gar keine	Trifft zu
Gar keine	Etwas	Gar keine	Trifft sicher zu
Ab und zu	Etwas	Ab und zu	Trifft sicher zu
Gar keine	Gar keine	Gar keine	Trifft zu
Etwas	Etwas	Etwas	Trifft sicher zu
Gar keine	Gar keine	Gar keine	Trifft zu
Ab und zu	Ab und zu	Ab und zu	Trifft zu
Ab und zu	Etwas	Etwas	Trifft zu
Etwas	Ab und zu	Ab und zu	Trifft sicher zu
Etwas	Ab und zu	Etwas	Trifft sicher zu
Ab und zu	Ab und zu	Häufig	Trifft zu

Etwas	Ab und zu	Häufig	Trifft sicher zu
Ab und zu	Ab und zu	Häufig	Trifft zu
Ab und zu	Ab und zu	Etwas	Trifft zu
Etwas	Etwas	Gar keine	Trifft sicher zu
Gar keine	Gar keine	Gar keine	Trifft sicher zu
Etwas	Häufig	Ab und zu	Trifft zu
Etwas	Ab und zu	Gar keine	Trifft sicher zu
Etwas	Etwas	Etwas	Unentschieden
Gar keine	Gar keine	Gar keine	Trifft sicher zu
Sehr viel	Gar keine	Gar keine	Trifft sicher zu
Etwas	Etwas	Etwas	Trifft sicher zu
Ab und zu	Häufig	Etwas	Trifft sicher zu
Ab und zu	Ab und zu	Ab und zu	Trifft sicher zu
Etwas	Etwas	Etwas	Trifft sicher zu
Ab und zu	Ab und zu	Häufig	Trifft zu
Gar keine	Gar keine	Gar keine	Trifft sicher zu
Etwas	Häufig	Häufig	Trifft sicher zu
Etwas	Etwas	Etwas	Trifft sicher zu

Der Besuch der Lehrvera	Die Auswahl und Lösbar	Durch die Veranstaltung	Das Erlernen von Blickdi
Trifft sicher zu	Trifft sicher zu	Trifft sicher zu	Trifft sicher zu
Trifft zu	Trifft zu	Trifft zu	Trifft zu
Trifft sicher zu	Trifft sicher zu	Trifft sicher zu	Trifft sicher zu
Trifft sicher zu	Trifft sicher zu	Trifft sicher zu	Trifft sicher zu
Trifft sicher zu	Trifft sicher zu	Trifft sicher zu	Trifft sicher zu
Trifft sicher zu	Trifft sicher zu	Trifft sicher zu	Trifft sicher zu
Trifft sicher zu	Trifft sicher zu	Trifft sicher zu	Trifft sicher zu
Trifft zu	Trifft zu	Trifft zu	Trifft sicher zu
Unentschieden	Trifft zu	Unentschieden	Trifft sicher zu
Trifft sicher zu	Trifft zu	Trifft zu	Trifft sicher zu
Trifft sicher zu	Trifft sicher zu	Trifft sicher zu	Trifft sicher zu
Trifft sicher zu	Trifft zu	Trifft sicher zu	Trifft sicher zu
Trifft sicher zu	Trifft sicher zu	Trifft zu	Trifft zu
Trifft sicher zu	Trifft zu	Trifft zu	Trifft sicher zu
Trifft sicher zu	Trifft sicher zu	Trifft sicher zu	Trifft sicher zu
Trifft sicher zu	Trifft sicher zu	Trifft sicher zu	Trifft sicher zu
Trifft sicher zu	Trifft sicher zu	Trifft zu	Trifft sicher zu
Unentschieden	Trifft zu	Unentschieden	Trifft zu
Trifft sicher zu	Trifft sicher zu	Trifft sicher zu	Trifft sicher zu
Trifft sicher zu	Trifft sicher zu	Unentschieden	Trifft sicher zu
Unentschieden	Trifft zu	Trifft nicht zu	Trifft sicher zu
Trifft sicher zu	Trifft sicher zu	Trifft sicher zu	Trifft sicher zu
Trifft sicher zu	Trifft zu	Trifft sicher zu	Trifft sicher zu
Trifft sicher zu	Trifft sicher zu	Trifft sicher zu	Trifft sicher zu
Trifft zu	Trifft zu	Trifft zu	Trifft sicher zu
Trifft zu	Trifft sicher zu	Unentschieden	Trifft sicher zu
Trifft zu	Trifft sicher zu	Trifft zu	Trifft sicher zu
Trifft zu	Trifft zu	Trifft zu	Trifft sicher zu
Trifft sicher zu	Trifft sicher zu	Trifft sicher zu	Trifft sicher zu
Trifft sicher zu	Trifft zu	Trifft zu	Trifft sicher zu
Trifft sicher zu	Trifft sicher zu	Trifft sicher zu	Trifft sicher zu
Trifft zu	Trifft zu	Trifft zu	Trifft zu
Trifft sicher zu	Trifft sicher zu	Trifft sicher zu	Trifft sicher zu
Trifft zu	Unentschieden	Unentschieden	Trifft sicher zu
Trifft sicher zu	Trifft zu	Unentschieden	Trifft sicher zu
Trifft sicher zu	Trifft sicher zu	Unentschieden	Trifft zu
Trifft sicher zu	Trifft sicher zu	Trifft sicher zu	Trifft sicher zu
Trifft sicher zu	Trifft sicher zu	Trifft sicher zu	Trifft sicher zu
Trifft sicher zu	Trifft sicher zu	Trifft zu	Trifft sicher zu
Trifft zu	Trifft zu	Trifft zu	Trifft sicher zu
Trifft sicher zu	Trifft sicher zu	Trifft sicher zu	Trifft sicher zu
Trifft sicher zu	Trifft sicher zu	Trifft sicher zu	Trifft sicher zu
Trifft sicher zu	Trifft sicher zu	Trifft zu	Trifft sicher zu
Trifft sicher zu	Trifft sicher zu	Trifft sicher zu	Trifft sicher zu
Trifft sicher zu	Trifft sicher zu	Trifft sicher zu	Trifft sicher zu
Trifft zu	Trifft zu	Trifft zu	Trifft sicher zu
Trifft zu	Trifft zu	Unentschieden	Trifft zu
Trifft zu	Trifft zu	Trifft zu	Trifft sicher zu

Trifft zu	Trifft zu	Unentschieden	Trifft zu
Trifft zu	Trifft zu	Unentschieden	Trifft sicher zu
Trifft zu	Trifft zu	Trifft zu	Trifft zu
Trifft sicher zu	Trifft sicher zu	Trifft sicher zu	Trifft sicher zu
Trifft sicher zu	Trifft sicher zu	Trifft sicher zu	Trifft sicher zu
Trifft zu	Trifft zu	Trifft zu	Trifft sicher zu
Trifft zu	Trifft zu	Trifft zu	Trifft sicher zu
Trifft sicher zu	Trifft sicher zu	Trifft sicher zu	Trifft sicher zu
Trifft sicher zu	Trifft zu	Trifft zu	Trifft sicher zu
Trifft sicher zu	Trifft sicher zu	Trifft sicher zu	Trifft sicher zu
Trifft sicher zu	Trifft sicher zu	Trifft sicher zu	Trifft sicher zu
Trifft zu	Trifft zu	Unentschieden	Trifft sicher zu
Trifft zu	Trifft zu	Trifft zu	Trifft sicher zu
Trifft zu	Trifft zu	Trifft zu	Trifft zu
Trifft sicher zu	Trifft zu	Trifft sicher zu	Trifft sicher zu
Trifft zu	Trifft zu	Trifft zu	Trifft zu
Trifft sicher zu	Trifft zu	Trifft sicher zu	Trifft sicher zu
Trifft sicher zu	Trifft sicher zu	Trifft sicher zu	Trifft sicher zu
Trifft sicher zu	Trifft zu	Trifft zu	Trifft sicher zu
Trifft sicher zu	Trifft zu	Trifft zu	Trifft sicher zu
Trifft sicher zu	Unentschieden	Trifft sicher zu	Trifft sicher zu
Trifft sicher zu	Trifft sicher zu	Trifft sicher zu	Trifft sicher zu
Trifft sicher zu	Trifft sicher zu	Trifft zu	Trifft sicher zu
Trifft zu	Trifft nicht zu	Trifft nicht zu	Trifft sicher zu
Trifft sicher zu	Trifft zu	Trifft zu	Trifft sicher zu
Trifft zu	Trifft sicher zu	Trifft nicht zu	Trifft zu
Trifft zu	Unentschieden	Unentschieden	Trifft sicher zu
Trifft sicher zu	Unentschieden	Trifft zu	Trifft sicher zu
Trifft sicher zu	Trifft sicher zu	Trifft zu	Trifft zu
Trifft zu	Trifft zu	Trifft zu	Trifft sicher zu
Trifft zu	Trifft zu	Trifft zu	Trifft sicher zu
Trifft sicher zu	Trifft sicher zu	Trifft sicher zu	Trifft sicher zu
Trifft sicher zu	Trifft zu	Trifft zu	Trifft sicher zu
Trifft sicher zu	Unentschieden	Trifft zu	Trifft sicher zu
Trifft sicher zu	Trifft sicher zu	Trifft zu	Trifft zu
Trifft zu	Trifft zu	Unentschieden	Trifft zu
Trifft sicher zu	Unentschieden	Trifft zu	Trifft sicher zu
Trifft sicher zu	Trifft sicher zu	Trifft sicher zu	Trifft sicher zu
Trifft zu	Trifft zu	Trifft zu	Trifft zu
Trifft sicher zu	Trifft zu	Trifft sicher zu	Trifft sicher zu
Trifft sicher zu	Trifft sicher zu	Trifft sicher zu	Trifft sicher zu
Trifft zu	Trifft zu	Trifft zu	Trifft zu
Trifft zu	Trifft zu	Trifft zu	Trifft sicher zu
Unentschieden	Trifft sicher zu	Unentschieden	Trifft sicher zu
Trifft zu	Unentschieden	Trifft zu	Trifft sicher zu
Trifft zu	Trifft zu	Unentschieden	Trifft sicher zu
Trifft zu	Trifft zu	Trifft zu	Trifft sicher zu
Trifft zu	Trifft zu	Trifft zu	Trifft zu
Trifft sicher zu	Trifft sicher zu	Trifft sicher zu	Trifft sicher zu
Trifft sicher zu	Trifft sicher zu	Trifft sicher zu	Trifft sicher zu

Trifft zu	Trifft zu	Trifft zu	Trifft zu
Trifft zu	Trifft zu	Unentschieden	Trifft zu
Trifft zu	Trifft zu	Unentschieden	Unentschieden
Trifft zu	Trifft nicht zu	Trifft zu	Trifft sicher zu
Trifft zu	Trifft sicher zu	Trifft zu	Trifft sicher zu
Trifft sicher zu	Trifft sicher zu	Trifft sicher zu	Trifft sicher zu
Trifft sicher zu	Trifft sicher zu	Trifft sicher zu	Trifft sicher zu
Trifft sicher zu	Trifft sicher zu	Trifft sicher zu	Trifft sicher zu
Trifft sicher zu	Trifft zu	Trifft zu	Trifft zu
Trifft sicher zu	Trifft zu	Trifft sicher zu	Trifft sicher zu
Trifft zu	Trifft sicher zu	Trifft sicher zu	Trifft sicher zu
Trifft zu	Trifft zu	Trifft zu	Trifft sicher zu
Trifft zu	Trifft zu	Trifft zu	Trifft sicher zu
Trifft sicher zu	Trifft sicher zu	Trifft zu	Trifft sicher zu
Trifft sicher zu	Trifft sicher zu	Trifft sicher zu	Trifft sicher zu
Trifft sicher zu	Trifft zu	Trifft sicher zu	Trifft sicher zu
Trifft zu	Trifft zu	Unentschieden	Trifft sicher zu
Trifft zu	Trifft zu	Trifft zu	Trifft sicher zu
Trifft sicher zu	Trifft zu	Trifft sicher zu	Trifft sicher zu
Trifft zu	Unentschieden	Trifft zu	Trifft zu
Trifft sicher zu	Trifft zu	Trifft sicher zu	Trifft zu
Trifft sicher zu	Trifft zu	Trifft zu	Trifft zu
Trifft zu	Trifft nicht zu	Trifft zu	Trifft zu
Trifft sicher zu	Trifft zu	Trifft zu	Trifft sicher zu
Trifft sicher zu	Trifft zu	Trifft sicher zu	Trifft zu
Trifft sicher zu	Trifft zu	Trifft zu	Trifft zu
Trifft sicher zu	Trifft sicher zu	Trifft sicher zu	Trifft sicher zu
Trifft sicher zu	Trifft zu	Trifft zu	Trifft sicher zu
Trifft zu	Trifft zu	Trifft zu	Trifft sicher zu
Trifft zu	Trifft zu	Unentschieden	Trifft sicher zu
Trifft zu	Trifft sicher zu	Unentschieden	Trifft sicher zu
Trifft zu	Trifft zu	Trifft zu	Trifft zu
Trifft sicher zu	Trifft zu	Trifft sicher zu	Trifft sicher zu
Trifft zu	Trifft zu	Trifft sicher zu	Trifft zu
Trifft sicher zu	Trifft zu	Trifft zu	Trifft sicher zu
Trifft zu	Trifft zu	Trifft zu	Trifft sicher zu
Trifft sicher zu	Trifft sicher zu	Trifft sicher zu	Trifft sicher zu
Trifft sicher zu	Trifft zu	Trifft sicher zu	Trifft sicher zu
Trifft sicher zu	Trifft zu	Trifft zu	Trifft sicher zu
Trifft zu	Trifft zu	Unentschieden	Trifft sicher zu
Trifft sicher zu	Trifft zu	Trifft zu	Trifft sicher zu
Trifft zu	Trifft sicher zu	Trifft zu	Trifft sicher zu
Unentschieden	Trifft sicher zu	Unentschieden	Trifft sicher zu
Trifft sicher zu	Trifft sicher zu	Trifft zu	Trifft sicher zu
Trifft sicher zu	Trifft zu	Unentschieden	Trifft zu
Trifft zu	Trifft zu	Trifft zu	Trifft sicher zu
Trifft sicher zu	Trifft sicher zu	Trifft sicher zu	Trifft sicher zu
Trifft sicher zu	Trifft zu	Trifft sicher zu	Trifft sicher zu
Trifft sicher zu	Trifft sicher zu	Trifft zu	Trifft sicher zu
Trifft sicher zu	Trifft zu	Trifft zu	Trifft sicher zu

Trifft sicher zu	Trifft zu	Trifft zu	Trifft zu
Trifft zu	Trifft zu	Trifft zu	Trifft sicher zu
Trifft zu	Trifft zu	Trifft zu	Trifft sicher zu
Trifft sicher zu	Trifft sicher zu	Trifft sicher zu	Trifft zu
Trifft zu	Trifft zu	Trifft zu	Trifft sicher zu
Trifft sicher zu	Trifft sicher zu	Trifft sicher zu	Trifft sicher zu
Trifft sicher zu	Trifft sicher zu	Trifft sicher zu	Trifft sicher zu
Trifft zu	Unentschieden	Trifft zu	Trifft zu
Trifft sicher zu	Trifft zu	Trifft zu	Trifft sicher zu
Trifft sicher zu	Trifft sicher zu	Trifft sicher zu	Trifft sicher zu
Trifft sicher zu	Trifft zu	Trifft zu	Trifft sicher zu
Trifft sicher zu	Trifft sicher zu	Trifft zu	Trifft sicher zu
Trifft sicher zu	Trifft sicher zu	Trifft sicher zu	Trifft sicher zu
Trifft sicher zu	Trifft zu	Unentschieden	Trifft zu
Trifft sicher zu	Trifft zu	Trifft sicher zu	Unentschieden
Trifft zu	Trifft zu	Trifft zu	Trifft sicher zu
Trifft sicher zu	Trifft sicher zu	Trifft sicher zu	Trifft sicher zu
Trifft sicher zu	Trifft sicher zu	Trifft zu	Trifft sicher zu

Die Lösbarkeit der Blick	Ich würde die Veranstalt	Die Veranstaltung hat fü	Der zeitliche Umfang de
Trifft sicher zu	Trifft zu	Trifft sicher nicht zu	Trifft sicher zu
Trifft zu	Trifft sicher zu	Trifft nicht zu	Trifft zu
Trifft sicher zu	Trifft sicher zu	Trifft sicher nicht zu	Trifft sicher zu
Trifft zu	Trifft sicher zu	Trifft sicher nicht zu	Trifft sicher zu
Trifft sicher zu	Trifft sicher zu	Trifft sicher nicht zu	Trifft sicher zu
Trifft sicher zu	Trifft sicher zu	Trifft sicher nicht zu	Trifft zu
Trifft sicher zu	Trifft sicher zu	Trifft sicher nicht zu	Trifft zu
Trifft sicher zu	Trifft sicher zu	Trifft nicht zu	Trifft sicher zu
Unentschieden	Trifft zu	Trifft sicher nicht zu	Unentschieden
Trifft zu	Trifft sicher zu	Trifft nicht zu	Trifft zu
Trifft sicher zu	Trifft sicher zu	Trifft sicher nicht zu	Trifft sicher zu
Trifft zu	Trifft sicher zu	Trifft sicher nicht zu	Trifft zu
Trifft sicher zu	Trifft sicher zu	Trifft sicher nicht zu	Trifft sicher zu
Unentschieden	Trifft sicher zu	Trifft sicher nicht zu	Trifft zu
Trifft zu	Trifft sicher zu	Trifft sicher nicht zu	Trifft zu
Trifft sicher zu	Trifft sicher zu	Trifft sicher nicht zu	Trifft sicher zu
Trifft sicher zu	Trifft sicher zu	Trifft sicher nicht zu	Trifft sicher zu
Trifft zu	Trifft zu	Trifft nicht zu	Trifft zu
Trifft sicher zu	Trifft sicher zu	Trifft sicher nicht zu	Trifft sicher zu
Trifft zu	Trifft sicher zu	Trifft nicht zu	Trifft zu
Trifft zu	Trifft zu	Trifft nicht zu	Trifft sicher zu
Trifft sicher zu	Trifft sicher zu	Trifft sicher nicht zu	Trifft sicher zu
Trifft zu	Trifft sicher zu	Trifft nicht zu	Trifft sicher zu
Trifft sicher zu	Trifft sicher zu	Trifft sicher zu	Trifft sicher zu
Trifft zu	Trifft sicher zu	Trifft sicher nicht zu	Trifft zu
Trifft zu	Trifft sicher zu	Trifft nicht zu	Trifft zu
Trifft zu	Trifft sicher zu	Trifft sicher nicht zu	Trifft sicher zu
Trifft sicher zu	Trifft sicher zu	Trifft sicher nicht zu	Trifft sicher zu
Unentschieden	Trifft sicher zu	Trifft sicher zu	Trifft sicher zu
Trifft sicher zu	Unentschieden	Trifft sicher nicht zu	Trifft sicher zu
Unentschieden	Trifft sicher zu	Trifft sicher nicht zu	Unentschieden
Trifft zu	Trifft sicher zu	Trifft sicher nicht zu	Trifft zu
Trifft sicher zu	Trifft sicher zu	Trifft sicher nicht zu	Trifft zu
Trifft zu	Trifft sicher zu	Trifft sicher nicht zu	Unentschieden
Trifft sicher zu	Trifft sicher zu	Trifft sicher nicht zu	Trifft zu
Trifft zu		Trifft sicher nicht zu	Trifft sicher zu
Trifft sicher zu	Trifft sicher zu	Trifft sicher nicht zu	Trifft sicher zu
Trifft sicher zu	Trifft sicher zu	Trifft sicher nicht zu	Trifft sicher zu
Trifft zu	Trifft sicher zu	Trifft sicher nicht zu	Trifft zu
Unentschieden	Trifft sicher zu	Trifft sicher nicht zu	Trifft zu
Trifft zu	Trifft sicher zu	Trifft sicher nicht zu	Trifft zu
Trifft zu	Trifft sicher zu	Trifft sicher nicht zu	
Trifft zu	Trifft sicher zu	Trifft sicher nicht zu	Trifft zu
Trifft sicher zu	Trifft sicher zu	Trifft sicher nicht zu	Trifft sicher zu
Trifft zu	Trifft sicher zu	Trifft sicher nicht zu	Trifft sicher zu
Trifft zu	Trifft sicher zu	Trifft nicht zu	Trifft zu
Trifft zu	Trifft sicher zu	Trifft sicher nicht zu	Trifft zu
Trifft zu	Trifft sicher zu	Trifft nicht zu	Trifft zu

Unentschieden	Trifft sicher zu	Trifft sicher nicht zu	Trifft sicher zu
Trifft zu	Trifft sicher zu	Trifft nicht zu	Trifft zu
Trifft zu	Trifft zu	Trifft nicht zu	Trifft zu
Trifft zu	Trifft sicher zu	Trifft sicher nicht zu	Trifft sicher zu
Trifft sicher zu	Trifft sicher zu	Trifft sicher nicht zu	Trifft sicher zu
Unentschieden	Trifft zu	Trifft sicher nicht zu	Trifft zu
Trifft zu	Trifft sicher zu	Trifft nicht zu	Trifft zu
Trifft sicher zu	Trifft sicher zu	Trifft sicher zu	Trifft sicher zu
Trifft zu	Trifft sicher zu	Trifft sicher nicht zu	Trifft sicher zu
Trifft sicher zu	Trifft sicher zu	Trifft sicher nicht zu	Trifft sicher zu
Trifft sicher zu	Trifft sicher zu	Trifft sicher nicht zu	Trifft sicher zu
Trifft zu	Trifft sicher zu	Trifft nicht zu	Trifft zu
Trifft zu	Trifft sicher zu	Trifft sicher nicht zu	Trifft zu
Trifft zu	Trifft zu	Trifft zu	Trifft zu
Trifft zu	Trifft sicher zu	Trifft sicher nicht zu	Trifft sicher zu
Trifft nicht zu	Trifft zu	Trifft nicht zu	Unentschieden
Unentschieden	Trifft sicher zu	Trifft sicher nicht zu	Trifft sicher zu
Trifft sicher zu	Trifft sicher zu	Trifft sicher nicht zu	Trifft sicher zu
Unentschieden	Trifft sicher zu	Trifft sicher nicht zu	Trifft sicher zu
Trifft zu	Trifft sicher zu	Trifft nicht zu	Trifft zu
Unentschieden	Trifft sicher zu	Trifft sicher nicht zu	Trifft zu
Trifft sicher zu	Trifft sicher zu	Trifft sicher nicht zu	Trifft sicher zu
Trifft zu	Trifft sicher zu	Trifft sicher nicht zu	Trifft zu
Trifft nicht zu	Trifft zu	Trifft nicht zu	Trifft sicher zu
Unentschieden	Trifft sicher zu	Trifft sicher nicht zu	Trifft sicher zu
Trifft sicher zu	Trifft sicher zu	Trifft sicher nicht zu	Trifft sicher zu
Unentschieden	Trifft zu	Trifft sicher nicht zu	Trifft zu
Trifft zu	Trifft sicher zu	Trifft sicher nicht zu	Trifft sicher zu
Trifft zu	Trifft sicher zu	Trifft sicher nicht zu	Trifft sicher zu
Trifft zu	Trifft sicher zu	Trifft nicht zu	Trifft zu
Unentschieden	Trifft zu	Trifft nicht zu	Trifft zu
Trifft sicher zu	Trifft sicher zu	Trifft nicht zu	Trifft sicher zu
Trifft zu	Trifft sicher zu	Trifft sicher nicht zu	Trifft zu
Unentschieden	Trifft sicher zu	Trifft nicht zu	Trifft sicher zu
Trifft zu	Trifft sicher zu	Trifft sicher nicht zu	Trifft sicher zu
Unentschieden	Trifft sicher zu	Trifft sicher nicht zu	Trifft sicher zu
Unentschieden	Trifft sicher zu	Trifft sicher nicht zu	Trifft zu
Trifft zu	Trifft sicher zu	Trifft sicher nicht zu	Trifft sicher zu
Trifft nicht zu	Trifft sicher zu	Trifft sicher nicht zu	Trifft sicher zu
Trifft nicht zu	Trifft sicher zu	Trifft sicher nicht zu	Trifft sicher zu
Trifft sicher zu	Trifft sicher zu	Trifft sicher nicht zu	Trifft sicher zu
Unentschieden	Trifft zu	Trifft nicht zu	Trifft zu
Trifft zu	Trifft sicher zu	Trifft sicher nicht zu	Trifft zu
Trifft sicher zu	Trifft sicher zu	Trifft sicher nicht zu	Trifft sicher zu
Unentschieden	Trifft sicher zu	Trifft nicht zu	Trifft zu
Trifft zu	Trifft sicher zu	Trifft nicht zu	Trifft nicht zu
Unentschieden	Trifft zu	Trifft nicht zu	Trifft zu
Trifft zu	Trifft zu	Trifft sicher nicht zu	Trifft zu
Trifft sicher zu	Trifft sicher zu	Trifft sicher zu	Trifft sicher zu
Unentschieden	Trifft sicher zu	Trifft sicher nicht zu	Trifft sicher zu

Trifft zu	Trifft zu	Trifft sicher nicht zu	Trifft zu
Trifft nicht zu	Trifft zu	Trifft nicht zu	Trifft zu
Trifft nicht zu	Trifft zu	Trifft nicht zu	Trifft zu
Trifft nicht zu	Trifft zu	Unentschieden	Trifft sicher zu
Unentschieden	Trifft sicher zu	Trifft sicher nicht zu	Trifft sicher zu
Trifft sicher zu	Trifft sicher zu	Unentschieden	Trifft sicher zu
Trifft zu	Trifft sicher zu	Trifft sicher nicht zu	Trifft sicher zu
Trifft nicht zu	Trifft sicher zu	Trifft sicher nicht zu	Trifft sicher zu
Trifft zu	Trifft sicher zu	Trifft sicher nicht zu	Trifft zu
Trifft zu	Trifft sicher zu	Trifft sicher nicht zu	Trifft sicher zu
Trifft zu	Trifft sicher zu	Trifft sicher nicht zu	Trifft sicher zu
Trifft zu	Trifft sicher zu	Trifft nicht zu	Trifft sicher zu
Trifft zu	Trifft zu	Trifft nicht zu	Unentschieden
Trifft zu	Trifft zu	Trifft nicht zu	Trifft zu
Trifft zu	Trifft sicher zu	Trifft sicher nicht zu	Trifft sicher zu
Unentschieden	Trifft sicher zu	Trifft sicher nicht zu	Trifft sicher zu
Unentschieden	Trifft zu	Trifft nicht zu	Trifft zu
Trifft zu	Trifft sicher zu	Trifft sicher nicht zu	Trifft sicher zu
Trifft zu	Trifft sicher zu	Trifft sicher nicht zu	Trifft sicher zu
Trifft zu	Trifft zu	Trifft nicht zu	Trifft zu
Trifft sicher zu	Trifft sicher zu	Trifft sicher nicht zu	Trifft zu
Trifft zu	Trifft sicher zu	Trifft sicher nicht zu	
Trifft nicht zu	Trifft zu	Trifft nicht zu	Trifft nicht zu
Trifft sicher zu	Trifft sicher zu	Trifft nicht zu	Trifft sicher zu
Trifft sicher zu	Trifft sicher zu	Trifft sicher nicht zu	Trifft zu
Trifft sicher zu	Trifft sicher zu	Trifft sicher zu	Trifft sicher zu
Trifft zu	Trifft sicher zu	Trifft sicher nicht zu	Trifft sicher zu
Trifft zu	Trifft sicher zu	Trifft nicht zu	Trifft sicher zu
Trifft sicher zu	Trifft sicher zu	Trifft sicher nicht zu	Trifft sicher zu
Trifft zu	Trifft sicher zu	Trifft sicher nicht zu	Trifft sicher zu
Unentschieden	Trifft zu	Trifft nicht zu	Trifft zu
Trifft sicher zu	Trifft sicher zu	Trifft sicher zu	Trifft sicher zu
Unentschieden	Trifft zu	Trifft sicher nicht zu	Trifft zu
Trifft zu	Trifft sicher zu	Trifft sicher nicht zu	Trifft sicher zu
Trifft zu	Trifft sicher zu	Trifft nicht zu	Trifft zu
Trifft sicher zu	Trifft sicher zu	Trifft sicher nicht zu	Trifft sicher zu
Trifft sicher zu	Trifft sicher zu	Trifft nicht zu	Trifft sicher zu
Trifft zu	Trifft zu	Trifft sicher nicht zu	Trifft sicher zu
Trifft sicher zu	Trifft sicher zu	Trifft sicher nicht zu	Trifft sicher zu
Trifft zu	Trifft sicher zu	Trifft sicher nicht zu	Trifft zu
Trifft sicher zu	Trifft sicher zu	Trifft sicher nicht zu	Trifft zu
Trifft zu	Trifft sicher zu	Trifft nicht zu	Trifft zu
Trifft zu	Trifft sicher zu	Trifft sicher nicht zu	Trifft sicher zu
Trifft zu	Trifft sicher zu	Trifft nicht zu	Trifft zu
Trifft sicher zu	Trifft sicher zu	Trifft nicht zu	Trifft zu
Trifft zu	Trifft sicher zu	Trifft sicher nicht zu	Trifft sicher zu
Trifft zu	Trifft sicher zu	Trifft sicher nicht zu	Trifft sicher zu
Trifft zu	Trifft sicher zu	Trifft sicher nicht zu	Trifft sicher zu
Trifft sicher zu	Trifft sicher zu	Trifft sicher nicht zu	Trifft sicher zu

Trifft zu	Trifft sicher zu	Trifft sicher nicht zu	Trifft sicher zu
Trifft zu	Trifft sicher zu	Trifft sicher nicht zu	Trifft zu
Unentschieden	Trifft sicher zu	Trifft nicht zu	Trifft zu
Unentschieden	Trifft sicher zu	Trifft sicher nicht zu	Trifft sicher zu
Unentschieden	Trifft zu	Trifft nicht zu	Trifft zu
Trifft zu	Trifft sicher zu	Trifft nicht zu	Trifft zu
Trifft sicher zu	Trifft sicher zu	Trifft sicher nicht zu	Trifft sicher zu
Trifft zu	Trifft sicher zu	Trifft sicher nicht zu	Trifft sicher zu
Unentschieden	Trifft sicher zu	Trifft nicht zu	Trifft sicher zu
Trifft sicher zu	Trifft sicher zu	Trifft sicher zu	Trifft sicher zu
Trifft sicher zu	Trifft sicher zu	Trifft sicher nicht zu	Trifft sicher zu
Trifft sicher zu	Trifft sicher zu	Trifft sicher nicht zu	
Trifft sicher zu	Trifft sicher zu		Trifft sicher zu
Trifft zu	Trifft sicher zu	Trifft sicher nicht zu	Trifft sicher zu
Trifft zu	Trifft sicher zu	Trifft sicher nicht zu	Trifft zu
Trifft sicher zu	Trifft zu	Trifft nicht zu	Trifft sicher zu
Trifft sicher zu	Trifft sicher zu	Trifft sicher nicht zu	Unentschieden
Trifft zu	Trifft sicher zu	Trifft sicher nicht zu	Trifft sicher zu

Ich würde mir wünscher	Die studentische Leitung	Das Seminar war profes:	Während des Seminars f
Trifft nicht zu	Trifft zu	Trifft zu	Trifft zu
Trifft sicher zu	Trifft zu	Trifft sicher zu	Trifft nicht zu
Trifft sicher zu	Trifft sicher zu	Trifft sicher zu	Trifft zu

Trifft zu	Trifft zu	Trifft zu	Unentschieden
Trifft sicher zu	Trifft sicher zu	Trifft sicher zu	Unentschieden
Trifft zu	Unentschieden	Trifft sicher zu	Trifft nicht zu
Trifft sicher zu	Trifft sicher zu	Trifft sicher zu	Trifft sicher zu
Trifft sicher zu	Trifft sicher zu	Trifft sicher zu	Trifft sicher zu
Trifft zu	Trifft sicher zu	Trifft sicher zu	Trifft zu
Trifft sicher zu	Trifft sicher zu	Trifft sicher zu	Trifft zu
Trifft sicher zu	Trifft sicher zu	Trifft sicher zu	Trifft sicher zu
Trifft zu	Trifft sicher zu	Trifft sicher zu	Trifft sicher zu
Trifft sicher zu	Trifft sicher zu	Trifft sicher zu	Trifft zu
Trifft sicher zu	Trifft sicher zu	Trifft sicher zu	Trifft sicher zu
Trifft sicher zu	Trifft zu	Trifft zu	Trifft zu
Trifft zu	Trifft sicher zu	Trifft sicher zu	Trifft zu
Trifft zu	Trifft zu	Trifft zu	Trifft zu
Trifft sicher zu	Trifft sicher zu	Trifft sicher zu	Trifft zu
Trifft zu	Trifft zu	Trifft sicher zu	Trifft nicht zu
Trifft sicher zu	Trifft sicher zu	Trifft sicher zu	Trifft sicher zu
Trifft sicher zu	Trifft sicher zu	Trifft sicher zu	Trifft sicher zu
Trifft sicher zu	Trifft sicher zu	Trifft sicher zu	Trifft zu
Trifft zu	Trifft sicher zu	Trifft sicher zu	Trifft zu
Trifft sicher zu	Trifft sicher zu	Trifft sicher zu	Trifft zu
Trifft zu	Trifft sicher zu	Trifft sicher zu	Trifft zu
Trifft sicher zu	Trifft zu	Trifft sicher zu	Unentschieden
Trifft sicher zu	Trifft sicher zu	Trifft sicher zu	Unentschieden
Trifft sicher zu	Trifft sicher zu	Trifft sicher zu	Trifft zu
Trifft sicher zu	Trifft sicher zu	Trifft zu	Trifft sicher zu
Trifft zu	Trifft zu	Trifft zu	Trifft zu
Trifft sicher zu	Trifft sicher zu	Trifft sicher zu	Trifft sicher zu
Unentschieden	Trifft sicher zu	Trifft sicher zu	Trifft zu
Trifft sicher zu	Trifft sicher zu	Trifft sicher zu	Trifft sicher zu
Trifft zu	Trifft zu	Trifft sicher zu	Unentschieden
Trifft sicher zu	Trifft sicher zu	Trifft sicher zu	Trifft zu
Trifft sicher zu	Trifft sicher zu	Trifft sicher zu	Trifft sicher zu
Trifft sicher zu	Trifft sicher zu	Trifft sicher zu	Trifft sicher zu
Trifft sicher zu	Trifft sicher zu	Trifft zu	Trifft zu
Trifft sicher zu	Trifft sicher zu	Trifft sicher zu	Trifft zu
Trifft zu	Trifft sicher zu	Trifft sicher zu	Trifft zu
Trifft zu	Trifft sicher zu	Trifft sicher zu	Trifft sicher zu
Trifft zu	Trifft sicher zu	Trifft sicher zu	Trifft sicher zu
Trifft sicher zu	Trifft sicher zu	Trifft sicher zu	Trifft sicher zu
Trifft sicher zu	Trifft sicher zu	Trifft sicher zu	Trifft sicher zu
Trifft zu	Trifft zu	Trifft zu	Trifft zu
Trifft sicher zu	Trifft sicher zu	Trifft sicher zu	Unentschieden
Trifft zu	Trifft sicher zu	Trifft sicher zu	Trifft sicher zu
Trifft sicher zu	Trifft sicher zu	Trifft sicher zu	Trifft sicher zu
Trifft zu	Trifft sicher zu	Trifft sicher zu	Trifft zu
Unentschieden	Trifft zu	Trifft sicher zu	Trifft nicht zu
Trifft zu	Trifft zu	Trifft zu	Trifft sicher zu
Trifft sicher zu	Trifft sicher zu	Trifft sicher zu	Trifft sicher zu
Unentschieden	Trifft sicher zu	Trifft sicher zu	Trifft zu

Trifft zu	Trifft zu	Trifft zu	Trifft zu
Unentschieden	Trifft zu	Trifft zu	Unentschieden
Trifft zu	Trifft zu	Trifft zu	Unentschieden
Trifft sicher nicht zu	Trifft sicher zu	Trifft sicher zu	Trifft zu
Unentschieden	Trifft sicher zu	Trifft sicher zu	Trifft zu
Trifft sicher zu	Trifft sicher zu	Trifft sicher zu	Trifft sicher zu
Trifft sicher zu	Trifft sicher zu	Trifft sicher zu	Trifft sicher zu
Trifft sicher zu	Trifft sicher zu	Trifft sicher zu	Trifft sicher zu
Trifft sicher zu	Trifft sicher zu	Trifft sicher zu	Trifft zu
Trifft sicher zu	Trifft sicher zu	Trifft sicher zu	Trifft zu
Trifft sicher zu	Trifft sicher zu	Trifft sicher zu	Trifft sicher zu
Trifft zu	Trifft zu	Trifft zu	Unentschieden
Trifft zu	Unentschieden	Trifft zu	Trifft sicher zu
Trifft sicher zu	Trifft zu	Trifft sicher zu	Trifft zu
Trifft zu	Trifft sicher zu	Trifft sicher zu	Trifft zu
Trifft zu	Trifft sicher zu	Trifft sicher zu	Trifft zu
Unentschieden	Trifft sicher zu	Trifft sicher zu	Unentschieden
Trifft zu	Trifft zu	Trifft sicher zu	Trifft zu
Trifft sicher zu	Trifft sicher zu	Trifft sicher zu	Trifft sicher zu
Trifft sicher zu	Trifft sicher zu	Trifft sicher zu	Trifft zu
Trifft sicher zu	Trifft sicher zu	Trifft sicher zu	Trifft zu
Trifft sicher zu	Trifft sicher zu	Trifft sicher zu	Trifft zu
Trifft zu	Trifft sicher zu	Trifft zu	Trifft zu
Unentschieden	Trifft sicher zu	Trifft sicher zu	Trifft zu
Trifft sicher zu	Trifft sicher zu	Trifft sicher zu	Trifft sicher zu
Trifft sicher zu	Trifft sicher zu	Trifft sicher zu	Trifft sicher zu
Trifft zu	Trifft zu	Trifft sicher zu	Trifft zu
Unentschieden	Trifft zu	Trifft zu	Trifft zu
Trifft sicher zu	Trifft zu	Trifft sicher zu	Trifft zu
Unentschieden	Trifft sicher zu	Unentschieden	Unentschieden
Trifft zu	Trifft zu	Trifft zu	Trifft zu
Trifft sicher zu	Trifft sicher zu	Trifft sicher zu	Trifft sicher zu
Trifft zu	Trifft zu	Trifft zu	Trifft zu
Trifft zu	Trifft sicher zu	Trifft sicher zu	Trifft zu
Unentschieden	Trifft sicher zu	Trifft sicher zu	Unentschieden
Trifft sicher zu	Trifft sicher zu	Trifft sicher zu	Trifft sicher zu
Trifft zu	Trifft sicher zu	Trifft sicher zu	Trifft zu
Trifft zu	Trifft sicher zu	Trifft sicher zu	Trifft zu
Trifft sicher zu	Trifft sicher zu	Trifft sicher zu	Trifft zu
Trifft sicher zu	Trifft sicher zu	Trifft sicher zu	Trifft zu
Trifft sicher zu	Trifft sicher zu	Trifft sicher zu	Trifft zu
Trifft sicher zu	Trifft zu	Trifft sicher zu	Trifft zu
Trifft zu	Trifft sicher zu	Trifft sicher zu	Trifft zu
Unentschieden	Trifft zu	Trifft zu	Trifft zu
Trifft zu	Unentschieden	Trifft zu	Trifft zu
Trifft sicher zu	Trifft sicher zu	Trifft sicher zu	Trifft zu
Trifft sicher zu	Trifft sicher zu	Trifft sicher zu	Unentschieden
Trifft sicher zu	Trifft sicher zu	Trifft sicher zu	Trifft zu
Unentschieden	Trifft sicher zu	Trifft sicher zu	Unentschieden

Trifft sicher zu	Trifft sicher zu	Trifft sicher zu	Trifft sicher zu
Trifft zu	Trifft zu	Trifft zu	Unentschieden
Trifft sicher zu	Trifft sicher zu	Trifft zu	Unentschieden
Unentschieden	Trifft sicher zu	Trifft sicher zu	Trifft sicher zu
Trifft sicher zu	Trifft sicher zu	Trifft sicher zu	Trifft zu
Trifft sicher zu	Trifft zu	Trifft zu	Trifft zu
Trifft sicher zu	Trifft sicher zu	Trifft sicher zu	Trifft zu
Trifft sicher zu	Trifft sicher zu	Trifft sicher zu	Trifft sicher zu
Trifft sicher zu	Trifft sicher zu	Trifft sicher zu	Trifft zu
Trifft sicher zu	Trifft sicher zu	Trifft sicher zu	Trifft sicher zu
Trifft sicher zu	Trifft zu	Trifft sicher zu	Trifft sicher zu
Trifft sicher zu	Trifft sicher zu	Trifft sicher zu	Trifft sicher zu
Trifft sicher zu	Trifft sicher zu	Trifft sicher zu	Trifft sicher zu
Trifft sicher zu	Trifft sicher zu	Trifft zu	Trifft sicher zu
Trifft zu	Trifft zu	Trifft sicher zu	Trifft zu
Trifft zu	Trifft sicher zu	Trifft sicher zu	Trifft zu
Trifft zu	Trifft sicher zu	Trifft sicher zu	Trifft zu
Trifft sicher zu	Trifft sicher zu	Trifft sicher zu	Trifft zu

Ich befürworte es, dass i	Nach Teilnahme an dies	Ich konnte bereits neue	Durch das Seminar fällt
Trifft sicher zu	Trifft zu	Trifft zu	Trifft zu
Trifft zu	Trifft zu	Trifft sicher zu	Trifft zu
Trifft zu	Unentschieden	Trifft zu	Trifft sicher zu

Unentschieden	Trifft zu	Trifft zu	Trifft zu
Trifft zu	Unentschieden	Trifft zu	Trifft zu
Trifft zu	Trifft zu	Trifft nicht zu	Unentschieden
Trifft sicher zu	Trifft sicher zu	Trifft sicher zu	Trifft sicher zu
Trifft sicher zu	Trifft sicher zu	Trifft sicher zu	Trifft sicher zu
Trifft zu	Trifft zu	Trifft sicher zu	Trifft zu
Trifft sicher zu	Unentschieden	Trifft zu	Unentschieden
Trifft sicher zu	Trifft sicher zu	Trifft sicher zu	Trifft sicher zu
Trifft sicher zu	Trifft sicher zu	Trifft sicher zu	Trifft sicher zu
Trifft sicher zu	Trifft sicher zu	Trifft sicher zu	Trifft zu
Trifft sicher zu	Trifft sicher zu	Trifft sicher zu	Trifft sicher zu
Trifft zu	Trifft sicher zu	Trifft zu	Trifft zu
Trifft zu	Unentschieden	Trifft zu	Unentschieden
Trifft zu	Trifft zu	Trifft zu	Trifft zu
Trifft sicher zu	Trifft sicher zu	Trifft sicher zu	Trifft sicher zu
Trifft zu	Unentschieden	Trifft zu	Trifft zu
Trifft sicher zu	Trifft sicher zu	Trifft sicher zu	Trifft sicher zu
Trifft sicher zu	Unentschieden	Trifft zu	Trifft zu
Trifft zu	Unentschieden	Trifft zu	Trifft zu
Trifft zu	Trifft zu	Trifft zu	Trifft zu
Trifft sicher zu	Trifft sicher zu	Trifft sicher zu	Trifft sicher zu
Trifft sicher zu	Trifft zu	Trifft sicher zu	Trifft zu
Trifft zu	Trifft sicher zu	Trifft sicher zu	Trifft zu
Unentschieden	Trifft sicher zu	Trifft sicher zu	Trifft zu
Trifft sicher zu	Trifft zu	Trifft sicher zu	Trifft sicher zu
Unentschieden	Unentschieden	Trifft zu	Trifft nicht zu
Trifft zu	Unentschieden	Trifft zu	Unentschieden
Trifft sicher zu	Unentschieden	Trifft sicher zu	Trifft zu
Trifft zu	Unentschieden	Trifft sicher zu	Trifft zu
Trifft sicher zu	Unentschieden	Trifft zu	Trifft zu
Trifft zu	Trifft zu	Trifft sicher zu	Unentschieden
Trifft sicher zu	Unentschieden	Trifft sicher zu	Trifft sicher zu
Trifft sicher zu	Unentschieden	Trifft sicher zu	Unentschieden
Trifft zu	Unentschieden	Trifft sicher zu	Trifft zu
Trifft zu	Trifft nicht zu	Trifft zu	Trifft zu
Trifft zu	Unentschieden	Trifft zu	Trifft zu
Trifft sicher zu	Unentschieden	Trifft sicher zu	Trifft zu
Trifft sicher zu	Trifft sicher zu	Trifft sicher zu	Trifft sicher zu
Trifft zu	Trifft sicher zu	Trifft sicher zu	Trifft zu
Trifft sicher zu	Unentschieden	Unentschieden	Trifft zu
Trifft sicher zu	Trifft sicher zu	Trifft sicher zu	Trifft sicher zu
Trifft zu	Trifft zu	Trifft zu	Trifft zu
Trifft sicher zu	Unentschieden	Trifft zu	Trifft zu
Trifft sicher zu	Unentschieden	Trifft sicher zu	Unentschieden
Trifft sicher zu	Trifft zu	Trifft sicher zu	Trifft sicher zu
Trifft sicher zu	Unentschieden	Trifft zu	Trifft zu
Trifft sicher zu	Trifft sicher zu	Trifft zu	Trifft zu
Trifft sicher zu	Trifft zu	Trifft zu	Trifft zu
Trifft sicher zu	Trifft sicher zu	Trifft sicher zu	Trifft sicher zu
Trifft sicher zu	Trifft sicher zu	Trifft sicher zu	Trifft sicher zu

Trifft sicher zu	Unentschieden	Trifft zu	Trifft zu
Trifft sicher zu	Trifft sicher zu	Trifft zu	Unentschieden
Trifft zu	Trifft sicher zu	Unentschieden	Unentschieden
Trifft sicher zu	Unentschieden	Trifft zu	Trifft zu
Trifft zu	Unentschieden	Trifft sicher zu	Trifft zu
Trifft sicher zu	Unentschieden	Trifft sicher zu	Trifft sicher zu
Trifft sicher zu	Trifft sicher zu	Trifft sicher zu	Trifft sicher zu
Trifft sicher zu	Unentschieden	Trifft sicher zu	Trifft sicher zu
Trifft sicher zu	Trifft zu	Unentschieden	Trifft zu
Trifft zu	Trifft sicher zu	Trifft sicher zu	Trifft sicher zu
Trifft sicher zu	Trifft zu	Trifft zu	Trifft zu
Trifft zu	Unentschieden	Trifft zu	Unentschieden
Trifft zu	Trifft zu	Trifft sicher zu	Trifft zu
Trifft sicher zu	Trifft zu	Trifft zu	Unentschieden
Trifft sicher zu	Trifft zu	Trifft sicher zu	Trifft sicher zu
Trifft sicher zu	Trifft sicher zu	Trifft zu	Trifft zu
Trifft zu	Unentschieden	Unentschieden	Unentschieden
Trifft sicher zu	Unentschieden	Trifft zu	Trifft zu
Trifft sicher zu	Unentschieden	Trifft sicher zu	Trifft sicher zu
Trifft zu	Unentschieden	Trifft zu	Trifft zu
Trifft sicher zu	Trifft sicher zu	Trifft sicher zu	Trifft sicher zu
Trifft sicher zu	Trifft zu	Trifft sicher zu	Trifft zu
Trifft zu	Unentschieden	Trifft zu	Trifft zu
Trifft zu	Trifft zu	Trifft sicher zu	Unentschieden
Trifft sicher zu	Trifft sicher zu	Trifft sicher zu	Trifft sicher zu
Trifft sicher zu	Trifft sicher zu	Trifft sicher zu	Trifft zu
Trifft sicher zu	Trifft zu	Trifft zu	Trifft zu
Trifft sicher zu	Trifft sicher zu	Trifft sicher zu	Trifft zu
Trifft sicher zu	Trifft sicher zu	Trifft sicher zu	Trifft sicher zu
Trifft zu	Unentschieden	Trifft zu	Trifft nicht zu
Trifft zu	Trifft zu	Trifft zu	Trifft zu
Trifft sicher zu	Trifft sicher zu	Trifft sicher zu	Trifft sicher zu
Trifft zu	Trifft zu	Trifft zu	Trifft zu
Trifft sicher zu	Unentschieden	Trifft zu	Trifft zu
Trifft sicher zu	Trifft zu	Trifft zu	Trifft zu
	Trifft sicher zu	Trifft sicher zu	Trifft sicher zu
Trifft zu	Trifft zu	Trifft sicher zu	Trifft sicher zu
Trifft sicher zu	Trifft zu	Trifft zu	Trifft zu
Trifft sicher zu	Trifft zu	Trifft sicher zu	Trifft zu
Trifft sicher zu	Unentschieden	Trifft zu	Trifft zu
Trifft sicher zu	Trifft sicher zu	Trifft zu	Trifft zu
Trifft sicher zu	Trifft sicher zu	Trifft sicher zu	Trifft zu
Trifft zu	Trifft zu	Trifft zu	Trifft zu
Trifft sicher zu	Trifft zu	Trifft zu	Trifft zu
Trifft zu	Unentschieden	Unentschieden	Trifft sicher zu
Trifft zu	Trifft sicher zu	Trifft zu	Unentschieden
Trifft sicher zu	Trifft zu	Trifft zu	Trifft sicher zu
Trifft sicher zu	Trifft sicher zu	Trifft sicher zu	Trifft sicher zu
Trifft zu	Trifft nicht zu	Trifft zu	Trifft zu

Trifft sicher zu	Unentschieden	Trifft zu	Trifft zu
Trifft zu	Unentschieden	Trifft zu	Unentschieden
Trifft sicher zu	Trifft zu	Trifft sicher zu	Trifft sicher zu
Trifft sicher zu	Trifft zu	Trifft sicher zu	Trifft sicher zu
Trifft zu	Unentschieden	Trifft zu	Trifft zu
Trifft sicher zu	Trifft sicher zu	Trifft zu	Trifft sicher zu
Trifft sicher zu	Trifft sicher zu	Trifft sicher zu	Trifft sicher zu
Trifft sicher zu	Trifft sicher zu	Trifft sicher zu	Trifft sicher zu
Trifft sicher zu	Trifft sicher zu	Trifft sicher zu	Trifft zu
Trifft sicher zu	Trifft zu	Trifft sicher zu	Trifft sicher zu
Trifft sicher zu	Trifft sicher zu	Trifft sicher zu	Trifft sicher zu
Trifft sicher zu	Trifft sicher zu	Trifft sicher zu	Trifft zu
Trifft sicher zu	Unentschieden	Trifft sicher zu	Trifft sicher zu
Trifft sicher zu	Trifft sicher zu	Trifft zu	Unentschieden
Trifft zu	Trifft zu	Unentschieden	Trifft sicher zu
Trifft sicher zu	Unentschieden	Trifft zu	Trifft zu
Trifft sicher zu	Unentschieden	Trifft zu	Trifft sicher zu
Trifft zu	Trifft sicher zu	Trifft sicher zu	Trifft zu

Das Erlernen von Sympt	Durch das Tool wurde da	Ich würde mir die Nutzu	Ich würde mir die Nutzu
	Trifft sicher zu	Trifft sicher zu	Trifft sicher zu
	Trifft sicher zu	Trifft sicher zu	Trifft sicher zu
	Trifft sicher zu	Trifft sicher zu	Trifft sicher zu
	Trifft sicher zu	Trifft zu	Trifft zu
	Trifft sicher zu	Trifft sicher zu	Trifft sicher zu
	Trifft sicher zu	Trifft sicher zu	Trifft zu
	Trifft sicher zu	Trifft sicher zu	Trifft zu
	Trifft zu	Trifft zu	Trifft zu
	Trifft zu	Trifft zu	Trifft zu
	Trifft sicher zu	Trifft sicher zu	Trifft sicher zu
	Trifft sicher zu	Trifft sicher zu	Unentschieden
	Trifft sicher zu	Trifft sicher zu	Trifft sicher zu
	Trifft sicher zu	Trifft sicher zu	Trifft sicher zu
	Trifft zu	Trifft sicher zu	Trifft zu
	Unentschieden	Trifft zu	Trifft zu
	Trifft sicher zu	Trifft sicher zu	Trifft sicher zu
	Trifft sicher zu	Trifft zu	Trifft sicher zu
	Trifft sicher zu	Trifft sicher zu	Trifft sicher zu
	Trifft sicher zu	Trifft sicher zu	Trifft sicher zu
	Trifft sicher zu	Trifft sicher zu	Trifft sicher zu
	Trifft sicher zu	Trifft sicher zu	Trifft sicher zu
	Trifft sicher zu	Trifft sicher zu	Trifft sicher zu
	Trifft sicher zu	Trifft sicher zu	Trifft sicher zu
	Trifft sicher zu	Trifft sicher zu	Trifft sicher zu
	Trifft zu	Trifft zu	Trifft zu
	Trifft sicher zu	Trifft sicher zu	Unentschieden
	Unentschieden	Unentschieden	Unentschieden
	Trifft zu	Trifft zu	Unentschieden
	Trifft sicher zu	Trifft sicher zu	Trifft sicher zu
	Trifft sicher zu	Trifft sicher zu	Trifft sicher zu
	Trifft zu	Trifft zu	Trifft zu
	Trifft sicher zu	Trifft sicher zu	Trifft sicher zu
	Unentschieden		
	Trifft sicher zu	Trifft sicher zu	Trifft sicher zu
	Trifft sicher zu	Trifft sicher zu	Trifft sicher zu
	Trifft zu	Unentschieden	Unentschieden
	Trifft sicher zu	Trifft sicher zu	Trifft sicher zu
	Trifft sicher zu	Trifft sicher zu	Trifft sicher zu
	Trifft sicher zu	Trifft sicher zu	Trifft sicher zu
	Trifft sicher zu	Trifft sicher zu	Trifft sicher zu
	Trifft sicher zu	Trifft sicher zu	Trifft sicher zu
Trifft sicher zu	Trifft sicher zu	Trifft zu	Trifft zu
Trifft zu	Trifft sicher zu	Trifft sicher zu	Trifft nicht zu
Trifft zu	Trifft sicher zu	Trifft sicher zu	Unentschieden

Trifft zu	Trifft zu	Trifft zu	Trifft zu
Trifft zu	Trifft sicher zu	Trifft sicher zu	Trifft sicher zu
Unentschieden	Trifft sicher zu	Trifft sicher zu	Trifft sicher zu
Trifft sicher zu	Trifft sicher zu	Trifft sicher zu	Trifft sicher zu
Trifft sicher zu	Trifft sicher zu	Trifft sicher zu	Trifft sicher zu
Trifft sicher zu	Trifft sicher zu	Trifft sicher zu	Trifft sicher zu
Trifft zu	Trifft sicher zu	Trifft sicher zu	Trifft sicher zu
Trifft sicher zu	Trifft sicher zu	Trifft sicher zu	Trifft sicher zu
Trifft sicher zu	Trifft zu	Trifft zu	Trifft sicher zu
Trifft sicher zu	Trifft sicher zu	Trifft sicher zu	Trifft sicher zu
Trifft sicher zu	Trifft sicher zu	Trifft sicher zu	Trifft sicher zu
Trifft zu	Trifft sicher zu	Trifft sicher zu	Trifft sicher zu
Unentschieden	Trifft sicher zu	Trifft sicher zu	Trifft sicher zu
Trifft zu	Trifft sicher zu	Trifft sicher zu	Trifft sicher zu
Trifft sicher zu	Trifft sicher zu	Trifft sicher zu	Trifft sicher zu
Trifft zu	Trifft sicher zu	Trifft zu	Trifft zu
Trifft sicher zu	Trifft sicher zu	Trifft sicher zu	Trifft sicher zu
Trifft sicher zu	Trifft zu	Trifft zu	Trifft zu
Trifft zu	Trifft sicher zu	Trifft sicher zu	Trifft sicher zu
	Trifft sicher zu	Trifft sicher zu	Trifft sicher zu
Trifft sicher zu	Trifft sicher zu	Trifft sicher zu	Trifft zu
Trifft sicher zu	Trifft sicher zu	Trifft sicher zu	Trifft zu
Trifft zu	Trifft zu	Trifft zu	Unentschieden
Trifft zu	Trifft zu	Trifft sicher zu	Trifft sicher zu
Trifft sicher zu	Trifft sicher zu	Trifft sicher zu	Trifft sicher zu
Unentschieden	Trifft sicher zu	Trifft sicher zu	Trifft sicher zu
Unentschieden	Trifft sicher zu	Trifft sicher zu	Trifft zu
Trifft sicher zu	Trifft sicher zu	Trifft sicher zu	Trifft sicher zu
Trifft zu	Trifft sicher zu	Trifft sicher zu	Trifft zu
Trifft zu	Unentschieden		Trifft sicher zu
Trifft sicher zu	Trifft sicher zu	Trifft sicher zu	Trifft zu
Trifft sicher zu	Trifft sicher zu	Trifft sicher zu	Trifft sicher zu
Trifft sicher zu	Trifft sicher zu	Trifft sicher zu	Trifft zu
Trifft sicher zu	Trifft sicher zu	Trifft sicher zu	Trifft sicher zu
Trifft zu	Trifft sicher zu	Trifft zu	Trifft zu
Trifft sicher zu	Trifft sicher zu	Trifft sicher zu	Trifft sicher zu
Trifft sicher zu	Trifft sicher zu	Trifft sicher zu	Trifft sicher zu
Trifft sicher zu	Trifft sicher zu	Trifft sicher zu	Unentschieden
Trifft sicher zu	Trifft sicher zu	Trifft sicher zu	Trifft zu
Trifft sicher zu	Trifft sicher zu	Trifft sicher zu	Trifft sicher zu
Trifft sicher zu	Trifft sicher zu	Trifft sicher zu	Trifft sicher zu
Trifft zu	Trifft sicher zu	Trifft sicher zu	Trifft sicher zu
Trifft zu	Trifft sicher zu	Trifft sicher zu	Trifft sicher zu
Trifft sicher zu	Trifft sicher zu	Trifft sicher zu	Trifft sicher zu
Trifft sicher zu	Trifft sicher zu	Trifft sicher zu	Trifft sicher zu
Trifft sicher zu	Trifft sicher zu	Trifft sicher zu	Trifft sicher zu
Trifft sicher zu	Trifft sicher zu	Unentschieden	Unentschieden
Trifft zu	Unentschieden	Unentschieden	Unentschieden
Trifft sicher zu			
Trifft sicher zu	Trifft sicher zu	Trifft sicher zu	Trifft sicher zu

Trifft zu	Trifft sicher zu	Trifft sicher zu	Trifft sicher zu
Trifft zu	Trifft sicher zu	Trifft sicher zu	Trifft sicher zu
Trifft zu	Trifft sicher zu	Trifft zu	Trifft zu
Trifft sicher zu	Trifft sicher zu	Trifft sicher zu	Trifft sicher zu
Trifft zu	Trifft sicher zu	Trifft sicher zu	Trifft sicher zu
Trifft sicher zu	Trifft sicher zu	Trifft sicher zu	Trifft sicher zu
Trifft sicher zu	Trifft sicher zu	Trifft sicher zu	Trifft sicher zu
Trifft sicher zu	Trifft sicher zu	Trifft sicher zu	Trifft zu
Trifft sicher zu	Trifft sicher zu	Trifft sicher zu	Trifft sicher zu
Trifft sicher zu	Trifft sicher zu	Trifft sicher zu	Trifft sicher zu
Trifft sicher zu	Trifft sicher zu	Trifft sicher zu	Trifft sicher zu
Trifft zu	Trifft zu	Trifft zu	Trifft zu
Trifft sicher zu	Trifft zu	Trifft zu	Trifft nicht zu
Trifft zu	Trifft sicher zu	Trifft sicher zu	Trifft sicher zu
Trifft sicher zu	Trifft sicher zu	Trifft sicher zu	Trifft sicher zu
Trifft sicher zu	Trifft sicher zu	Trifft sicher zu	Trifft sicher zu
Trifft zu	Trifft zu	Trifft zu	Trifft zu
Trifft zu	Trifft sicher zu	Trifft sicher zu	Trifft sicher zu
Trifft sicher zu	Trifft sicher zu	Trifft sicher zu	Trifft sicher zu
Trifft zu	Trifft zu	Trifft zu	Trifft zu
Trifft sicher zu	Trifft sicher zu	Trifft sicher zu	Trifft zu
Trifft zu	Trifft sicher zu	Trifft sicher zu	Trifft zu
Trifft zu	Trifft sicher zu	Trifft sicher zu	Trifft sicher zu
Trifft zu	Trifft sicher zu	Unentschieden	Unentschieden
Trifft sicher zu	Trifft sicher zu	Trifft sicher zu	Trifft sicher zu
Trifft sicher zu	Trifft sicher zu	Trifft sicher zu	Trifft sicher zu
Trifft sicher zu	Trifft sicher zu	Trifft sicher zu	Trifft sicher zu
Trifft sicher zu	Trifft sicher zu		Trifft sicher zu
Trifft sicher zu	Trifft sicher zu	Trifft sicher zu	Trifft sicher zu
Trifft zu	Trifft sicher zu	Trifft sicher zu	Trifft sicher zu
Trifft zu	Trifft zu	Trifft zu	Trifft zu
Trifft sicher zu	Trifft sicher zu	Trifft sicher zu	Trifft sicher zu
Trifft zu	Trifft sicher zu	Trifft sicher zu	Trifft sicher zu
Trifft zu	Trifft sicher zu	Trifft sicher zu	Trifft sicher zu
Trifft zu	Trifft zu	Trifft zu	Trifft zu
Trifft sicher zu	Trifft sicher zu	Trifft sicher zu	Trifft sicher zu
Trifft sicher zu	Trifft zu	Unentschieden	Trifft nicht zu
Trifft zu	Trifft sicher zu	Trifft sicher zu	Trifft sicher zu
Trifft sicher zu	Trifft sicher zu	Trifft sicher zu	Trifft sicher zu
Trifft zu	Trifft sicher zu	Trifft sicher zu	Trifft sicher zu
Trifft sicher zu	Trifft sicher zu	Trifft sicher zu	Trifft sicher zu
Trifft zu	Trifft sicher zu	Trifft sicher zu	Trifft sicher zu
Trifft zu	Trifft sicher zu	Trifft sicher zu	Trifft sicher zu
Trifft sicher zu	Trifft sicher zu	Trifft sicher zu	Trifft zu
Trifft zu	Trifft sicher zu	Trifft sicher zu	Trifft sicher zu
Trifft sicher zu	Trifft sicher zu	Trifft sicher zu	Trifft zu
Trifft sicher zu	Trifft sicher zu	Trifft sicher zu	Trifft sicher zu
Trifft sicher zu	Trifft sicher zu	Trifft sicher zu	Trifft sicher zu
Trifft sicher zu	Trifft zu	Trifft sicher zu	Trifft zu

Trifft zu	Trifft sicher zu	Trifft sicher zu	Trifft sicher zu
Trifft zu	Trifft zu	Trifft zu	Trifft zu
Trifft sicher zu	Trifft sicher zu	Trifft sicher zu	Trifft sicher zu
Trifft sicher zu	Trifft sicher zu	Trifft sicher zu	Trifft sicher zu
Trifft sicher zu	Trifft sicher zu	Trifft sicher zu	Trifft sicher zu
Trifft sicher zu	Trifft sicher zu	Trifft sicher zu	Trifft sicher zu
Trifft sicher zu	Trifft sicher zu	Trifft sicher zu	Trifft sicher zu
Trifft sicher zu	Trifft sicher zu	Trifft sicher zu	Trifft sicher zu
Trifft sicher zu	Trifft sicher zu	Trifft sicher zu	Trifft sicher zu
Trifft sicher zu	Trifft sicher zu	Trifft sicher zu	Trifft sicher zu
Trifft sicher zu	Trifft sicher zu	Trifft sicher zu	Trifft sicher zu
Trifft sicher zu	Trifft sicher zu	Trifft sicher zu	Trifft sicher zu
Trifft sicher zu	Trifft sicher zu	Trifft sicher zu	Trifft sicher zu
Trifft zu	Trifft sicher zu	Trifft sicher zu	Trifft sicher zu
Trifft sicher zu	Unentschieden	Unentschieden	Unentschieden
Trifft zu	Trifft sicher zu	Trifft sicher zu	Trifft sicher zu
Trifft sicher zu	Trifft sicher zu	Trifft sicher zu	Trifft sicher zu
Trifft sicher zu	Trifft sicher zu	Trifft zu	Trifft zu

Ein Seminar ohne die Ve	Ein Vorteil der Lehre dur	Ein Nachteil der Lehre d	Um die fehlende klinisch
Trifft sicher nicht zu	Unentschieden	Trifft zu	Trifft sicher zu
Trifft sicher nicht zu	Trifft zu	Trifft nicht zu	Trifft zu
Trifft sicher nicht zu	Trifft zu	Trifft nicht zu	Trifft sicher zu
Trifft nicht zu	Trifft nicht zu	Trifft sicher nicht zu	Trifft zu
Trifft sicher nicht zu	Unentschieden	Trifft nicht zu	Trifft sicher zu
Trifft sicher nicht zu	Trifft sicher zu	Trifft nicht zu	Trifft sicher zu
Trifft sicher nicht zu	Trifft sicher zu	Trifft nicht zu	Trifft sicher zu
Trifft nicht zu	Trifft zu	Trifft nicht zu	Trifft zu
Trifft nicht zu	Trifft zu	Trifft nicht zu	Trifft sicher zu
Trifft nicht zu	Trifft zu	Unentschieden	Trifft sicher zu
	Trifft sicher zu	Trifft nicht zu	Trifft sicher zu
Trifft sicher nicht zu	Trifft sicher zu	Trifft sicher nicht zu	Trifft zu
Trifft sicher nicht zu	Trifft sicher zu	Trifft sicher nicht zu	Trifft zu
	Trifft zu	Trifft zu	Trifft sicher zu
Trifft sicher nicht zu	Unentschieden	Unentschieden	Trifft sicher zu
Trifft sicher nicht zu	Trifft sicher zu	Trifft nicht zu	Trifft sicher zu
	Trifft sicher zu	Trifft nicht zu	Trifft sicher zu
Trifft nicht zu	Trifft zu	Trifft nicht zu	Trifft zu
Trifft nicht zu	Trifft zu	Unentschieden	Trifft zu
Trifft sicher nicht zu	Unentschieden	Trifft zu	Trifft sicher zu
Trifft sicher nicht zu	Trifft zu	Trifft zu	Trifft sicher zu
Trifft sicher nicht zu	Trifft sicher zu	Trifft nicht zu	Trifft sicher zu
Trifft sicher nicht zu	Unentschieden	Trifft nicht zu	Trifft sicher zu
Unentschieden	Trifft zu	Trifft sicher nicht zu	Trifft zu
Trifft sicher nicht zu	Trifft zu	Trifft nicht zu	Trifft sicher zu
Trifft sicher nicht zu	Trifft sicher zu	Unentschieden	Trifft sicher zu
Trifft nicht zu	Trifft sicher zu	Unentschieden	Trifft sicher zu
Trifft sicher nicht zu	Trifft zu	Unentschieden	Trifft sicher zu
	Unentschieden	Trifft sicher nicht zu	Unentschieden
Trifft sicher nicht zu	Trifft sicher zu	Trifft nicht zu	Trifft sicher zu
Unentschieden	Trifft sicher zu	Trifft nicht zu	Trifft sicher zu
Trifft nicht zu	Unentschieden	Unentschieden	Trifft zu
Trifft sicher nicht zu	Trifft sicher zu	Trifft nicht zu	Trifft sicher zu
Trifft nicht zu	Trifft sicher zu	Trifft nicht zu	Trifft sicher zu
	Trifft zu		
Trifft nicht zu	Trifft zu	Trifft nicht zu	Trifft sicher zu
Trifft sicher nicht zu	Trifft sicher zu	Trifft nicht zu	Trifft zu
	Trifft zu	Trifft zu	Trifft sicher zu
Trifft sicher nicht zu	Unentschieden	Unentschieden	Trifft sicher zu
Trifft sicher nicht zu	Trifft zu	Unentschieden	Trifft sicher zu
Unentschieden	Trifft sicher zu	Unentschieden	Trifft sicher zu
Trifft sicher nicht zu	Trifft sicher zu	Trifft sicher nicht zu	Trifft zu
Trifft sicher nicht zu	Unentschieden	Trifft sicher nicht zu	Unentschieden
Trifft sicher nicht zu	Unentschieden	Trifft nicht zu	Trifft sicher zu
Trifft sicher nicht zu	Trifft sicher zu	Unentschieden	Trifft sicher zu
Trifft sicher nicht zu	Trifft sicher zu	Trifft sicher nicht zu	Trifft sicher zu
Trifft sicher nicht zu	Unentschieden	Trifft zu	Trifft sicher zu
Trifft nicht zu	Trifft zu	Trifft zu	Trifft zu
Trifft sicher nicht zu	Trifft sicher zu	Unentschieden	Trifft sicher zu

Trifft nicht zu	Trifft zu	Trifft zu	Trifft zu
Trifft sicher nicht zu	Trifft zu	Unentschieden	Trifft zu
Trifft sicher nicht zu	Trifft sicher zu	Trifft zu	Trifft sicher zu
Trifft sicher nicht zu	Trifft sicher zu	Trifft nicht zu	Trifft sicher zu
Trifft sicher nicht zu	Trifft sicher zu	Trifft nicht zu	Trifft zu
Trifft sicher nicht zu	Trifft zu	Unentschieden	Trifft sicher zu
Trifft nicht zu	Trifft zu	Unentschieden	Trifft sicher zu
Trifft sicher zu	Trifft sicher zu	Trifft nicht zu	Trifft sicher zu
Trifft sicher nicht zu	Trifft sicher zu	Trifft sicher nicht zu	Trifft sicher zu
Trifft sicher nicht zu	Trifft sicher zu	Trifft nicht zu	Trifft sicher zu
Trifft sicher nicht zu	Trifft sicher zu	Trifft sicher nicht zu	Trifft sicher zu
Trifft sicher nicht zu	Trifft sicher zu	Trifft nicht zu	Trifft sicher zu
Trifft sicher nicht zu	Unentschieden	Trifft nicht zu	Trifft sicher zu
Trifft nicht zu	Unentschieden	Trifft nicht zu	Unentschieden
Trifft sicher nicht zu	Trifft sicher zu	Unentschieden	Trifft sicher zu
Trifft sicher nicht zu	Unentschieden	Trifft nicht zu	Trifft sicher zu
Trifft sicher nicht zu	Trifft sicher zu	Trifft sicher nicht zu	Trifft sicher zu
Trifft sicher nicht zu	Trifft sicher zu	Trifft nicht zu	Trifft sicher zu
Trifft sicher nicht zu	Trifft sicher zu	Trifft nicht zu	Trifft sicher zu
Trifft sicher nicht zu	Trifft sicher zu	Trifft zu	Trifft sicher zu
Trifft sicher nicht zu	Trifft zu	Trifft nicht zu	Trifft zu
Trifft sicher nicht zu	Trifft sicher zu	Trifft nicht zu	Trifft sicher zu
Trifft nicht zu	Unentschieden	Unentschieden	Trifft zu
Trifft sicher nicht zu	Trifft sicher zu	Trifft sicher nicht zu	Trifft zu
Trifft nicht zu	Trifft sicher zu	Unentschieden	Trifft zu
Trifft sicher nicht zu	Trifft zu	Unentschieden	Unentschieden
Trifft nicht zu	Trifft sicher zu	Unentschieden	Trifft sicher zu
Trifft sicher nicht zu	Unentschieden	Trifft sicher nicht zu	Trifft zu
Trifft nicht zu	Unentschieden	Unentschieden	Trifft sicher zu
	Trifft zu	Trifft nicht zu	Trifft sicher zu
Trifft sicher nicht zu	Trifft nicht zu	Trifft zu	Trifft sicher zu
Trifft sicher nicht zu	Trifft sicher zu	Trifft zu	Trifft sicher zu
Trifft sicher nicht zu	Trifft zu	Unentschieden	Trifft sicher zu
Trifft sicher nicht zu	Trifft zu	Trifft zu	Trifft sicher zu
Trifft nicht zu	Trifft zu	Unentschieden	Trifft sicher zu
Trifft nicht zu	Trifft zu	Unentschieden	Trifft zu
Trifft sicher nicht zu	Trifft zu	Unentschieden	Trifft zu
Trifft sicher nicht zu	Trifft sicher zu	Trifft nicht zu	Trifft sicher zu
Trifft sicher nicht zu	Unentschieden	Trifft nicht zu	Trifft zu
Trifft sicher nicht zu	Trifft sicher zu	Trifft nicht zu	Trifft sicher zu
Trifft sicher nicht zu	Trifft sicher zu	Unentschieden	Trifft zu
Trifft nicht zu	Trifft zu	Unentschieden	Trifft zu
Trifft sicher nicht zu	Trifft sicher zu	Trifft nicht zu	Trifft zu
Trifft sicher nicht zu	Trifft zu	Trifft nicht zu	Trifft zu
Trifft sicher nicht zu	Trifft sicher zu	Trifft nicht zu	Unentschieden
Trifft sicher nicht zu	Unentschieden	Trifft nicht zu	Trifft sicher zu
Unentschieden	Trifft zu	Unentschieden	Unentschieden
Unentschieden	Trifft sicher zu	Unentschieden	Trifft zu
	Trifft zu	Unentschieden	Trifft sicher zu
Trifft sicher nicht zu	Unentschieden	Trifft sicher nicht zu	Trifft nicht zu

Trifft sicher nicht zu	Trifft zu	Unentschieden	Trifft zu
Trifft nicht zu	Unentschieden	Trifft zu	Trifft zu
Trifft nicht zu	Trifft sicher zu	Unentschieden	Trifft zu
Trifft sicher nicht zu	Trifft sicher nicht zu	Trifft zu	Trifft sicher zu
Trifft sicher nicht zu	Unentschieden	Trifft sicher nicht zu	Trifft sicher zu
Trifft sicher nicht zu	Trifft sicher zu	Trifft nicht zu	Trifft sicher zu
Trifft sicher nicht zu	Trifft zu	Trifft sicher nicht zu	Trifft nicht zu
Trifft nicht zu	Trifft zu	Unentschieden	Trifft sicher zu
Trifft sicher nicht zu	Trifft zu	Unentschieden	Trifft zu
Trifft sicher nicht zu	Unentschieden	Trifft nicht zu	Trifft sicher zu
Trifft sicher nicht zu	Unentschieden	Unentschieden	Trifft zu
Trifft nicht zu	Trifft zu	Unentschieden	Unentschieden
Trifft zu	Unentschieden	Trifft zu	Trifft sicher zu
Trifft sicher nicht zu	Unentschieden	Trifft zu	Trifft sicher zu
Trifft sicher nicht zu	Trifft sicher nicht zu	Trifft zu	Trifft zu
Trifft sicher nicht zu	Unentschieden	Unentschieden	Trifft zu
Trifft nicht zu	Trifft zu	Trifft nicht zu	Unentschieden
Trifft sicher nicht zu	Trifft zu	Unentschieden	Trifft sicher zu
Trifft sicher nicht zu	Unentschieden	Trifft sicher nicht zu	Trifft sicher zu
Trifft nicht zu	Trifft zu	Trifft nicht zu	Trifft zu
Trifft sicher nicht zu	Trifft zu	Trifft sicher nicht zu	Unentschieden
Trifft sicher nicht zu	Trifft zu	Trifft nicht zu	Trifft nicht zu
Trifft sicher nicht zu	Trifft sicher zu	Trifft nicht zu	Unentschieden
Trifft sicher nicht zu	Trifft sicher zu	Trifft zu	Trifft sicher zu
Trifft sicher nicht zu	Trifft sicher zu	Unentschieden	Trifft zu
Trifft sicher nicht zu	Trifft sicher zu	Trifft sicher nicht zu	Trifft sicher zu
Trifft sicher nicht zu	Unentschieden	Trifft zu	Trifft zu
Trifft sicher nicht zu	Trifft sicher zu	Trifft nicht zu	Trifft zu
Trifft sicher nicht zu	Trifft zu	Trifft sicher zu	Trifft sicher zu
Trifft sicher nicht zu	Unentschieden	Unentschieden	Trifft zu
Trifft nicht zu	Trifft zu	Unentschieden	Trifft zu
Trifft sicher nicht zu	Trifft sicher zu	Trifft sicher nicht zu	Trifft sicher zu
Trifft nicht zu	Trifft zu	Unentschieden	Trifft zu
Trifft nicht zu	Trifft zu	Unentschieden	Trifft sicher zu
Trifft nicht zu	Trifft sicher zu	Unentschieden	Trifft zu
Trifft sicher nicht zu	Trifft nicht zu	Trifft sicher nicht zu	Trifft sicher zu
Trifft nicht zu			
Trifft nicht zu	Trifft zu	Trifft zu	Trifft sicher zu
Trifft sicher nicht zu	Trifft zu	Trifft nicht zu	Trifft sicher zu
Trifft sicher nicht zu	Trifft zu	Trifft sicher nicht zu	Trifft sicher zu
Trifft sicher nicht zu	Trifft zu	Unentschieden	Trifft sicher zu
Trifft nicht zu	Trifft sicher zu	Trifft nicht zu	Trifft sicher zu
Trifft nicht zu	Trifft zu	Trifft zu	Trifft sicher zu
Trifft nicht zu	Trifft sicher zu	Trifft nicht zu	Trifft sicher zu
Unentschieden	Unentschieden	Trifft nicht zu	Trifft zu
Trifft sicher nicht zu	Trifft sicher zu	Unentschieden	Trifft sicher zu
Trifft sicher nicht zu	Trifft zu	Trifft nicht zu	Trifft sicher zu
Trifft sicher nicht zu	Trifft sicher zu	Trifft nicht zu	Trifft zu
Trifft nicht zu	Unentschieden	Unentschieden	Trifft sicher zu

Trifft nicht zu	Trifft zu	Trifft zu	Trifft sicher zu
Trifft nicht zu	Trifft zu	Trifft zu	Trifft zu
Trifft sicher nicht zu	Trifft sicher zu	Trifft nicht zu	Trifft zu
Trifft sicher nicht zu	Unentschieden	Trifft sicher nicht zu	Trifft nicht zu
Trifft nicht zu	Trifft sicher zu	Unentschieden	Trifft sicher zu
Trifft nicht zu	Trifft zu	Trifft zu	Trifft sicher zu
Trifft sicher nicht zu	Trifft sicher zu	Trifft nicht zu	Trifft sicher zu
Trifft sicher nicht zu	Trifft sicher zu	Trifft nicht zu	Trifft sicher zu
Trifft sicher nicht zu	Unentschieden	Unentschieden	Trifft sicher zu
Trifft sicher zu	Trifft sicher zu	Trifft sicher nicht zu	Trifft sicher zu
Trifft sicher nicht zu	Trifft zu	Trifft sicher zu	Trifft sicher zu
Trifft sicher nicht zu	Trifft sicher zu	Trifft zu	Trifft sicher zu
Trifft nicht zu	Trifft sicher zu	Trifft nicht zu	Trifft zu
Trifft sicher nicht zu	Trifft sicher zu	Trifft zu	Trifft sicher zu
Unentschieden	Unentschieden	Trifft zu	Trifft zu
Trifft nicht zu	Trifft sicher zu	Unentschieden	Trifft sicher zu
Trifft sicher nicht zu	Trifft zu	Unentschieden	Trifft sicher zu
Trifft nicht zu	Trifft sicher zu	Unentschieden	Trifft sicher zu

Ich würde mir mehr stuc	Durch die studentische l	Ich würde den Besuch di	Im Vergleich zu herkömı
Trifft zu	Unentschieden	Trifft zu	
Trifft zu	Trifft zu	Trifft zu	
Trifft zu	Trifft sicher zu	Trifft zu	
Trifft zu	Trifft nicht zu	Trifft nicht zu	
Trifft sicher zu	Unentschieden	Trifft sicher zu	
Trifft sicher zu	Trifft sicher zu	Trifft sicher zu	
Trifft zu	Trifft sicher zu	Trifft zu	
Trifft zu	Trifft zu	Trifft zu	
Unentschieden	Unentschieden	Trifft zu	
Unentschieden	Trifft zu	Unentschieden	
Trifft zu	Trifft sicher zu	Trifft sicher zu	
Unentschieden	Trifft sicher zu	Trifft nicht zu	
Trifft sicher zu	Unentschieden	Unentschieden	
Unentschieden	Unentschieden	Trifft nicht zu	
Trifft sicher zu	Trifft sicher zu	Trifft sicher zu	
Trifft zu	Trifft zu	Trifft sicher zu	
Trifft sicher zu	Trifft sicher zu	Trifft zu	
Unentschieden	Unentschieden	Unentschieden	
Trifft zu	Trifft sicher zu	Trifft zu	
Trifft zu	Trifft zu	Trifft sicher zu	
Unentschieden	Trifft nicht zu	Trifft zu	
Trifft zu	Trifft zu	Trifft sicher zu	
Trifft sicher zu	Trifft zu	Unentschieden	
Trifft zu	Trifft zu	Trifft zu	
Unentschieden	Trifft zu	Trifft zu	
Trifft sicher zu	Unentschieden	Trifft sicher zu	
Unentschieden	Trifft zu	Unentschieden	
Trifft zu	Trifft zu	Trifft zu	
Trifft sicher zu	Trifft zu	Trifft zu	
Unentschieden	Trifft zu	Trifft zu	
Trifft sicher zu	Trifft sicher zu		
Unentschieden	Unentschieden	Unentschieden	
Trifft sicher zu	Trifft sicher zu	Trifft sicher zu	
Trifft sicher zu	Trifft sicher zu	Trifft nicht zu	
Trifft sicher zu	Trifft zu	Trifft zu	
Trifft zu	Trifft zu	Unentschieden	
Unentschieden	Trifft sicher zu	Trifft sicher zu	
Unentschieden	Trifft zu	Unentschieden	
Unentschieden	Trifft nicht zu	Trifft zu	
Trifft zu	Unentschieden	Trifft nicht zu	
Unentschieden	Trifft zu	Trifft zu	
Unentschieden	Unentschieden	Unentschieden	
Trifft zu	Trifft zu	Trifft sicher zu	
Unentschieden	Unentschieden	Unentschieden	
Unentschieden	Trifft zu	Trifft sicher zu	
Trifft sicher zu	Trifft sicher zu	Trifft sicher zu	
Unentschieden	Trifft zu	Trifft nicht zu	Trifft nicht zu
Trifft sicher zu	Trifft sicher zu	Trifft sicher zu	Trifft zu
Trifft zu	Unentschieden	Trifft zu	Trifft zu

Trifft zu	Unentschieden	Unentschieden	Unentschieden
Trifft zu	Unentschieden	Trifft zu	Trifft zu
Trifft zu	Trifft zu	Trifft zu	Unentschieden
Trifft sicher zu	Trifft sicher zu	Trifft sicher zu	Trifft nicht zu
Trifft sicher zu	Trifft sicher zu	Trifft sicher zu	Trifft sicher zu
Trifft zu	Trifft sicher zu	Unentschieden	Trifft zu
Trifft zu	Trifft sicher zu	Unentschieden	Trifft zu
Trifft sicher zu	Trifft sicher zu	Trifft sicher zu	Trifft sicher zu
Trifft sicher zu	Trifft sicher zu	Trifft sicher zu	Trifft sicher zu
Trifft zu	Trifft zu	Trifft sicher zu	Trifft sicher zu
Trifft sicher zu	Trifft sicher zu	Trifft sicher zu	Trifft sicher zu
Trifft zu	Trifft sicher zu	Unentschieden	Unentschieden
Trifft zu	Trifft zu	Trifft sicher zu	Unentschieden
Trifft zu	Trifft zu	Unentschieden	Unentschieden
Trifft sicher zu	Trifft sicher zu	Trifft sicher zu	Trifft zu
Trifft zu	Unentschieden	Trifft zu	Trifft zu
Trifft sicher zu	Trifft sicher zu	Trifft sicher zu	Trifft zu
Trifft sicher zu	Trifft sicher zu	Trifft sicher zu	Trifft sicher zu
Trifft sicher zu	Trifft sicher zu	Trifft zu	Trifft zu
Trifft zu	Trifft zu	Trifft zu	Unentschieden
Trifft sicher zu	Unentschieden	Unentschieden	Unentschieden
Trifft sicher zu	Trifft sicher zu	Trifft zu	Trifft sicher zu
Unentschieden	Trifft zu	Unentschieden	Unentschieden
Trifft zu	Trifft zu	Trifft sicher zu	Trifft sicher zu
Trifft sicher zu	Trifft sicher zu	Trifft sicher zu	Trifft sicher zu
Trifft zu	Trifft sicher zu	Unentschieden	Unentschieden
Trifft zu	Trifft zu	Unentschieden	Unentschieden
Trifft zu	Unentschieden	Trifft sicher zu	Unentschieden
Unentschieden	Unentschieden	Trifft sicher zu	Trifft sicher zu
Trifft zu		Trifft sicher zu	
Unentschieden	Trifft zu	Unentschieden	Unentschieden
Trifft sicher zu	Trifft sicher zu	Trifft sicher zu	Trifft sicher zu
Trifft zu	Trifft zu	Trifft zu	Trifft zu
Trifft sicher zu	Trifft sicher zu	Trifft zu	Trifft zu
Unentschieden	Unentschieden	Trifft nicht zu	Trifft nicht zu
Trifft zu	Trifft zu	Trifft zu	Trifft zu
Trifft zu	Trifft sicher zu	Trifft sicher zu	Trifft sicher zu
Unentschieden	Trifft zu	Trifft nicht zu	Trifft sicher zu
Unentschieden	Trifft zu	Trifft zu	Trifft zu
Trifft zu	Trifft sicher zu	Trifft zu	Trifft zu
Trifft sicher zu	Trifft sicher zu	Trifft sicher zu	Trifft sicher zu
Trifft zu	Trifft zu	Trifft zu	Trifft zu
Trifft sicher zu	Trifft sicher zu	Trifft zu	Trifft sicher zu
Trifft zu	Trifft zu	Trifft zu	Unentschieden
Trifft sicher zu	Trifft sicher zu	Unentschieden	Trifft zu
Trifft nicht zu	Trifft nicht zu	Trifft sicher zu	Trifft zu
Trifft zu	Trifft zu	Trifft zu	Unentschieden
Trifft zu	Trifft zu	Unentschieden	Unentschieden
Trifft zu	Trifft zu	Trifft zu	Trifft zu
Unentschieden	Unentschieden	Trifft sicher zu	Trifft sicher zu

Trifft zu	Trifft zu	Unentschieden	Trifft zu
Unentschieden	Unentschieden	Unentschieden	Unentschieden
Trifft zu	Trifft zu	Trifft zu	Trifft zu
Trifft sicher zu	Trifft sicher nicht zu	Trifft sicher zu	Trifft sicher nicht zu
Unentschieden	Unentschieden	Trifft sicher zu	Unentschieden
Trifft zu	Trifft zu	Unentschieden	Unentschieden
Trifft sicher zu	Trifft zu	Trifft sicher zu	Trifft sicher zu
Trifft sicher zu	Trifft sicher zu	Trifft sicher zu	Trifft sicher zu
Trifft zu	Trifft zu	Unentschieden	Unentschieden
Unentschieden	Trifft zu	Trifft sicher zu	Trifft zu
Trifft zu	Unentschieden	Trifft zu	Trifft zu
Trifft zu	Trifft zu	Unentschieden	Unentschieden
Unentschieden	Unentschieden	Trifft sicher zu	Unentschieden
Unentschieden	Unentschieden	Unentschieden	Trifft zu
Trifft nicht zu	Trifft sicher nicht zu	Trifft sicher nicht zu	Trifft nicht zu
Unentschieden	Unentschieden	Trifft nicht zu	Unentschieden
Unentschieden	Unentschieden	Unentschieden	Unentschieden
Unentschieden	Trifft zu	Unentschieden	Unentschieden
Trifft zu	Trifft zu	Trifft sicher zu	Trifft sicher zu
Trifft sicher zu	Trifft zu	Trifft sicher zu	Trifft sicher zu
Trifft zu	Trifft zu	Trifft sicher zu	Trifft sicher zu
Unentschieden	Trifft zu	Trifft sicher zu	Trifft sicher zu
Trifft zu	Trifft zu	Trifft zu	Trifft sicher zu
Unentschieden	Trifft sicher zu	Unentschieden	Trifft zu
Unentschieden	Trifft zu	Trifft zu	Trifft zu
Trifft sicher zu	Trifft sicher zu	Trifft sicher zu	Trifft sicher zu
Unentschieden	Unentschieden	Unentschieden	Unentschieden
Trifft zu	Trifft zu	Trifft sicher zu	Trifft zu
Trifft nicht zu	Unentschieden	Unentschieden	Trifft zu
Trifft zu	Trifft zu	Unentschieden	Unentschieden
Trifft zu	Trifft zu	Trifft zu	Trifft zu
Trifft sicher zu	Trifft sicher zu	Trifft sicher zu	Trifft sicher zu
Trifft zu	Trifft zu	Trifft zu	Trifft zu
Unentschieden	Trifft zu	Unentschieden	Unentschieden
Trifft zu	Trifft zu	Trifft zu	Unentschieden
Trifft sicher zu	Trifft nicht zu	Trifft sicher zu	Trifft sicher zu
Trifft zu	Trifft zu	Trifft zu	Trifft zu
Trifft zu	Trifft zu	Unentschieden	Trifft zu
Unentschieden	Unentschieden	Trifft zu	Trifft zu
Trifft zu	Trifft zu	Trifft zu	Unentschieden
Trifft zu	Trifft sicher zu	Trifft sicher zu	Trifft sicher zu
Trifft zu	Trifft zu	Trifft sicher zu	Trifft sicher zu
Trifft zu	Trifft sicher zu	Trifft zu	Trifft zu
Unentschieden	Unentschieden	Unentschieden	Trifft zu
Trifft sicher zu	Trifft sicher zu	Unentschieden	Trifft zu
Unentschieden	Trifft zu	Unentschieden	Trifft sicher zu
Trifft sicher zu	Trifft sicher zu	Trifft sicher zu	Trifft sicher zu
Unentschieden	Trifft zu	Trifft nicht zu	Trifft nicht zu

Trifft zu	Trifft zu	Unentschieden	Trifft zu
Unentschieden	Trifft zu	Trifft zu	Trifft zu
Trifft sicher nicht zu	Trifft sicher zu	Trifft sicher zu	Trifft zu
Trifft zu	Unentschieden	Trifft zu	Trifft zu
Trifft zu	Trifft sicher zu	Trifft sicher zu	Unentschieden
Trifft sicher zu	Trifft sicher zu	Trifft sicher zu	Trifft sicher zu
Trifft sicher zu	Trifft sicher zu	Trifft sicher zu	Trifft sicher zu
Trifft sicher zu	Trifft sicher zu	Trifft sicher zu	Trifft sicher zu
Trifft sicher zu	Unentschieden	Trifft sicher zu	Trifft zu
Trifft sicher zu	Trifft sicher zu	Unentschieden	Unentschieden
Trifft nicht zu	Trifft zu	Trifft zu	Trifft sicher zu
Trifft zu	Trifft sicher zu	Trifft sicher zu	Trifft sicher zu
Trifft zu	Trifft zu	Trifft sicher zu	Trifft sicher zu
Unentschieden	Unentschieden	Trifft sicher zu	Trifft sicher zu
Trifft zu	Unentschieden	Trifft zu	Trifft sicher zu
Trifft sicher zu	Trifft sicher zu	Trifft zu	Unentschieden
Trifft sicher zu	Trifft nicht zu	Trifft nicht zu	Trifft zu
Trifft zu	Trifft sicher zu	Trifft sicher zu	Trifft zu

Sonst noch Feedback, da
Weiter so! War echt cool, vielen Dank!
Sehr gute, spannende und lehrreiche Veranstaltung!!!
Ein wirklich ganz tolles Seminar!
Super gemacht und ein tolles neues Format!
Wäre sehr hilfreich wenn es das bald als wahlfach gäbe.
Vielen Dank für Euer Engagement! Hat viel Spaß gemacht:)
Sehr spannende und lehrreiche Veranstaltung :)
Unglaubliche tolles Format ! Wäre eine Schande, wenn dieses Seminar nicht zum offiziellen Wahlfach scha
Der Fall war spannend, aber doch sehr speziell und lang. Vielleicht lieber drei kleinere, alltäglichere Fälle a
Es war sehr interessant und extrem gut gemacht von euch! Obwohl ich noch nicht mal in der Klinik bin ko
Sehr gerne weiter so. Es hat Spaß gemacht und war lehrreich
Ich fand sehr gut dass viel von dem was Kommilitonen geschrieben haben nochmal erklärt wurde. Ich kanı
Es hat richtig viel Spaß gemacht und ich habe viel gelernt, danke!:)
Mir hat die Veranstaltung sehr viel gebracht. Die zum klassischen Studium oft "umgekehrte" Reihenfolge h
Super Veranstaltung und zur Abwechslung endlich etwas Nähe zur Praxis!Werde ich definitiv auch weiter b
Fand es super! Hat echt Spaß gemacht! Danke fürs Engagement!

Find euer Seminar echt super, die Interaktion ist sehr gut (auch dass wir kein Mikro anmachen müssen :D)

Tausend Dank!!!

Richtig cool 😉 danke

Ich würde mich sehr freuen, wenn ihr die Folien teilen könnte, zumindest die Wissensfolie, zum Beispiel h[illegible]

Antwortzeit bitte länger

Ich würde mir wünschen , dass die Fragen am Anfang als Warm up beibehalten werden. Aber mindestens [illegible]

Manches kann ich noch nicht beurteilen, weil ich Ersti bin :-D deshalb so oft Unentschieden angekreuzt

Cool dass ihr das macht!

Bin großer Fan der Veranstaltung

Bitte bei Polleverywhere die Antwortzeit von 15 Sekunden auf 20 oder 25 Sekunden verlängern.

Etwas mehr Zeit zur Beantwortung der Fragen. Vlt 30 Sekunden anstatt von 15. Hat echt Spaß gemacht :D

Ich bin aus der Vorklinik und kann meistens mit den vielen Eigennamen nichts anfangen. Mir ist klar, dass [illegible]

Es war auch für mich als Erstsemester sehr interessant, auch mit medizinischer Vorerfahrung, war es span[illegible]

Vielen Dank Euch, dass Ihr diese Veranstaltung anbietet!

Ich denke es wäre förderlich für die Motivation und auch Konzentration, wenn das Seminar auf eine Stund[illegible]

Das Tempo ist schnell- mir zu schnell, zB beim Durchgehen von Werten, beantworten von fragen, und teilw[illegible]

Ich würde mir teilweise mehr Grundlagenerklärung wünschen. Zwar ist mir bewusst, dass die Veranstaltur
Super Veranstaltung!

Danke für eure Mühe, es war sehr hilf- und lehrreich! Bin immer gerne wieder dabei :)

Ich find's super! Weiter so

gerne weiterhin per email erinnern einen tag vorhergerne auch öfter und auch in den ferien machen :)

Weiter so :) einzelne Blickdiagnosen habe ich persönlich vorher noch nie gesehen oder gehört, aber ich fin

Vielen Dank für die Vorstellung dieser spannenden Fälle!
Sehr interessanter Fall heute! Super Seminar!

Vielleicht eine Option, dass man die wichtigsten Facts für den besprochenen Fall, auch wenn man später d

Einfach genial, macht super viel Spaß, ich kann super viel mitnehmen und finde das aufjedenfall ein Angeb

Fand die Fälle bisher allesamt interessant und im allgemeinen das Konzept der Veranstaltung wirklich toll!
Macht ihr richtig gut!
Wir kannten den Fall durch Prof. Schäfer im bzw. vor dem ersten vorkl. Semester, haben uns nur nicht glei
Ich bedanke mich für die zwei Studierenden, die sich dafür Zeit genommen haben. Danke
Könnte mir vorstellen, diese Veranstaltung oder ähnliche freiwillig 2-3 Mal die Woche mitzumachen =) Vllt
Es gibt nix zu meckern, die Fälle sind spannend und ihr leitet das super! Der Dienstag Abend ist für mich pe

Bei mir hat die Software zum auswählen leider nicht funktioniert aber vielleicht geht es auch nur am PC. A

Tolles Engagement! Vielen Dank an alle Beteiligte
Vielen Dank für eure Mühe!!!
Nice, Danke dafür! Macht so weiter!
Ich wäre nächste Woche dabei :)
Vielen Dank. Ihr macht das sehr gut. Bin immer wieder gerne dabei :)
Das war eine großartige Sache und ich hoffe, dass ihr das ihr Zukunft weiterführen könnt. Ich nehme gern

us einem Themengebiet der Medizin, dazu dann noch passende Blickdiagnosen? Ansonsten super Veran

nnte ich halbwegs gut folgen und ihr habt es geschafft, das Niveau so zu halten, dass alle etwas davon m

ilft mir sehr beim Vernetzen von den verschiedenen Inhalten. Mir ist schon öfter in praktischen Situatione

. Man kann sich gut konzentrieren und eure Folien sind didaktisch super aufgearbeitet. Das Quiz am Anfa

eute Ursachen für Wesensveränderungen und sowas, dann muss man nicht alles mitschreiben oder foto

5 Sekunden pro Antwortoption Zeit zu haben, zu lange sollte es aber auch nicht sein um die Spontanität i

ihr nicht alles nochmal definieren könnt, aber vllt ja manchmal wenn es um wichtige Tests geht, die wich

veise auch den Erläuterungen. Lieber weniger Details damit mehr hängenbleibt und ich besser folgen kan

ıg für etwas fortgeschrittene Kliniker konzipiert wurde, allerdings würden teilweise Grundlagenerläuteru

lazu stößt, auch dann noch einsehen kann. Ansonsten kann man nicht wirklich mitmachen, wenn man be

Das Studium bräuchte viel mehr Veranstaltungen solcher Art, weil diese viel praxisorientierter sind. Viel

gibt es ja bald mehr studentische Lehre ;)Die herkömmlichen Seminare sind jedoch - wie ihr selbst sagt -
ersönlich leider sehr ungünstig, weshalb ich in Zukunft wahrscheinlich nicht mehr teilnehmen kann. Aber

itnehmen, egal welches Semester. Meine persönliche „Meinung“ ist natürlich, dass ich an der einen oder

en aufgefallen, dass mir die Transferleistung von Symptom -> Diagnose schwerer fällt als anders herum.A

ang hat Spaß gemacht. Ein erfahrener Arzt fehlt mir persönlich nicht. Die Fälle sind zwar eher selten, aber

grafieren und kann sich das im Nachhinein nochmal in Ruhe anschauen oder mal nachlesen, falls man es

ɛispielsweise den Anfang mit Anamnese verpasst hat und keine Ahnung hat welche Symptome vorrangig

wegen der beruflichen Erfahrung der Kliniker weiterhin essentiell, auch wenn die einzelnen Seminare nic

r anderen Stelle manchmal ganz kurze Erklärungen vermisst habe von Dingen, die ich als Vorklinikerin na

ußerdem war die Veranstaltung sehr gut und strukturiert vorbereitet mit einem spannenden Fall. Und vo

r es ist auch gut, etwas über seltene Krankheiten zu erfahren (wenn man an das IMPP denkt :D). Macht v

:ht an euren Aufwand/Technik für die einzelne Veranstaltung herankommenPS: Case war super =)

türlich noch nicht wissen kann. Trotzdem ist mir klar, dass ihr darauf nur bedingt eingehen könnt, weil es

n Studenten lernen sehe ich (als 7 Sem) gar nicht als problematisch an, von euch kann ich super viel lerne

s nunmal eine klinische Veranstaltung ist und es wahrscheinlich den Rahmen sprengen würde wenn man

en. Klar ist es gut, dass zur "Sicherheit" ein Experte anwesend ist, aber für mich persönlich wäre das nicht

noch so viel zusätzlich erklären würde. Freue mich aufs nächste Mal!
