# Supplementary material for: Teaching medicine web-based with the help of interactive audience response systems
Source: PLoS One. 2023 Aug 15;18(8):e0289417. doi: 10.1371/journal.pone.0289417 (PMC10427006; doi:10.1371/journal.pone.0289417)
Supplement: S3 File — (PDF) [file pone.0289417.s003.PDF]

Evaluation zur Lehrveranstaltung "Vom Symptom zur Diagnose"

Das Ausfüllen der Umfrage dauert ungefähr 7 Minuten.

* Erforderlich

1. In welchem Studienjahr befindest du dich aktuell? *

- ◯ 1
- ◯ 2
- ◯ 3
- ◯ 4
- ◯ 5
- ◯ 6
- ◯ Sonstiges

2. Wie viele Famulaturen hast du bisher absolviert? *

- ◯ 1
- ◯ 2
- ◯ 3
- ◯ 4
- ◯ 5
- ◯ 6
- ◯ Mehr als 6
- ◯ Keine
- ◯ Sonstiges

3. Was sind Gründe für eure Teilnahme an der Veranstaltung "Vom Symptom zur Diagnose"?

	Trifft sicher zu	Trifft zu	Unentschie den	Trifft nicht zu	Trifft sicher nicht zu
Die Inhalte sind relevant für meine Ausbildung.	◯	◯	◯	◯	◯
Für mich ist der Zeitpunkt, früher Abend, angenehm.	◯	◯	◯	◯	◯
Um mehr über gezieltes diagnostische s Vorgehen beim erörtern einer Diagnose zu erlernen.	◯	◯	◯	◯	◯
Um mehr diagnostische Möglichkeite n kennenzulern en.	◯	◯	◯	◯	◯
Um neue Krankheitsbil der zu entdecken.	◯	◯	◯	◯	◯
Das Event hat mir Spaß gemacht.	◯	◯	◯	◯	◯
Ich habe dort viel gelernt.	◯	◯	◯	◯	◯
Ich habe dort Neues gelernt.	◯	◯	◯	◯	◯
Ich möchte gerne klinisch wichtige Blickdiagnose n erlernen.	◯	◯	◯	◯	◯
Differentialdi agnostisches Denken und "Rätseln" bereit mir Freude.	◯	◯	◯	◯	◯

4. Wie viel Erfahrung hast du mit ...

	Gar keine	Etwas	Ab und zu	Häufig	Sehr viel
Der Durchführung einer klinischen Untersuchung.	○	○	○	○	○
Der Beurteilung von Laborbefunden.	○	○	○	○	○
Der Beurteilung einer Bildgebung (CT/MRT/Röntgen).	○	○	○	○	○
Dem Lösen sogenannter Blickdiagnosen.	○	○	○	○	○
Der Beurteilung, welche Diagnostik in der Klinik zu Anfang gewählt werden sollte.	○	○	○	○	○
Der Beurteilung von bedrohlichen Patientensituationen, die dringendes Handeln erfordern.	○	○	○	○	○

5. Wie ist dein Gesamteindruck der Lehrveranstaltung?

	Trifft sicher zu	Trifft zu	Unentschieden	Trifft nicht zu	Trifft sicher nicht zu
Der Anspruch der Veranstaltung ist angemessen.	○	○	○	○	○
Der Besuch der Lehrveranstaltung war für mich mit einem Lernerfolg verbunden.	○	○	○	○	○
Die Auswahl und Lösbarkeit des Falls würde ich als gut bewerten.	○	○	○	○	○
Durch die Veranstaltung fällt es mir leichter, einzelne Symptome Krankheitsbildern zuzuordnen.	○	○	○	○	○
Das Erlernen von Blickdiagnosen kann für den späteren klinischen Alltag von Vorteil sein.	○	○	○	○	○
Die Lösbarkeit der Blickdiagnosen würde ich als gut bzw. machbar bewerten.	○	○	○	○	○
Ich würde die Veranstaltung meinen Kommiliton:innen weiterempfehlen.	○	○	○	○	○
Die Veranstaltung hat für mich keinen großen Mehrwert.	○	○	○	○	○
Der zeitliche Umfang der Veranstaltung ist angemessen.	○	○	○	○	○
Ich würde mir wünschen, dass die Veranstaltung Teil der curriculären Lehre wird.	○	○	○	○	○
Die studentische Leitung konnte durch fundiertes Wissen überzeugen und Fragen gezielt beantworten.	○	○	○	○	○
Das Seminar war professionell und didaktisch sinnvoll.	○	○	○	○	○

6. In welchem umfang treffen die folgenden Aussagen auf dich zu?

	Trifft sicher zu	Trifft zu	Unentschieden	Trifft nicht zu	Trifft sicher nicht zu
Während des Seminars fiel es mir leicht mich durchgehend zu konzentrieren.	○	○	○	○	○
Ich befürworte es, dass im Seminar relevante Inhalte thematisch aufgegriffen und erklärt werden.	○	○	○	○	○
Nach Teilnahme an dieser Veranstaltung ist mir aufgefallen, dass die Lehre ausgehend vom Symptom im Medizinstudium nicht ausreichend behandelt wird.	○	○	○	○	○
Ich konnte bereits neue Krankheitsbilder und Blickdiagnosen erlernen	○	○	○	○	○
Durch das Seminar fällt es mir leichter anhand verschiedener Symptome zu beurteilen, welche Differentialdiagnosen in Frage kommen.	○	○	○	○	○
Das Erlernen von Symptomen bzw. Symptomkonstellationen fördert mein differentialdiagnostisches Denken.	○	○	○	○	○

7. Wie würdet ihr die Nutzung des Tools "Polleverywhere" beurteilen?

	Trifft sicher zu	Trifft zu	Unentschieden	Trifft nicht zu	Trifft sicher nicht zu
Durch das Tool wurde das Seminar deutlich interaktiver.	○	○	○	○	○
Ich würde mir die Nutzung des Tools weiterhin wünschen.	○	○	○	○	○
Ich würde mir die Nutzung auch in anderen Veranstaltung wünschen.	○	○	○	○	○
Ein Seminar ohne die Verwendung zusätzlicher Tools würde ich bevorzugen.	○	○	○	○	○

8. Was sind Vor- und Nachteile es studentisch geführten Seminars?

	Trifft sicher zu	Trifft zu	Unentschieden	Trifft nicht zu	Trifft sicher nicht zu
Ein Vorteil der Lehre durch Studierende ist die niedrigschwellige Möglichkeit des Nachfragens, wovor viele bei den Profs eher Hemmungen haben.	○	○	○	○	○
Ein Nachteil der Lehre durch Studierende ist deren fehlende berufliche Erfahrung und dass die Lehrinhalte angelesen und bestenfalls in der Vorbereitung durch den Dozenten besprochen werden.	○	○	○	○	○
Um die fehlende klinische Erfahrung zu kompensieren werden wir bei den Seminaren stets einen "altgedienten Kliniker/in" mit dabeihaben. Ist dies aus Eurer Sicht hilfreich?	○	○	○	○	○
Ich würde mir mehr studentisch geleitete Veranstaltungen wünschen.	○	○	○	○	○
Durch die studentische Leitung fällt mir die Interaktion leichter.	○	○	○	○	○
Ich würde den Besuch dieser Veranstaltung gegenüber herkömmlichen Seminaren bevorzugen.	○	○	○	○	○
Im Vergleich zu herkömmlichen Seminaren ist diese Veranstaltung für mich mit einem größeren Lerneffekt verbunden.	○	○	○	○	○

9. Sonst noch Feedback, das du uns mitteilen möchten?

Dieser Inhalt wurde von Microsoft weder erstellt noch gebilligt. Die von Ihnen übermittelten Daten werden an den Formulareigentümer gesendet.

Microsoft Forms
